# Novel metallo-supramolecular architectures based on side-pyridine-modified terpyridines: design, self-assembly, and properties

**DOI:** 10.1039/d5sc06054k

**Published:** 2025-10-06

**Authors:** Ningxu Han, Xin Jiang, Ming Wang

**Affiliations:** a State Key Laboratory of Supramolecular Structure and Materials, College of Chemistry, Jilin University Changchun Jilin 130012 China mingwang358@jlu.edu.cn; b Department of Chemistry, National University of Singapore Singapore 117543 Singapore xinjiang@nus.edu.sg

## Abstract

2,2′:6′,2′′-Terpyridine (tpy), as a classical tridentate chelating ligand, has been extensively utilized to construct discrete metallo-supramolecular architectures. However, current research has predominantly focused on conventional modification strategies at the 4′-position of the central pyridine ring. Recent breakthroughs in synthetic methodologies for side-pyridine functionalization have given rise to a new generation of tpy-based metallo-supramolecular systems. These innovative achievements have transcended the limitations of traditional design paradigms by significantly improving the coordination selectivity of terpyridine systems, thereby substantially expanding both the structural diversity and functional dimensionality of tpy-based coordination chemistry. This review systematically summarizes recent advances in novel side-pyridine-modified tpy-based coordination supramolecular architectures, including mononuclear complexes, helicates, two-dimensional (2D) macrocycles and polygons, three-dimensional (3D) cages and polyhedra, and decker architectures. A comprehensive discussion is provided on the functional implementations of these systems across various domains, particularly highlighting their applications in luminescence, catalysis, chirality, host–guest chemistry, and hierarchical self-assembly.

## Abstract 2

This review summarizes how novel side-pyridine functionalization of terpyridine-based ligands overcomes traditional design limits, enabling diverse metallo-supramolecular architectures with advanced functions.

## Graphical abstract

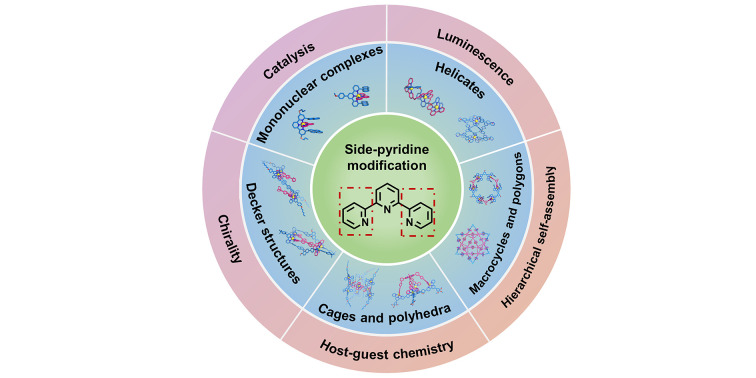

## 1 Introduction

Self-assembly is a fundamental organizational principle observed throughout nature, where complex and functional structures emerge from the spontaneous and ordered association of simple components through non-covalent interactions.^1–3^ In biological systems, for example, this process underpins the formation of essential macromolecular architectures—from the double helix of DNA (guided by hydrogen bonding) to the phospholipid bilayers of cell membranes (driven by hydrophobic effects). Inspired by these natural paradigms, chemists have developed supramolecular self-assembly to construct artificial supramolecular systems with tailored properties. Among the diverse array of non-covalent interactions, coordination interactions play a pivotal role. Metal–ligand bonds (15–50 kcal mol^−1^) exhibit intermediate strength between covalent bonds (60–120 kcal mol^−1^) and weak dispersive interactions (0.5–10 kcal mol^−1^), enabling self-correction and thermodynamic control during the assembly process.^4^ Additionally, the well-defined coordination geometries of metal ions and rigid ligands allow predictable structural outcomes. By employing coordination, numerous discrete 2D/3D architectures, such as metallacycles,^5–7^ metallacages,^8–10^ helicates,^11–13^ catenanes,^14–16^ rotaxanes,^17–19^ and knots,^20–22^ were predictably designed. The synthetic self-assembly offers programmable control over symmetry, topology, and functionality through rational ligand design and metal ion selection.

Among a wide variety of organic coordination motifs, 2,2′:6′,2′′-terpyridine (tpy) as a tridentate chelating motif has emerged as a key component in constructing coordination-driven supramolecular architectures, owing to its unique combination of structural directionality, electronic tunability, and coordination stability. As a classical tridentate N-donor motif, tpy forms stable 〈tpy-M^2+^-tpy〉 complexes with various transition metal ions, such as Zn^2+^, Cd^2+^, Mn^2+^, Cu^2+^, Co^2+^, Fe^2+^, Ru^2+^, and Os^2+^, characterized by nearly linear coordination geometry (≈180° bond angle). These characteristics provide a high level of predictability for supramolecular design, which allows for the rational design of discrete supramolecular architectures with high precision in spatial arrangement and dimensional control. Beyond structural precision, metal-tpy coordination imparts a rich spectrum of functional attributes to the resulting assemblies, including photophysical properties^23–25^ (*e.g.*, metal-to-ligand charge transfer, tunable fluorescence/phosphorescence), redox activity,^26–28^ catalytic capability,^29–31^ and dynamic responsiveness to external stimuli.^32,33^ Moreover, the modularity of tpy-based ligands facilitates the construction of structurally diverse architectures—from linear chains and 2D grids to 3D cages and frameworks—with well-defined structures and cavities.^34–37^ Based on the advantages mentioned above, tpy-based coordination chemistry has become an essential platform for developing multifunctional systems in fields spanning chirality,^38–42^ sensing,^43–45^ catalysis,^46–50^ and emissive materials.^51–58^

From the perspective of synthesizing tpy-based coordination supramolecules, tpy typically serves as a linking unit through its coordination with metal ions. The design strategy for such ligands primarily focuses on the modification of the 4′-position of tpy with different directing units, where the number and angular arrangement of these modification sites determine the ligand's geometric characteristics and the ultimate structure of the assembled supramolecules. The functionalization of the obtained supramolecule primarily arises from the cavities formed within the structure and the intrinsic properties of the directing moieties.

**Fig. 1:**
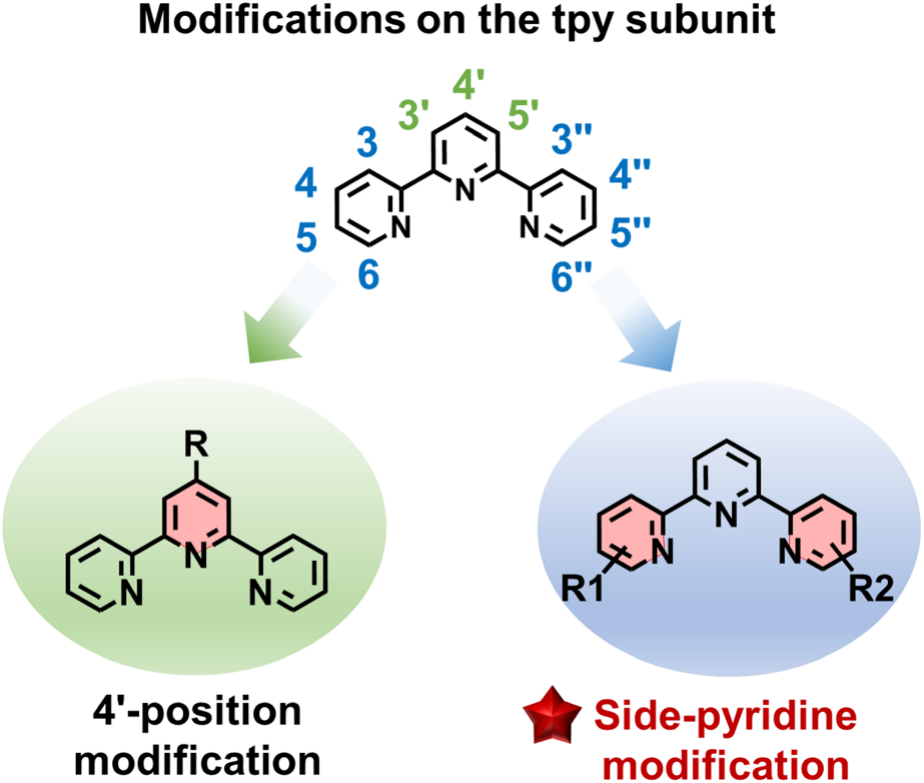
The illustration of 4′-position and side-pyridine modifications of tpy.

However, the conventional modification strategy—primarily focused on functionalizing the 4′-position of tpy—places inherent limitations on both structural construction and functional diversification. These limitations include: (1) poor coordination selectivity (ligand and metal-ion selectivity); (2) restricted substitution positions (only 4′-position) that impede rational introduction of functional units and property optimization; and (3) the intrinsic difficulty in constructing low-symmetry structures with highly symmetric ligands based on symmetric 4′-position-modified tpy moieties, where such low-symmetry structures are crucial due to their unique and irregular frameworks/cavities^59–62^ that enable either selective guest recognition^63–65^ or chiral assembly.^66–68^ To address these challenges, chemists have begun focusing on side-pyridine modification strategies of tpy to construct novel coordination supramolecular systems (Fig. 1). Compared with traditional 4′-position functionalization, side-pyridine modification offers the following advantages: (1) multiple modifiable sites (3,4,5,6,3′′,4′′,5′′,6′′-position) enable facile incorporation of functional moieties; (2) angular diversity enriches ligand geometry; (3) one-side-pyridine functionalization allows for dissymmetrical ligand design, facilitating the assembly of low-symmetry and chiral architectures; (4) the introduced substitutes around metal center affect coordination modes and selectivity. The integration of these two strategies, combining their respective advantages and compensating for their limitations, can overcome the constraints of traditional design approaches, significantly enriching the structural diversity and functional scope of tpy-based coordination chemistry. Previous excellent reviews have primarily focused on coordination supramolecular systems constructed through traditional 4′-position functionalization.^69–77^ In this review, we summarized recent advances in metallo-supramolecular architectures based on side-pyridine modification strategies, with particular discussion on novel structures and properties that are difficult to achieve through conventional approaches.

## 2 The main synthetic strategies for tpy

**Fig. 2:**
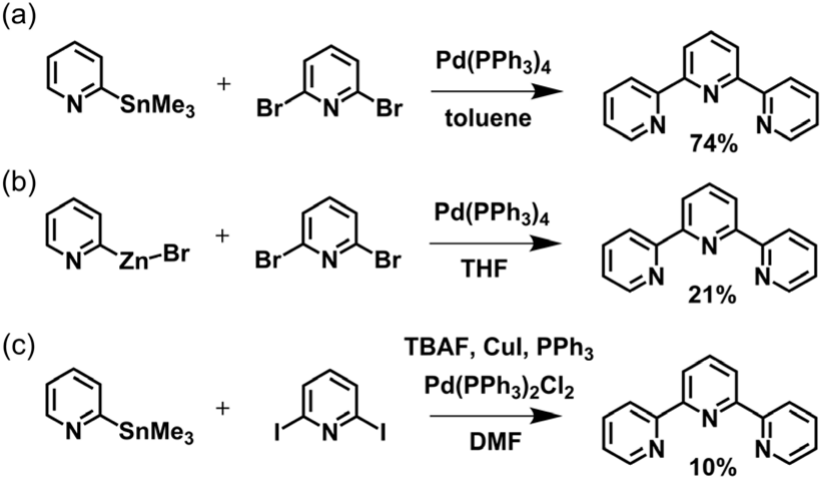
(a) Stille, (b) Negishi, and (c) Hiyama cross-coupling reaction for the synthesis of tpy.

In 1932, Gilbert Morgan first reported the synthesis of terpyridine through oxidative dehydrogenation of pyridine with FeCl_3_.^78^ However, this method produced numerous mixtures of oligopyridines and tpy regioisomers. Despite its low efficiency, this approach brought tpy and the synthetic strategy of direct synthesis *via* pyridine coupling reactions into the spotlight. Subsequently, a series of synthetic methods utilizing coupling reactions was reported. With the development of transition metal complex catalysts, the synthetic methods for tpy advanced further, exemplified by Stille,^79^ Negishi,^80^ and Hiyama^81^ cross-coupling reactions (Fig. 2). The main challenges of the coupling strategy lie in the low reactivity, sensitive substrates, limited functional group tolerance, and the high toxicity of reactants (such as toxic tin-based complexes).

In addition to coupling reactions, alternative strategies for constructing tpy skeletons, specifically through central pyridine or side pyridine ring formation, have been developed, offering promising solutions to the challenges mentioned above. This approach also provides a feasible route for synthesizing side-substituted tpy. For instance, halogen-substituted tpy, which maintains active sites in cross-coupling reactions, is difficult to prepare *via* cross-coupling methods.

**Fig. 3:**
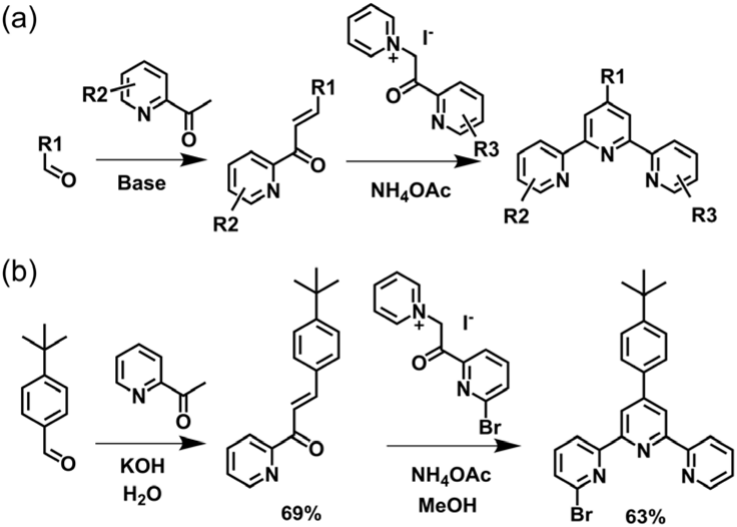
(a) Kröhnke methodology for the synthesis of 4′-aryl-tpy. (b) The synthetic pathway of 4′-(*tert*-butyl)-6-bromo-tpy.

Kröhnke's methodology^82^ has been widely utilized for the synthesis of 4′-aryl-tpy (Fig. 3a). The process initiates with an aldol condensation between 2-acetylpyridine and aromatic aldehydes mediated by alcoholic or aqueous KOH, leading to the formation of enone intermediate. This is followed by a Michael addition reaction with pyridinium salt obtained from 2-acetylpyridine, which produces the 1,5-diketone compound. Without isolation, subsequent *in situ* cyclization facilitated by ammonium acetate affords the desired tpy product in good yields on a gram scale. It is noteworthy that enones and pyridinium salts bearing different substituents enable the precise synthesis of dissymmetrically side-pyridine-modified tpy, as exemplified by the synthesis of 4′-(*tert*-butyl)-6-bromo-tpy reported by Wang and coworkers (Fig. 3b).^83^

**Fig. 4:**
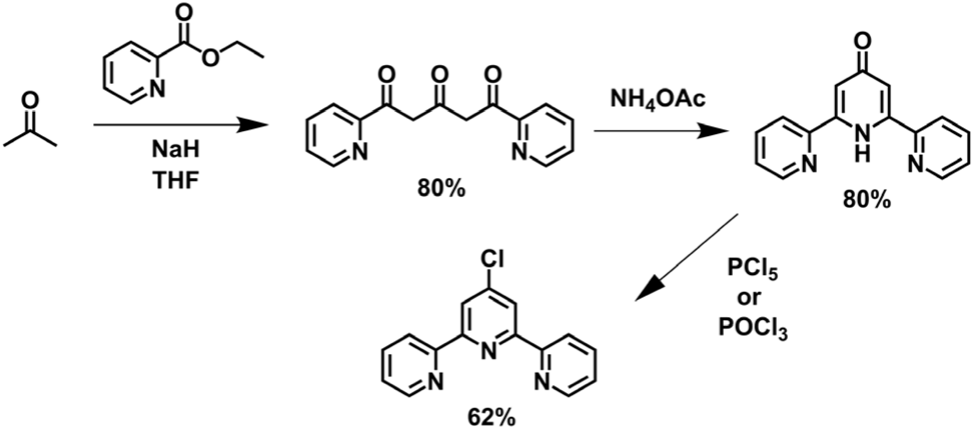
Kröhnke methodology for the synthesis of 4′-chloro-tpy.

Constable and coworkers reported the preparation of 4′-substituted terpyridine by constructing the central pyridine ring using the Kröhnke methodology (Fig. 4). The process involves the reaction of ethyl picolinate with acetone to form a triketone intermediate, followed by condensation with ammonium acetate (as a source of nitrogen) and subsequent chlorination using PCl_5_/POCl_3_ to give 4′-chloro-tpy with 62% yield at the last step.^84^

**Fig. 5:**
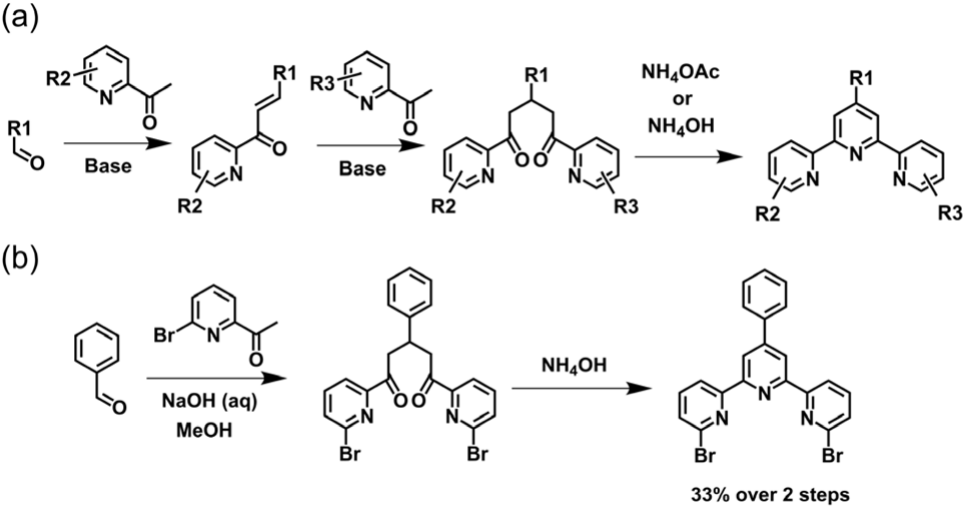
(a) Potts methodology for the synthesis of 4′-aryl-tpy *via* Knövenagel condensation. (b) The synthetic pathway of 6,6′′-dibromo-tpy.

The Knövenagel condensation provided an efficient route to 4′-aryl-substituted tpy as well. This method developed by Potts and coworkers proceeded *via* the formation of enone and diketone intermediate from 2-acetylpyridine and aryl aldehyde under mild conditions (Fig. 5a). Without isolation, this intermediate can be directly subjected to a one-pot cyclocondensation with ammonium acetate/ammonia, yielding the corresponding 4′-aryl-substituted tpy.^85^ This efficient and straightforward method has been widely adopted for the preparation of tpy-based ligands on a gram scale for the synthesis of metallo-supramolecules. It is also noteworthy that the use of substituted 2-acetylpyridines enables direct access to side-pyridine-modified tpy. For instance, Chan and co-workers employed 2-acetyl-6-bromopyridine and benzaldehyde as starting materials to synthesize 6,6′′-dibromo-tpy with a yield of 33% (Fig. 5b).^86^ Notably, this strategy can also be adapted for the synthesis of dissymmetrically side-pyridine-modified tpy. The key intermediate, the enone, can first be isolated and subsequently react with a differently substituted 2-acetylpyridine derivative to construct the dissymmetrical tpy.

**Fig. 6:**
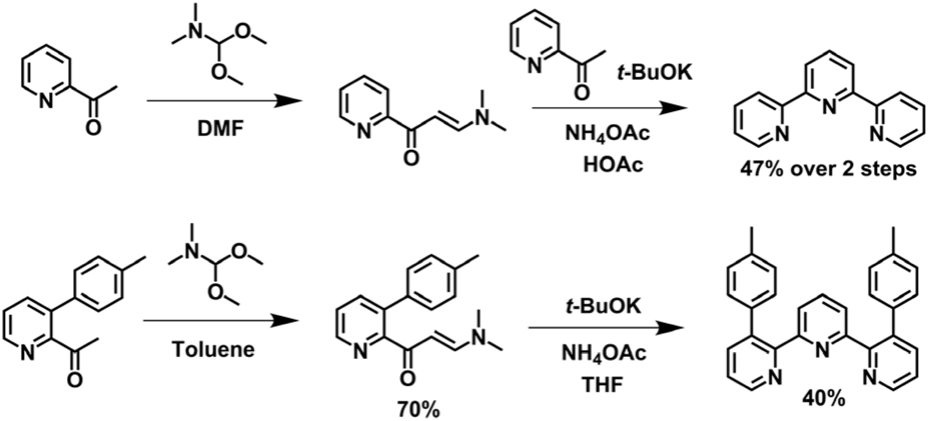
Jameson's modified method for the synthesis of tpy and 3,3′′-diaryl-tpy.

Based on the Potts methodology, Jameson and coworkers developed a synthetic approach for constructing tpy bearing non-aryl substituents at the 4′-position (Fig. 6). Utilizing 2-acetylpyridine and *N*,*N*-dimethylformamide-dimethylacetal to form an enaminone, which was then reacted with the potassium enolate of 2-acetylpyridine, followed by a Michael cyclization mediated by ammonium acetate, afforded tpy in 47% yield. The introduction of pre-substitutions on 2-acetylpyridine enables the synthesis of side-modified tpy, as exemplified by the preparation of 3,3′′-diaryl-tpy.^87,88^

**Fig. 7:**
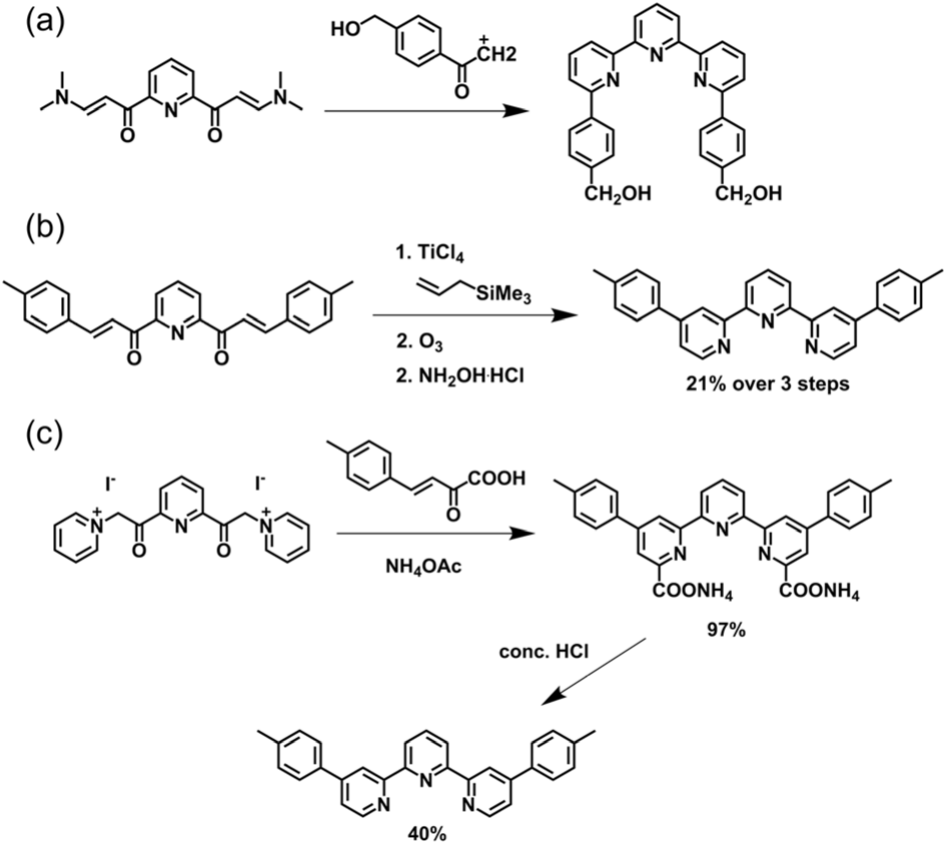
The synthesis of tpy *via* (a) Jameson's method, (b) Hosomi–Sakurai addition, and (c) Kröhnke heterocyclization for the synthesis of tpy.

In addition to the construction of the central pyridine ring, cyclization strategies analogous to those described above can also be applied to the formation of the side pyridine of tpy. Classic examples include the Jameson method,^88^ Hosomi–Sakurai addition,^89^ and Kröhnke heterocyclization,^82^*etc.* (Fig. 7). However, since both side pyridine rings are formed in a single reaction step, this strategy poses significant challenges for the precise synthesis of dissymmetrically modified tpy.^90^

## 3 Self-assembly of metallo-supramolecular architectures

### 3.1 Mononuclear complexes

Mononuclear complexes are the smallest structures formed by the coordination of organic ligands with metal ions. Due to the presence of three coordinating nitrogen atoms in tpy, most transition metal ions can accommodate two tpy units to form M(tpy)_2_-type complexes. Compared with polynuclear complexes, mononuclear complexes feature simpler synthesis and easier structural characterization, making them ideal model compounds for studying the self-assembly behavior between ligands and metals as well as the influence of substituents on the physicochemical properties of complexes.

In solution, tpy-based ligands and transition metal ions typically self-assemble to form M(tpy)_2_-type complexes. However, when two different tpy-based ligands (A and B) are introduced into the system, the low ligand selectivity often leads to the formation of a statistical mixture of M(A)_2_, M(B)_2_, and M(AB) complexes. Although stepwise synthesis can achieve precise preparation of heteroleptic complexes by multi-step synthesis and separation, it is often time-consuming, low-yielding, and unsuitable for constructing structurally diverse or dynamically responsive architectures. The one-pot self-assembly approach for selectively synthesizing heteroleptic species remains challenging. This requires rational ligand design to introduce additional stabilizing interactions (*e.g.*, attractive forces between different ligands) to favor the heteroleptic complex or repulsive forces between identical ligands to suppress homoleptic complex formation.

**Fig. 8:**
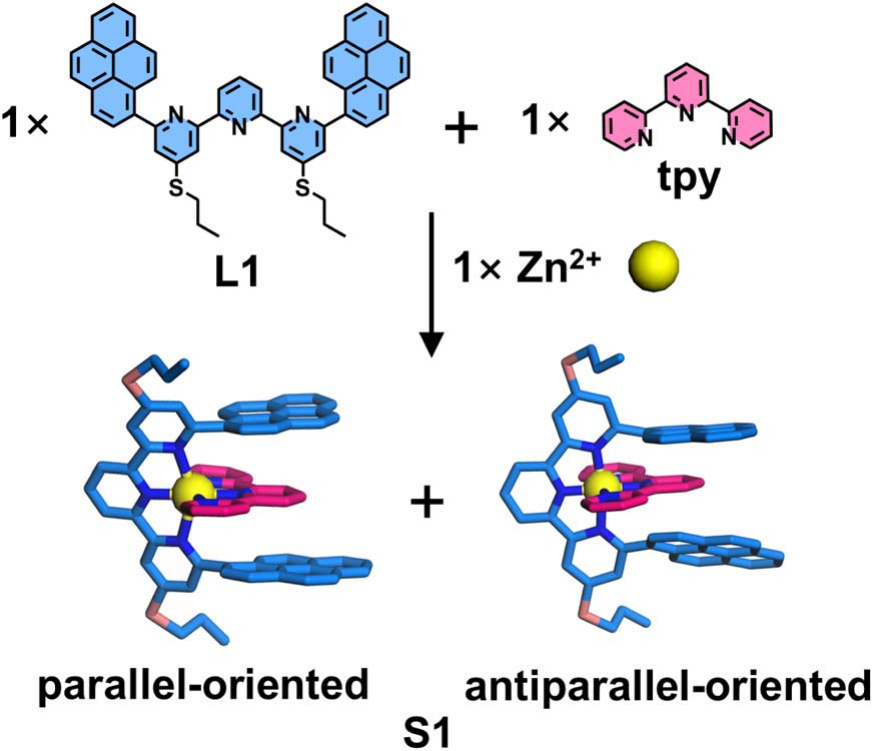
Structures of heteroleptic complex S1 exhibiting parallel (crystal structure) and antiparallel (simulated structure) configurations assembled from ligands L1 and tpy.

Lehn and coworkers pioneered the selective synthesis of heteroleptic complex S1 by introducing a pyrene chromophore at the 6,6′′-position of tpy (Fig. 8).^91^ Due to the two dissymmetrical conformations of the pyrene substituent, the self-assembly of ligands L1, tpy, and Zn^2+^ can generate a pair of isomers exhibiting parallel and antiparallel configurations, as confirmed by low-temperature nuclear magnetic resonance (NMR) spectroscopy. Single-crystal X-ray diffraction (SCXRD) revealed significant overlap between the pyrene chromophore and the tpy unit, forming a π–donor–π–acceptor–π–donor triad with an average centroid–centroid distance of 3.5 Å.

**Fig. 9:**
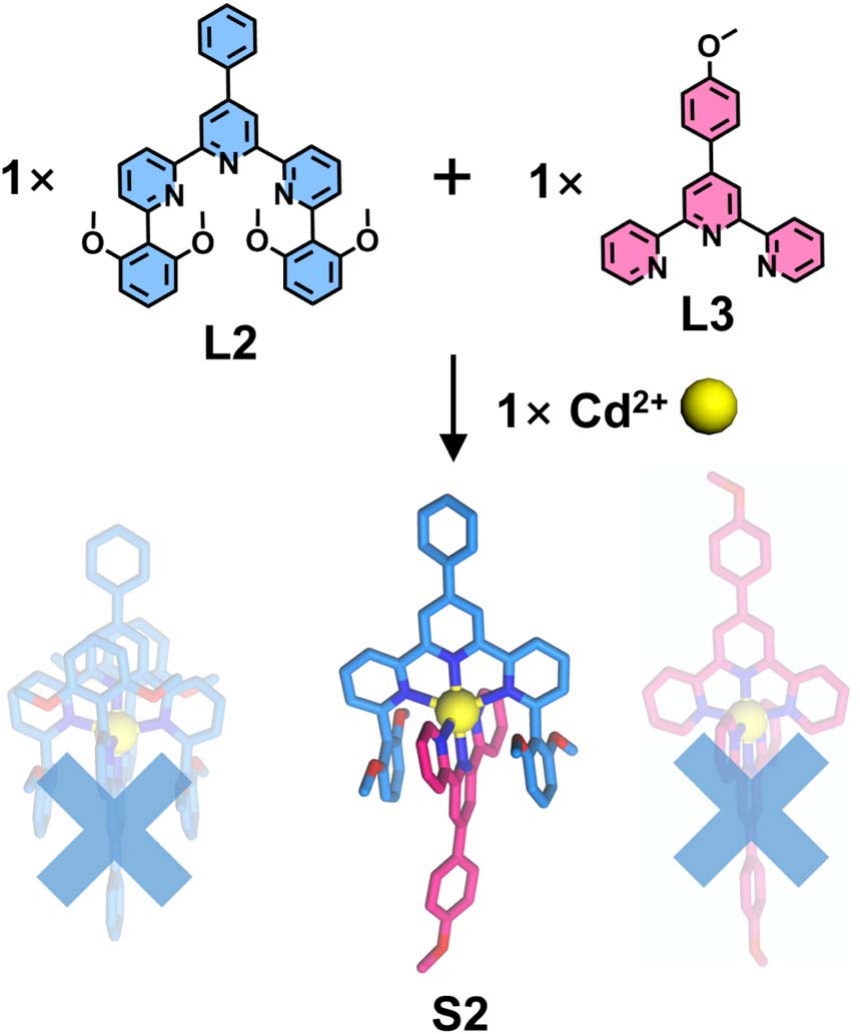
Crystal structure of heteroleptic complex S2 assembled from ligands L2 and L3.

Motivated by this pioneering study, Chan and coworkers introduced 2,6-dimethoxyphenyl substituents at the 6,6′′-position of tpy, establishing pre-designed complementary complexation between two tpy-based ligands (L2 and L3). Upon co-assembly of L2 with an unmodified ligand L3 and Cd^2+^ ions (Fig. 9), NMR and electrospray ionization mass spectrometry (ESI-MS) characterization confirmed the exclusive formation of the heteroleptic complex S2, with no observable homoleptic species, demonstrating the efficacy of this strategy in enhancing ligand selectivity (100%). SCXRD further revealed that the 6,6′′-position substituents were in close proximity to both the metal center and the adjacent ligand L3, providing ancillary ion–dipole interactions and additional π-stacking stabilization in the resulting heteroleptic complex S2, thereby improving coordination selectivity.^86^

**Fig. 10:**
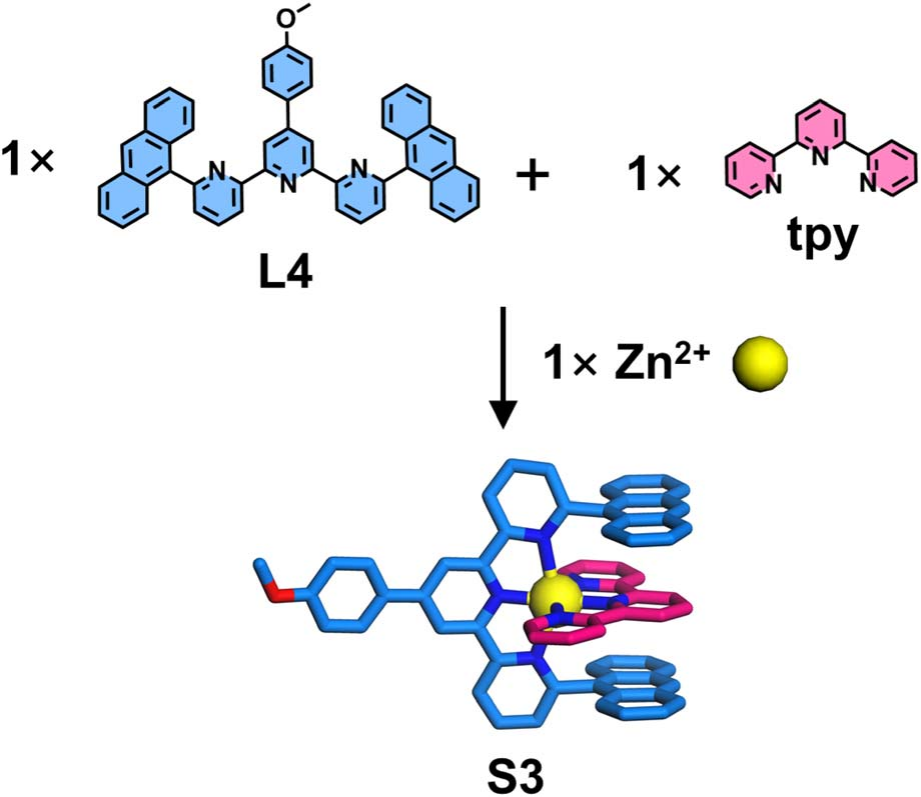
Crystal structure of heteroleptic complex S3 assembled from ligands L4 and tpy.

Besides 2,6-dimethoxyphenyl groups, anthracene-based substituents were also shown to stabilize the heteroleptic architecture. Chan and coworkers introduced a sterically demanding anthracene chromophore at the 6,6′′-position of tpy to construct the heteroleptic complex S3 (Fig. 10). Employing the same strategy, the anthracene moiety effectively suppressed the formation of the homoleptic L4 complex while promoting π-stacking with the tpy unit, thereby achieving highly selective coordination (100% selectivity).^92^

**Fig. 11:**
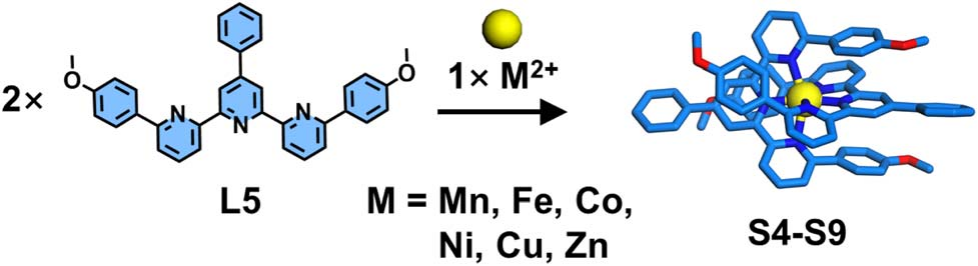
Crystal structure of complexes S4–S9 assembled from ligand L5.

The steric hindrance imposed by bulky substituents at the 6,6′′-position can significantly retard the formation rate of homoleptic complexes. When smaller steric groups are employed, homoleptic products may still form competitively. Furthermore, repulsive interactions between steric groups can also influence the coordination geometry of the resulting complexes. Wang, He, Liu, and coworkers reported the synthesis of mononuclear homoleptic complexes featuring helical conformations (Fig. 11). The authors designed ligand L5 by introducing 4-dimethoxyphenyl groups at the 6,6′′-position of tpy, which subsequently self-assembled with various metal ions to form complexes S4–S9. SCXRD analysis revealed that the steric hindrance from these substituents significantly altered both the metal–ligand bond lengths and coordination angles, thereby inducing the formation of helical conformations in the resulting complexes.^93^

**Fig. 12:**
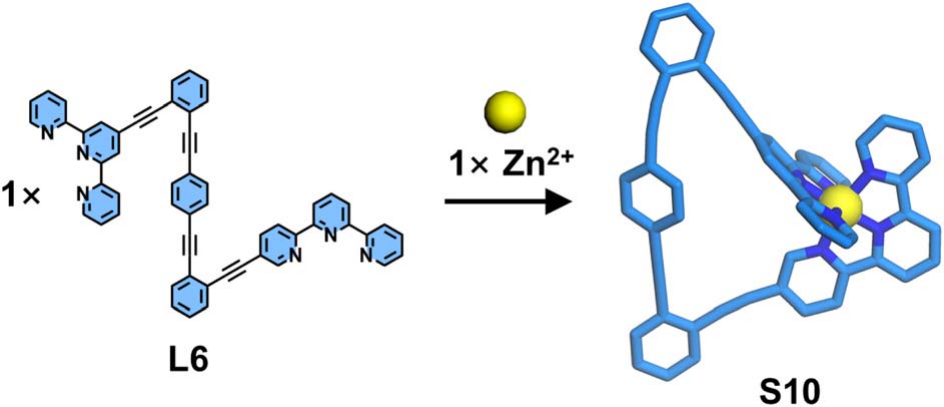
Simulated structure of complex S10 assembled from ligand L6.

The above examples demonstrate that substituents at the 6,6′′-positions can interact with another ligand, thereby influencing coordination behavior. However, each substitution position possesses characteristic geometric angles that can be strategically exploited to construct specialized architectures. Schmittel and coworkers reported an example of a mononuclear molecular triangle S10, constructed from a ditopic dissymmetrical ligand L6 containing a 4′-position-modified tpy and a 5-position-modified tpy (Fig. 12). Benefiting from the angular distortion of the 5-position-modified tpy, the coordination geometry at the metal center deviates from linearity upon binding to the 4′-position tpy, adopting an angle close to 120°. This configuration serves as a triangular vertex, facilitating the self-assembly of ligand L6 with metal ions to form a mononuclear complex.^94,95^

### 3.2 Helicates

The term “helicate” was first introduced by Lehn and coworkers in 1987 to describe a polymetallic helical complex that contains one or more ligand strands and two or more metal centers.^96^ To construct helicates, the choice of coordinating units is crucial. Typically, bidentate chelating coordinating units with tetrahedral or octahedral coordination geometries and tridentate chelating ligands with octahedral or tricapped trigonal prismatic geometries serve as ideal building blocks, as their coordination centers inherently introduce the necessary conditions for helix formation.^97,98^ Additionally, the spacers linking these coordinating units play a decisive role in determining ligand flexibility. These spacers must prevent self-coordination between coordinating units within a single strand while maintaining sufficient flexibility to facilitate ligand twisting and promote helicate formation. In this context, the tpy unit, with its octahedral coordination geometry, serves as a suitable building block for helicate assembly. However, conventional 4′-position modifications fail to provide the optimal angular orientation required for helix formation, whereas 5-position modifications leverage their inherent angular distortion to facilitate helicate construction.

**Fig. 13:**
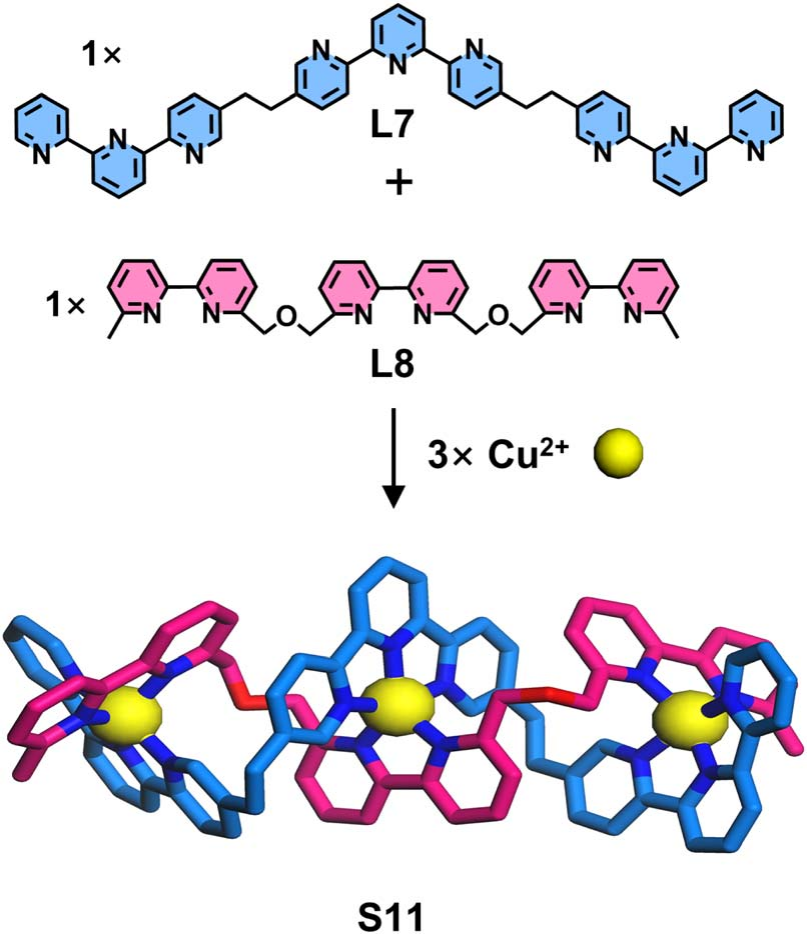
Simulated structure of helicate S11 assembled from ligands L7 and L8.

**Fig. 14:**
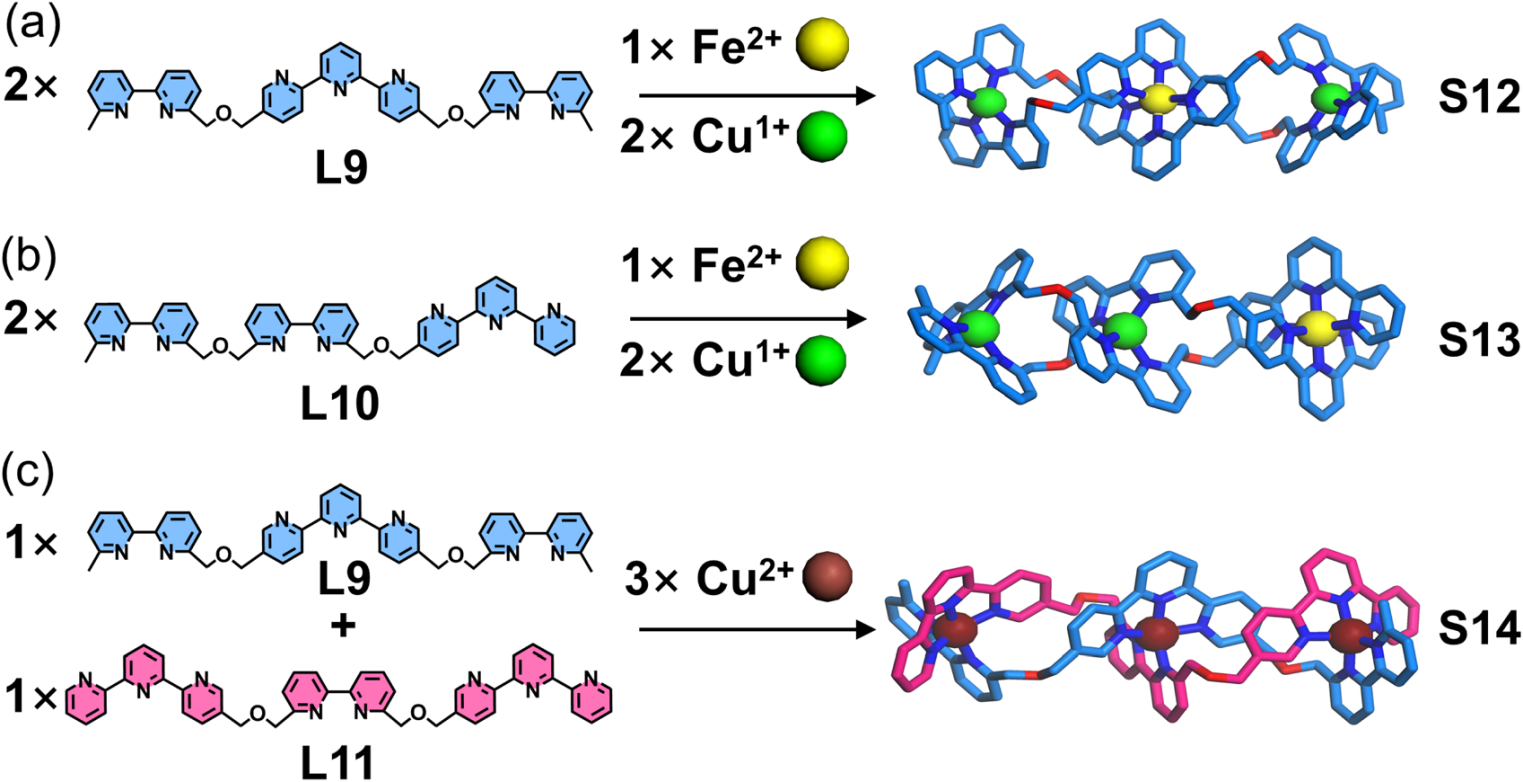
(a) Simulated structure of helicate S12 assembled from ligand L9. (b) Simulated structure of helicate S13 assembled from ligand L10. (c) Simulated structure of helicate S14 assembled from ligands L9 and L11.

Ligand strands based on bipyridine and tpy units and their assembly into double-stranded helicates were reported by Lehn, Hasenknopf, and coworkers (Fig. 13). In this system, the tpy moiety adopts a 5-position modification strategy to achieve geometric angle requirements similar to those of bipyridine.^99^ Two types of tritopic ligand strands (L7 and L8), composed exclusively of either tpy or bipyridine coordination units, respectively, were designed and synthesized. Two coordination units can form five-coordinate heteroleptic complexes with Cu^2+^. Taking advantage of this property, L7 and L8 co-assemble with Cu^2+^ to generate hetero-duplex complex S11, whose single-crystal structure was successfully obtained. This selective coordination behavior between different ligands resembles the DNA double helix, where two single strands are held together by complementary hydrogen bonding between nucleobase pairs. However, unlike DNA strands—which contain different nucleobases (A, T, G, C) acting as distinct recognition units—these ligand strands contain only a single type of coordination unit within each strand. Subsequently, Lehn and coworkers reported the design and synthesis of double-stranded helicates with enhanced DNA-like characteristics (Fig. 14). The authors achieved a closer structural mimicry of DNA single strand by incorporating both bipyridine and tpy coordination units into each ligand strand. Three tritopic ligands (L9–L11) with distinct sequences of these units were synthesized. The system exploits the differential metal-coordination preferences of the units: the bipyridine unit forms a tetrahedral coordination geometry with Cu^+^, while tpy adopts an octahedral configuration with Fe^2+^. Notably, bipyridine and tpy can cooperatively bind to five-coordinate Cu^2+^. This metal–ligand recognition pattern bears striking similarity to the complementary hydrogen bonding between nucleobase pairs in DNA double helices. When ligand strands (either L9 or L10 exclusively) were assembled with Cu^+^ or Fe^2+^, they formed homo-duplex complexes S12 and S13, respectively. In contrast, the combination of two different ligand strands (L9 and L11) with Cu^2+^ resulted in the formation of a hetero-duplex complex S14.^100^

**Fig. 15:**
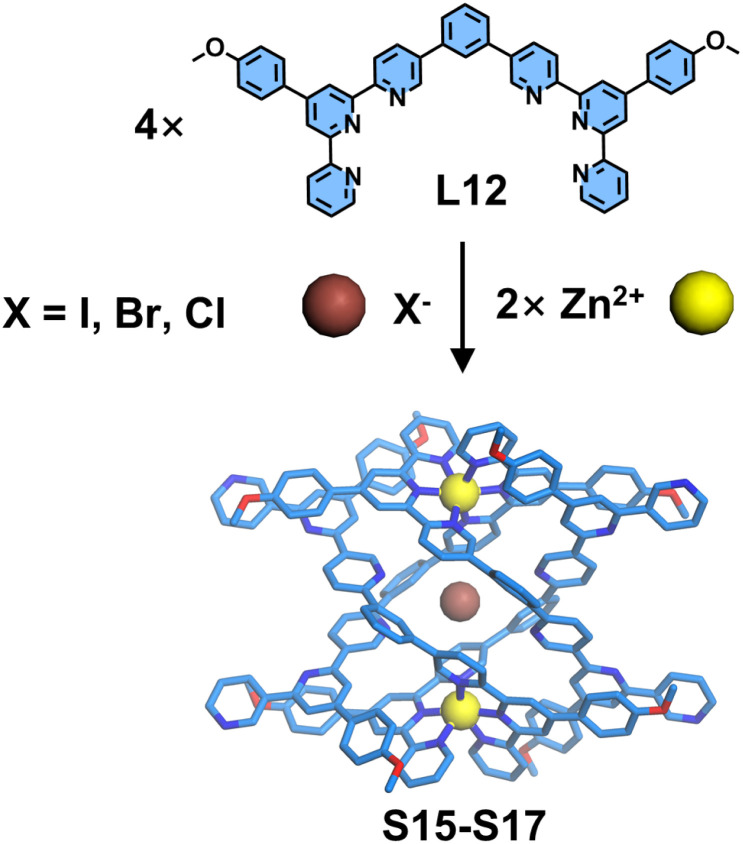
Crystal structures of helicates S15–S17 assembled from ligand L12 with halide ion templating.

The flexible spacers (*e.g.*, alkyl chains or polyether linkers) between coordination units enable ligand strands to adopt the necessary conformational twisting for helicate formation, whereas rigid spacers typically impede this process. Notably, Wu, Wang, and coworkers developed a novel ditopic ligand L12 incorporating a rigid benzene spacer, where the tpy units were strategically functionalized at the 5-position (Fig. 15). When ligand L12 was assembled with Zn^2+^, it initially formed a series of macrocyclic structures (di-, tri-, and tetranuclear cycles). Intriguingly, in the presence of halide anions (l^−^, Br^−^, and Cl^−^) as structure-directing templates, these macrocycles underwent topological transformation into quadruple-stranded helicates S15–S17. In this reconfigured architecture, one tpy unit from each ditopic ligand remained uncoordinated to Zn^2+^. The helicate stabilization arose from a synergistic combination of C–H⋯X hydrogen bonds (X = halide), anion–π interactions, π–π stacking, and electrostatic contacts. The authors systematically investigated halide binding affinities *via* NMR and gradient tandem mass spectrometry (gMS^2^), revealing the trend: Cl > Br > I in thermodynamic stability. Paradoxically, iodide—despite exhibiting the weakest binding—demonstrated the highest structural selectivity during helicate formation, contradicting conventional stability–selectivity correlations. Based on these experimental observations, mechanistic rationale for this anomalous phenomenon was proposed, elucidating the helicate formation pathway as follows: the nucleophilic character of halide anions initially disrupts the coordination bonds to generate a dimeric intermediate state, which subsequently evolves into the helicate structure through multivalent noncovalent interactions. Notably, iodide—exhibiting the strongest nucleophilicity among halides—preferentially stabilizes the transition state, thereby demonstrating the highest selectivity despite its weakest thermodynamic binding affinity.^67^

**Fig. 16:**
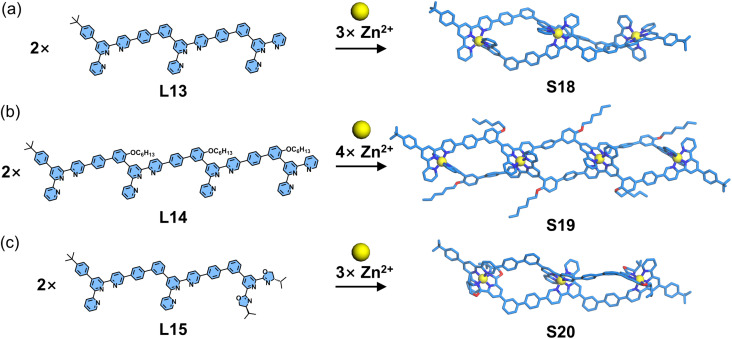
(a) Crystal structure of helicate S18 assembled from ligand L13. (b) Simulated structure of helicate S19 assembled from ligand L14. (c) Simulated structure of helicate S20 assembled from ligand L15.

An alternative rigid–ligand-based helicate system was reported by Wang, Ma, and coworkers (Fig. 16). Employing the same 5-position functionalization strategy, the authors designed and synthesized a series of dissymmetrical ladder-type multitopic ligands (L13–L15). Upon assembly with Zn^2+^, these ligands adopted a head-to-tail coordination mode to form double-stranded helicates (S18–S20), respectively. Key structural features were confirmed by SCXRD of S18, which revealed the chiral conformation of the helicate architecture. Notably, the chiral L15 incorporated a 2,6-bis(oxazolinyl)pyridine chelating group at its tails, which effectively induced pronounced circular dichroism (CD) signals in the resulting helicate.^101^

### 3.3 2D macrocycles and polygons

The 〈tpy-M-tpy〉 motif adopts a linear coordination geometry. Combined with the conventional 4′-position functionalization strategy, this feature renders tpy units advantageous for constructing 2D coordination supramolecular architectures, including macrocycles and polygons.^102–105^ In these structures, the 〈tpy-M-tpy〉 units typically serve as edges of the polygons, while angular directing groups—often organic molecules with two substitutable sites—act as vertices. Guided by this strategy, numerous 2D architectures based on tpy have been extensively reported. Recently, by integrating this conventional macrocycle construction approach with a side-pyridine modification strategy, a series of novel coordination macrocyclic systems have emerged, further enriching the family of 2D tpy-based coordination architectures.

**Fig. 17:**
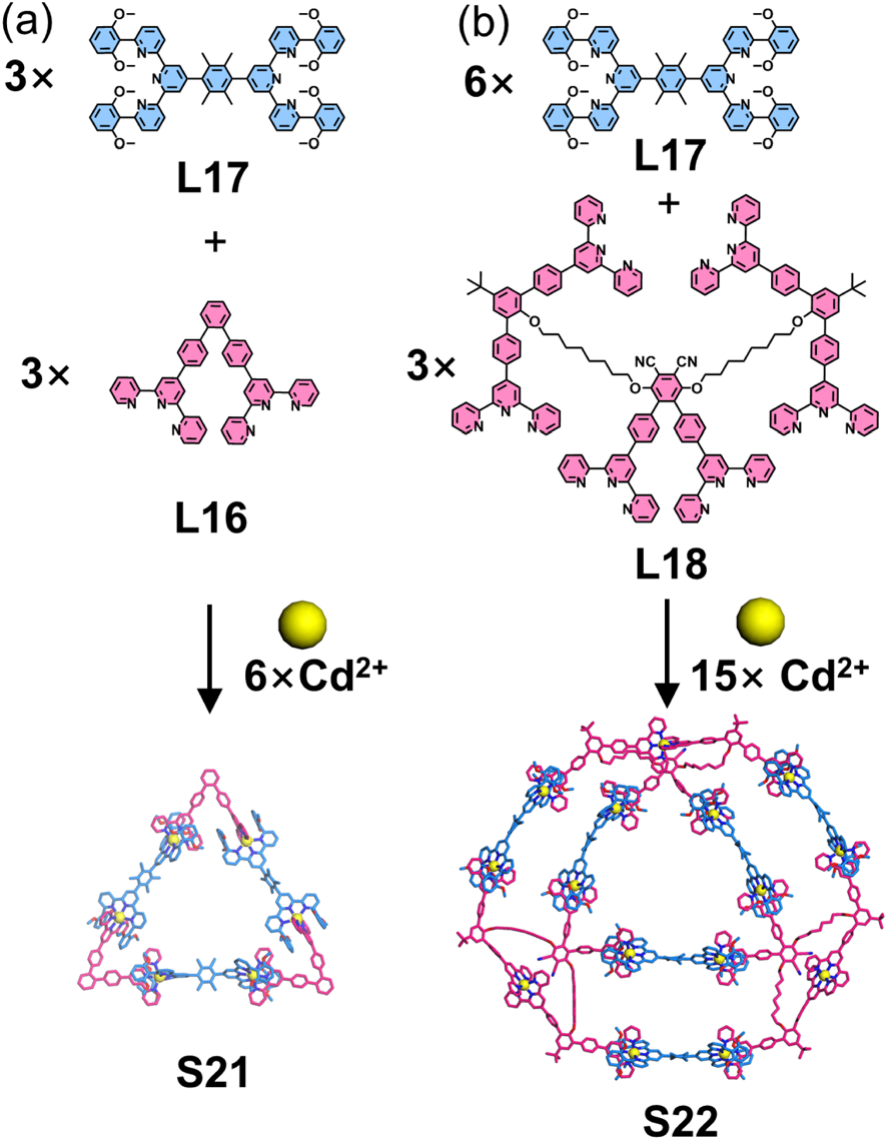
(a) Simulated structure of triangle S21 assembled from ligands L16 and L17. (b) Simulated structure of ditrigon S22 assembled from ligands L18 and L17.

Chan and coworkers developed a complementary ligand pair with self-recognition properties based on the 6,6′′-position functionalization strategy of tpy, as introduced in Section 3.1. Building upon this work, the team subsequently reported the self-assembly of a series of heteroleptic 2D architectures. The assembly of 60°-directed ditopic ligands (L16), 180°-directed ditopic ligands (L17) featuring four 2,6-dimethoxyphenyl substituents, and Cd^2+^ ions yielded the heteroleptic coordination triangle S21, which was unambiguously confirmed by SCXRD (Fig. 17). Notably, NMR and ESI-MS confirmed the exclusive formation of heteroleptic triangle S21, with no observable homoleptic triangle assembled by L16 and Cd^2+^ ions only, demonstrating the exceptional precision and efficiency of the assembly process. Capitalizing on these remarkable results, the authors designed and synthesized a hexatopic ligand (L18), comprising three ditopic ligands connected by two alkyl chain spacers, to construct ditrigon S22. Comprehensive characterization by NMR and ESI-MS confirmed the formation of the ditrigon, while transmission electron microscopy (TEM) and atomic force microscopy (AFM) imaging directly visualized individual double-triangle molecules, with measured dimensions in excellent agreement with computational models.^86^

**Fig. 18:**
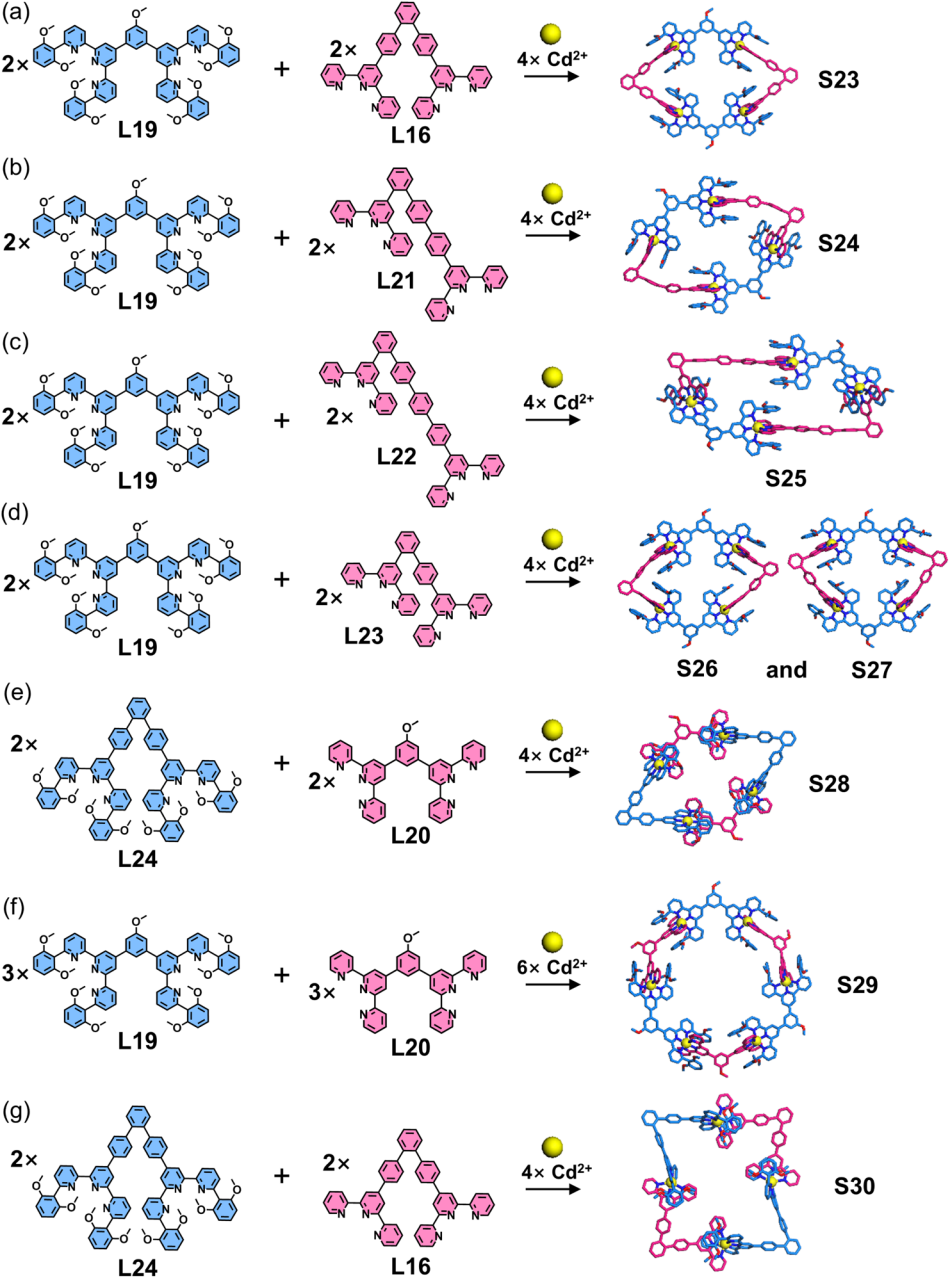
(a) Simulated structure of polygon S23 assembled from ligands L16 and L19. (b) Simulated structure of polygon S24 assembled from ligands L19 and L21. (c) Crystal structure of polygon S25 assembled from ligands L19 and L22. (d) Simulated structures of polygons S26 and S27 assembled from ligands L19 and L23. (e) Simulated structure of polygon S28 assembled from ligands L20 and L24. (f) Simulated structure of polygon S29 assembled from ligands L19 and L20. (g) Crystal structure of polygon S30 assembled from ligands L16 and L24.

Building upon a similar strategy, the same group constructed a series of additional polygonal architectures by systematically varying the angular orientation of directing groups and the arm length of ligands. 60°-directed (L16 and L21–L24) and 120°-directed ditopic ligands (L19 and L20) were designed and synthesized. Guided by the geometric requirements of polygons, the co-assembly of 60°-directed and 120°-directed ligands with Cd^2+^ ions yielded polygon structures S23–S28 (Fig. 18), while the combination of two 120°-directed ligands produced hexagonal architecture S29. Notably, L21–L23 represent dissymmetrical ditopic ligands with unequal arm lengths. Interestingly, when the dissymmetrical ligand L23 with slight arm-length difference was assembled with L19 and Cd^2+^ ions, isomeric macrocycle mixture (S26 and S27) formed. It is well-established that three 60°-directed ligands typically generate triangular structures, while the co-assembly of two distinct 60°-directed ligands (L16 and L24) with Cd^2+^ ions resulted in a cyclic tetramer S30.^106^

**Fig. 19:**
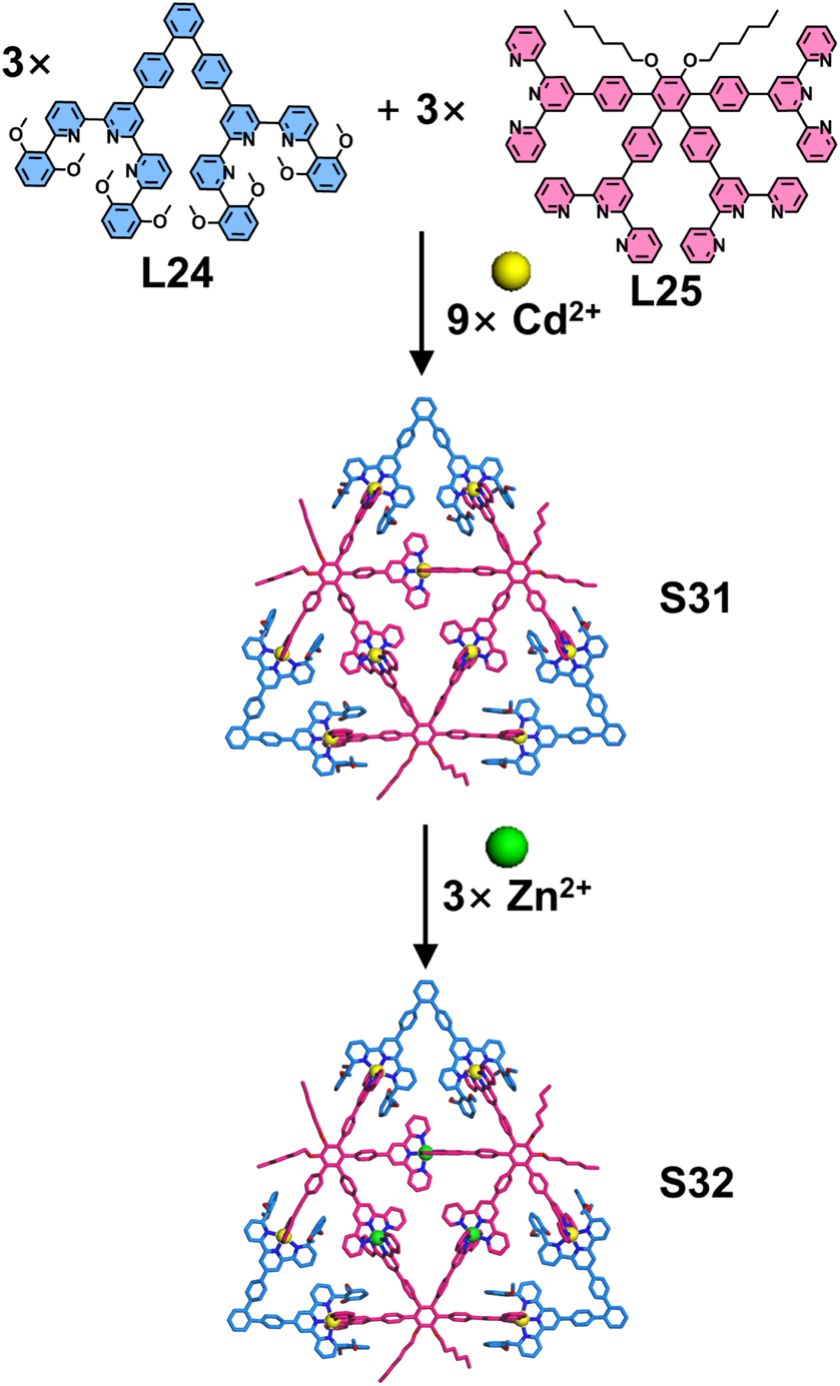
Simulated structure of Sierpiński triangle S31 assembled from ligands L24 and L25 and heterometallic Sierpiński triangle S32 from metal exchange.

The stepwise assembly strategy is commonly employed to construct highly intricate architectures containing multitopic tpy-based ligands.^107–110^ This approach typically involves: (1) nonlabile metal coordination (*e.g.*, Ru^2+^, Fe^2+^) to pre-organize ligands into stable metallo-ligands, which are isolated *via* column chromatography, followed by (2) labile metal coordination (*e.g.*, Zn^2+^, Cd^2+^) to assemble the final architectures. This strategy was developed to circumvent the common problem of low coordination selectivity in multitopic ligands, which often leads to mismatched assemblies and failure to form the desired architectures. In contrast, the complementary ligand pair offers significantly enhanced coordination selectivity, enabling a one-pot synthesis of intricate 2D architectures. Chan and coworkers reported the precise construction of a heteroleptic Sierpiński triangle (S31) through the coordination-driven assembly of a K-shaped ligand (L25), a 60°-directed ligand (L24), and Cd^2+^ ions (Fig. 19). Remarkably, upon treatment with Zn^2+^ at 50 °C for 2 hours, S31 underwent complete metal exchange at its inner Cd^2+^ sites while maintaining its structural integrity, yielding a well-defined heterometallic Sierpiński triangle S32.^106^

**Fig. 20:**
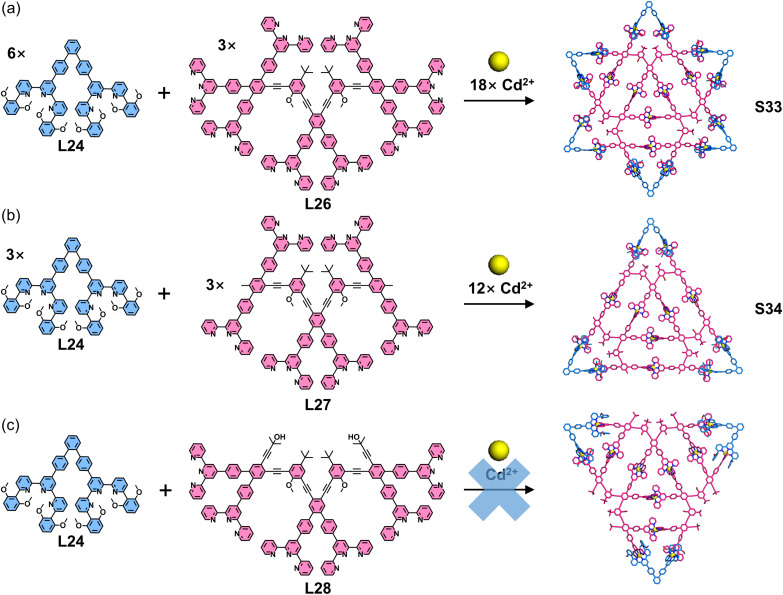
(a) Simulated structure of star hexagon S33 assembled from ligands L24 and L26. (b) Simulated structure of triangle S34 assembled from ligands L24 and L27. (c) Proposed triangle structure from self-assembly of ligands L24 and L28, which has been demonstrated to be unfeasible.

**Fig. 21:**
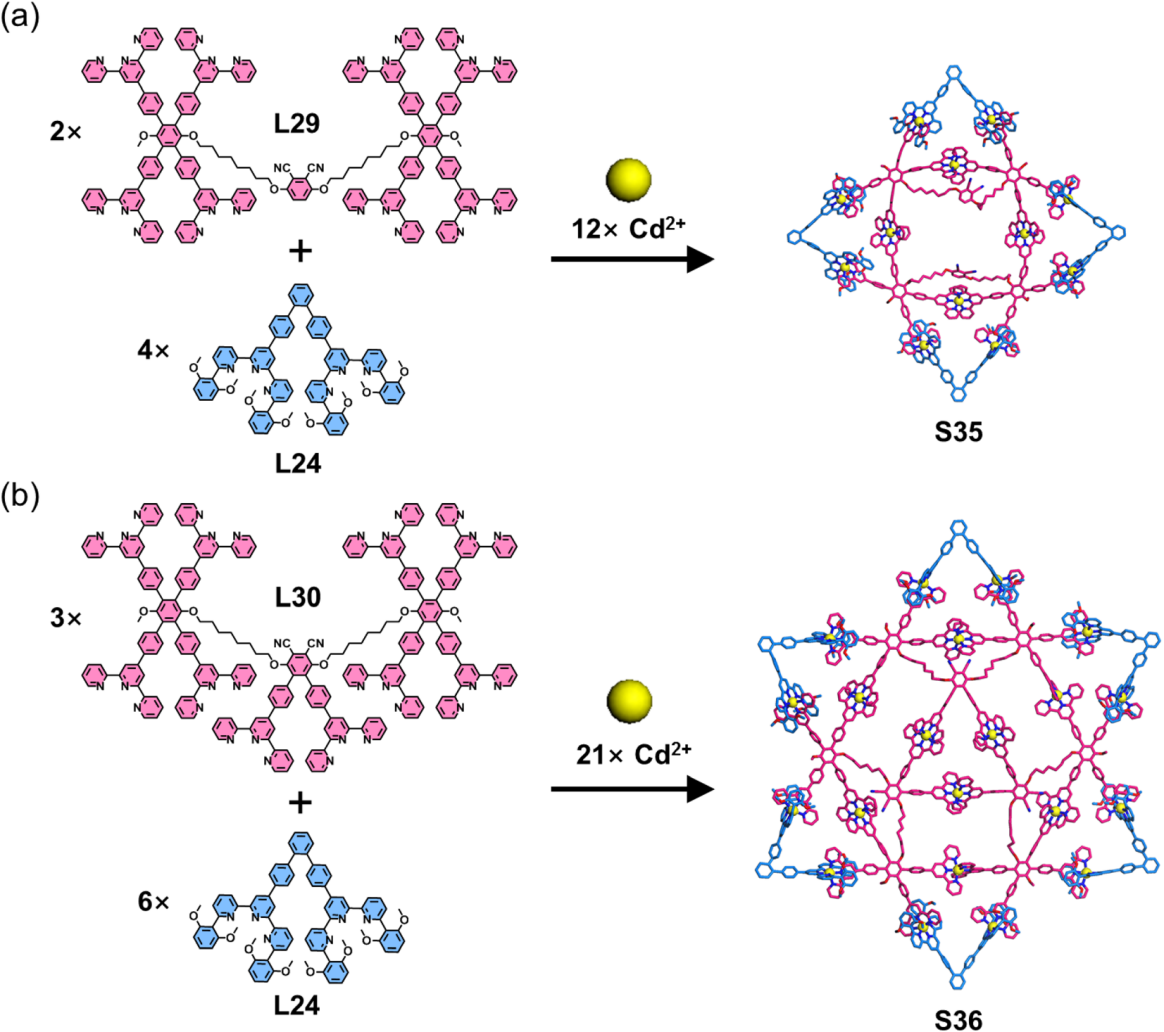
(a) Simulated structure of four-pointed star S35 assembled from ligands L24 and L29. (b) Simulated structure of six-pointed star S36 assembled from ligands L24 and L30.

Expanding this strategy combining multitopic ligands with complementary ligand pairs, the same group designed and synthesized a larger star hexagon *via* one-pot self-assembly. The assembly of a 6,6′′-substituted ditopic ligand (L24), an octatopic ligand (L26), and Cd^2+^ ions yielded the star hexagon architecture S33 (Fig. 20). Given the multiple binding sites in the octatopic ligand L26, the authors designed two hexatopic model ligands (L27 and L28, derived from L26) to investigate the assembly mechanism. Intriguingly, while the combination of L24, L27, and Cd^2+^ ions produced triangular structure S34, the analogous assembly with L28 failed to form the target triangular architecture. Mechanistic studies revealed that pre-organized building unit constructed by L27 and L24 promotes triangle formation, whereas L28 induces mismatched assembly that suppresses the desired structural outcome.^111^ Another representative example includes the self-assembly of four-pointed and six-pointed star architectures S35 and S36 (Fig. 21), achieved through the coordination of multitopic ligand L29 or L30 with 6,6′′-substituted L24 and Cd^2+^ ions, respectively.^112^

**Fig. 22:**
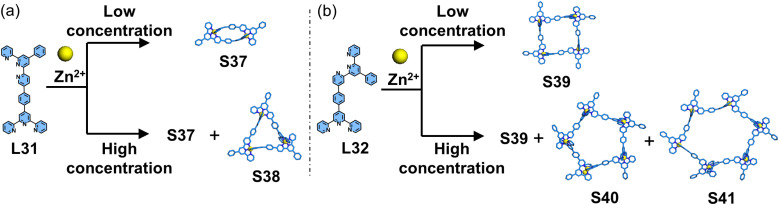
(a) Simulated structure of polygons S37 and S38 assembled from ligand L31. (b) Simulated structure of polygons S39–S41 assembled from ligand L32.

Considering that the 〈tpy-M-tpy〉 motif adopts a linear coordination geometry, conventional 4′-position modification of tpy can only position the metal centers on the edges of the resulting 2D polygons. In contrast, side-pyridine modification provides a method to adjust the directing angle, enabling the 〈tpy-M-tpy〉 center to serve as a vertex of the polygon. Wang, Shi, and coworkers reported the construction of coordination polygons using coordinated centers as directing units. By combining 5- or 4-position with 4′-position modification of tpy, dissymmetrical ditopic ligands L31 and L32 were designed and synthesized (Fig. 22). Owing to the angle modulation achieved through side pyridine modification, these two ligands assembled with Zn^2+^ ions to form discrete macrocyclic structures: at low concentrations, 60°-directed L31 and Zn^2+^ ions formed a dimer S37, while 120°-directed L32 and Zn^2+^ ions assembled into a tetramer S39. Notably, as the concentration increased, larger-sized polygon structures (S38, S40, and S41) emerged.^113^

**Fig. 23:**
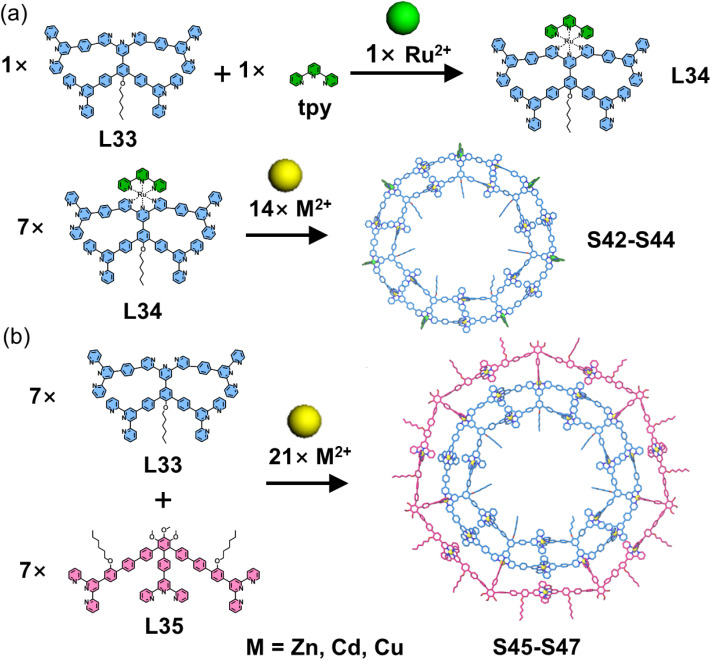
(a) Simulated structure of polygon S42–S44 assembled from ligand L34. (b) Simulated structure of polygon S45–S47 assembled from ligands L33 and L35.

Li, Zhang, Wang, and coworkers reported another example of self-assembled macrocyclic structures using tpy as the directing units (Fig. 23). The authors designed a central tpy unit with two arm-like tpy groups attached at its 4,4′′-positions. Upon coordination with Ru^2+^, the central tpy forms an inter-arm angle of 144.7°, making it highly suitable as a vertex for constructing polygonal architectures. Based on this design principle, ligand L34 was designed and synthesized. Using a stepwise self-assembly strategy, the central tpy with four bromide atoms was first coordinated with Ru^2+^, followed by Suzuki coupling to obtain the metallo-ligand L34, which was subsequently assembled with Zn^2+^, Cd^2+^, or Cu^2+^ to form double-layered heptagonal metallo-macrocycles (S42–S44). To further extend this design concept, the authors developed ligand L35 to replace the angle-fixing role of Ru^2+^, enabling one-pot synthesis of polygonal structures. Co-assembly of ligands L33, L35, and Zn^2+^, Cd^2+^, or Cu^2+^ yielded triple-layered heptagonal metallo-macrocycles (S45–S47).^114^

**Fig. 24:**
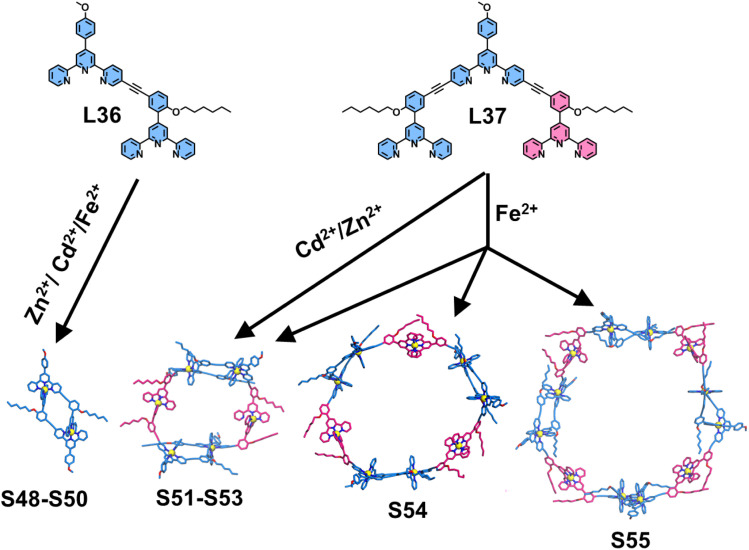
Simulated structures of dimeric motifs S48–S50 assembled from ligand L36 and polygons S51–S55 assembled from ligand L37.

In conventional tpy-based metallo-macrocycles, the 〈tpy-M-tpy〉 linkage typically serves as an edge. To enhance the structural complexity of such macrocycles, Wang and coworkers introduced novel head-to-tail dimeric motifs as the building edge for constructing sophisticated 2D macrocyclic architectures. These dimeric motifs consist of dissymmetrical ditopic ligand systems, where one tpy unit is modified at the 6- or 5-position while the other is functionalized at the 4′-position. Through shape-complementary principles, this design enables highly selective formation of the head-to-tail dimeric motifs (100% selectivity). For instance, the ditopic ligand L36, featuring 5-position-modified tpy, was designed and synthesized. Upon coordination with Zn^2+^, Cd^2+^, or Fe^2+^, L36 selectively self-assembled into dimers (S48–S50), demonstrating metal-independent assembly behavior.^115^ By further extending the ligand design—specifically through additional tpy arm incorporation at the 5′′-position—ligand L37 was developed. Intriguingly, L37 formed tetrameric macrocycles S51 and S52 when assembled with labile Zn^2+^ or Cd^2+^ ions, whereas coordination with non-labile Fe^2+^ yielded a mixture of tetrameric S53, hexameric S54, and octameric S55 (Fig. 24).

**Fig. 25:**
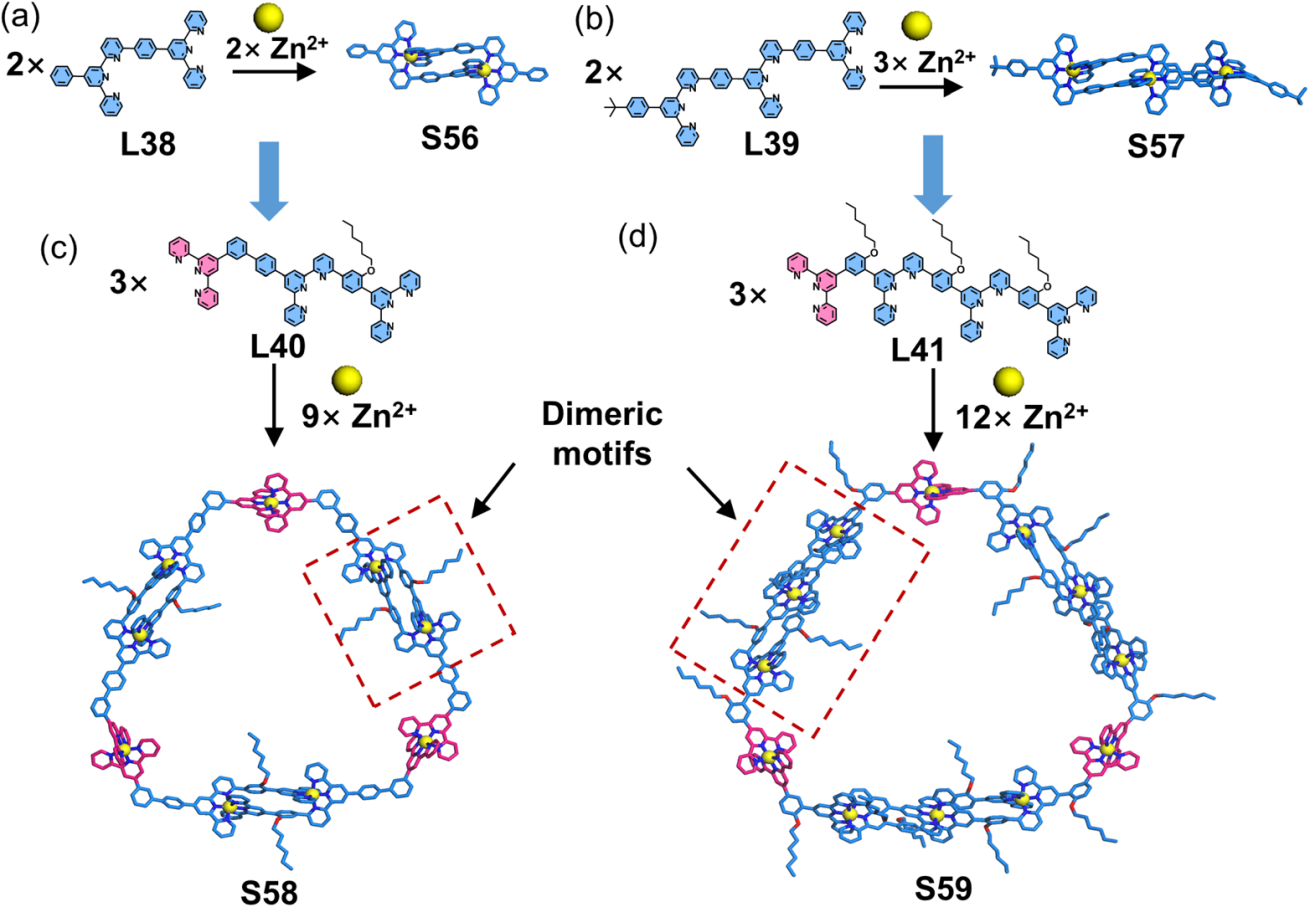
(a) Crystal structure of dimeric motif S56 assembled from ligand L38. (b) Crystal structure of dimeric motif S57 assembled from ligand L39. (c) Simulated structure of polygon S58 assembled from ligand L40. (d) Simulated structure of polygon S59 assembled from ligand L41.

The same group reported another example of dimeric motifs based on 6-position-modified tpy units.^83,116^ Ladder-type ditopic ligand L38 and tritopic ligand L39, both functionalized at the 6-position, were designed and synthesized. Upon coordination with Zn^2+^ ions, L38 and L39 selectively formed head-to-tail dimeric motifs S56 and S57 (100% selectivity), respectively. Furthermore, using meta-substituted benzene as a directing unit, dissymmetrical ligands L40 and L41 with varying arm lengths were developed. These ligands self-assembled with Zn^2+^ ions to generate dimer-based hexagonal macrocyclic structures S58 and S59 (Fig. 25).

**Fig. 26:**
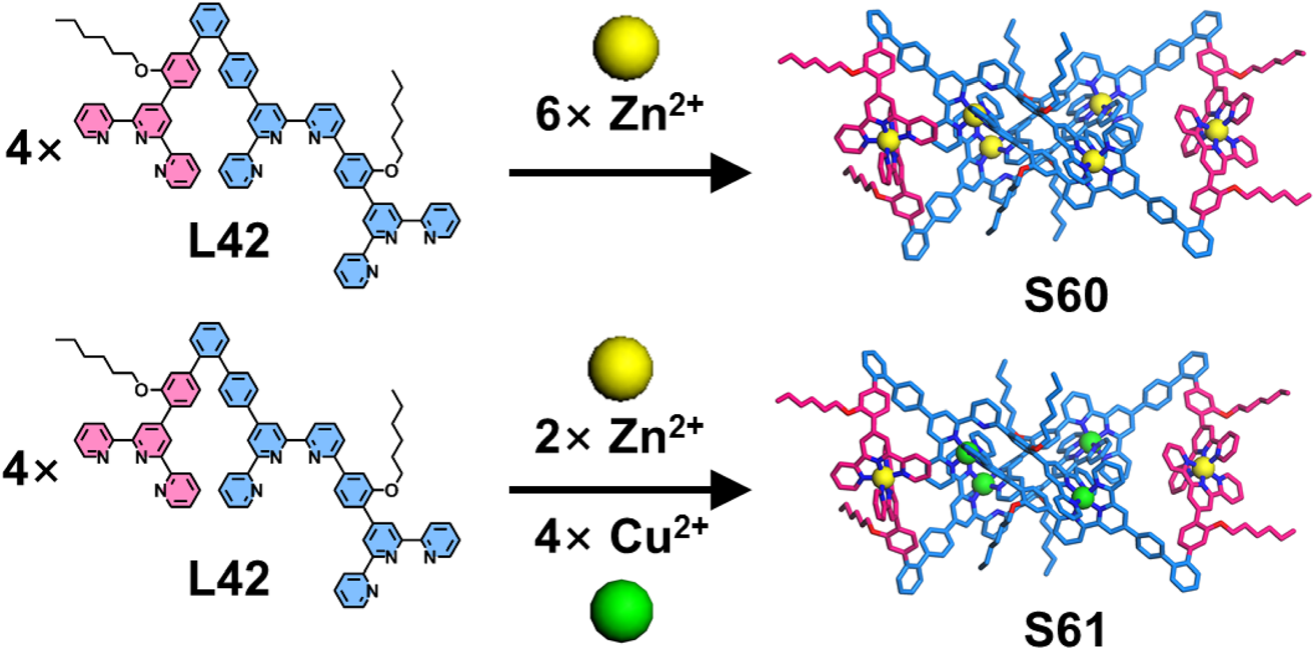
Simulated structures of figure-of-eight macrocycles S60 and S61 assembled from ligand L42.

Building upon this foundation, Wang and coworkers employed ortho-substituted benzene rings as directing units to design and synthesize a 60°-directed dissymmetrical ligand L42, featuring both ditopic and monotopic ligand arms (Fig. 26). Typically, 60°-directed ligands can form trimeric triangular architectures. While the dissymmetrical design of L42—with two arms of unequal length—led to different behavior. Upon coordination with Zn^2+^ ions, the ditopic ligand arm exhibited high self-selectivity, resulting in the exclusive formation of tetrameric macrocycle S60, as confirmed by NMR and ESI-MS. To alleviate steric strain caused by the tetramer, S60 adopted a twisted figure-of-eight conformation. Notably, the 6-position-modified benzene rings in the dimeric motif engaged in cation-π interactions with metal centers, significantly enhancing binding affinity for Cu^2+^ ions and altering the metal selectivity profile. Leveraging this effect, the authors achieved precise one-pot synthesis of heterometallic macrocycle S61 by mixing L42 with both Zn^2+^ and Cu^2+^ ions with 100% metal selectivity.^117^

**Fig. 27:**
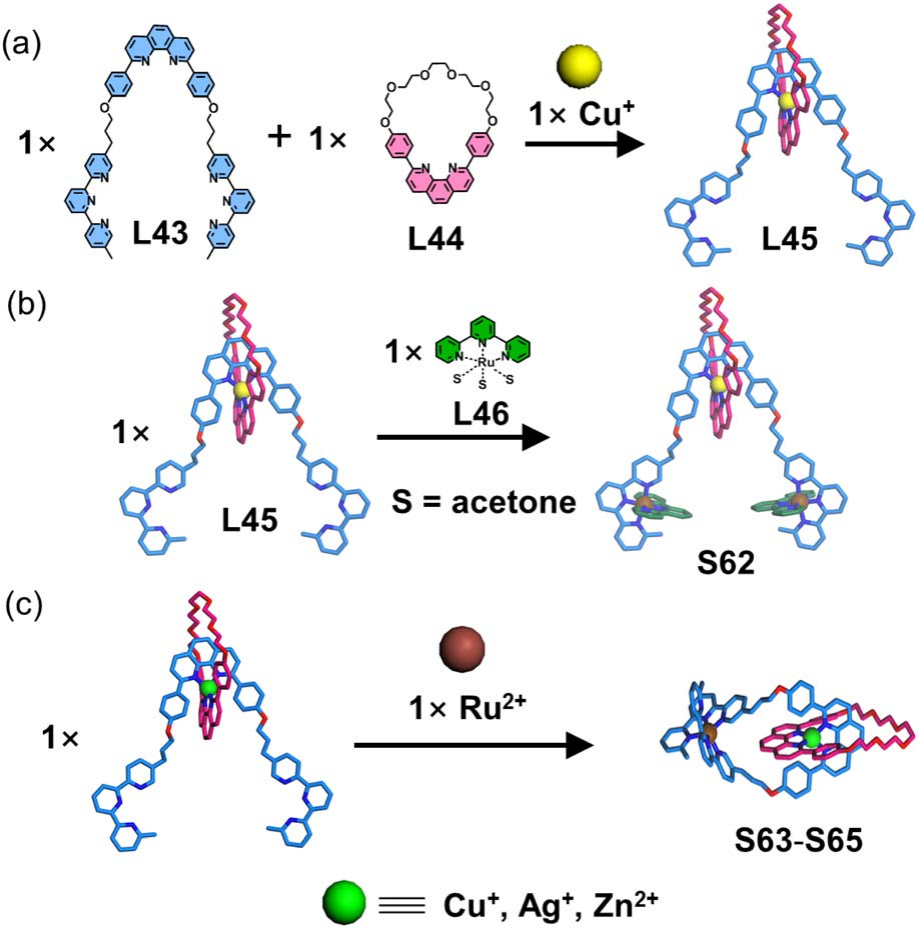
(a) Simulated structure of metallo-ligand L45 assembled from ligands L43 and L44. (b) Simulated structure of rotaxane S62 assembled from ligands L45 and L46. (c) Simulated structures of catenanes S63–S65 assembled from their respective ligands.

Tpy ligands, with their octahedral coordination geometry, form metal complexes that serve as ideal crossing points. The multiple modifiable sites on the side pyridines of tpy enable ring-closing linkages for the synthesis of specific macrocyclic molecules, including twisted macrocycles, catenanes, and knots. In earlier work, Sauvage and coworkers reported an orthogonal self-assembly strategy employing tpy and diphenylphenanthroline (dpp) motifs to construct sophisticated macrocyclic architectures. The construction of catenanes and rotaxanes was achieved through a dpp-based central template, where tpy-metal complexes served as connecting units.^118^ The authors designed and synthesized ligand L43 containing two tpy units and one dpp unit (Fig. 27). This ligand first reacted with macrocycle L44 and Cu^+^ to form the pre-organized metallo-ligand L45 with intertwined topology. The remaining two tpy units then underwent either of two pathways: (1) reaction with stable metallo-ligand L46 to form rotaxane S62, or (2) coordination with Ru^2+^ ions to achieve ring closure, forming catenane S63. In subsequent studies, Ag^+^ or Zn^2+^ was employed as alternative metal centers to Cu^+^, demonstrating the formation of analogous catenane structures (S64/S65).^119^

**Fig. 28:**
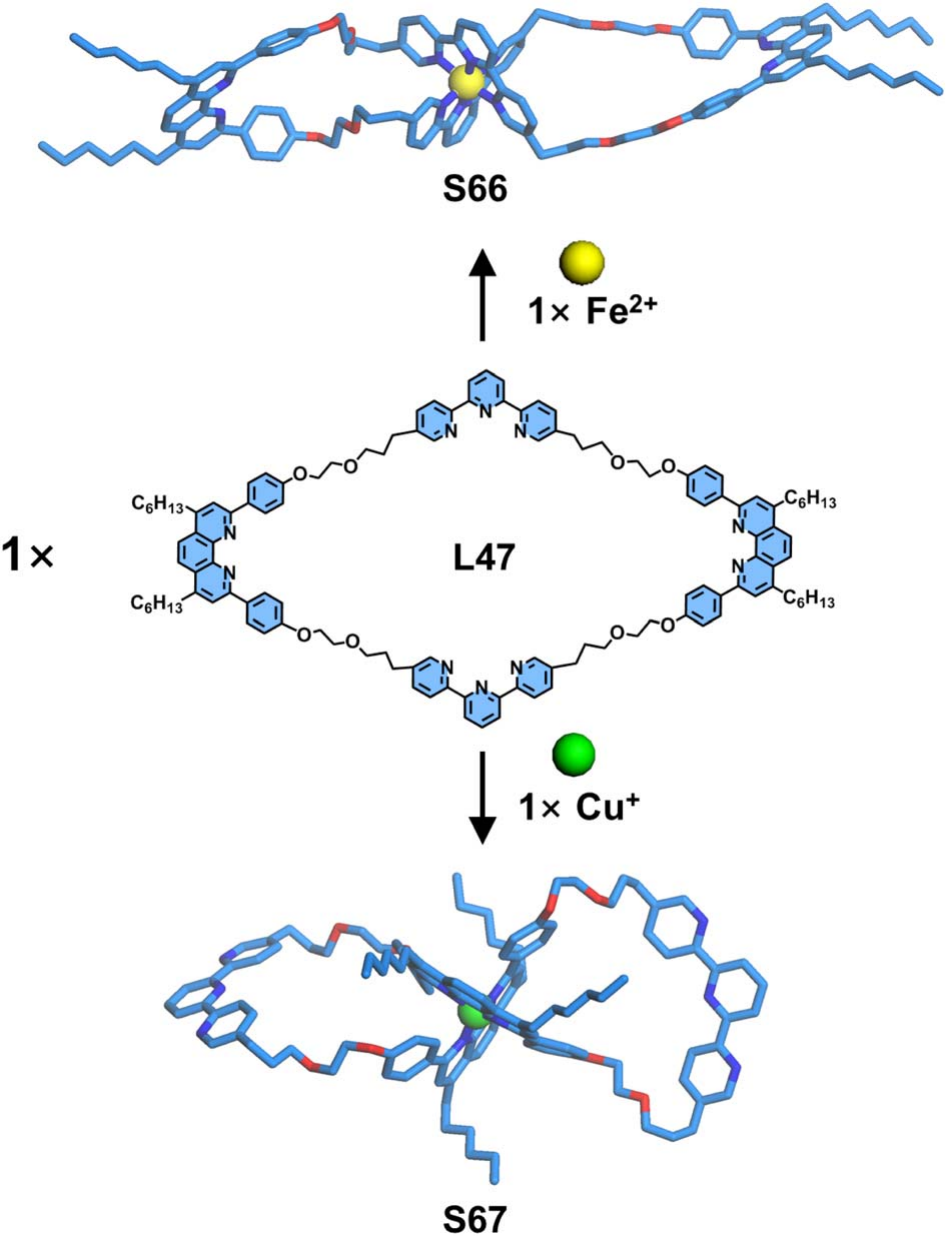
Simulated structures of figure-of-eight macrocycles S66 and S67 assembled from ligand L47.

Employing the same orthogonal coordination self-assembly strategy, Sauvage and coworkers achieved the design and synthesis of a dynamic figure-of-eight macrocyclic system (Fig. 28). The macrocyclic ligand L47 was judiciously designed to incorporate two dpp and two tpy units. The macrocyclic system exhibited metal-dependent structural conversion, yielding distinct figure-of-eight architectures through selective metal coordination: Fe^2+^ ions selectively coordinated to the tpy units, generating macrocycle S66 and Cu^+^ ions preferentially bound to dpp sites, forming macrocycle S67. Notably, upon electrochemical oxidation, S67 undergoes oxidation of Cu^+^ to Cu^2+^ followed by coordination to tpy units, resulting in an electrochemically triggered conformational switching of the figure-of-eight structures.^120^

**Fig. 29:**
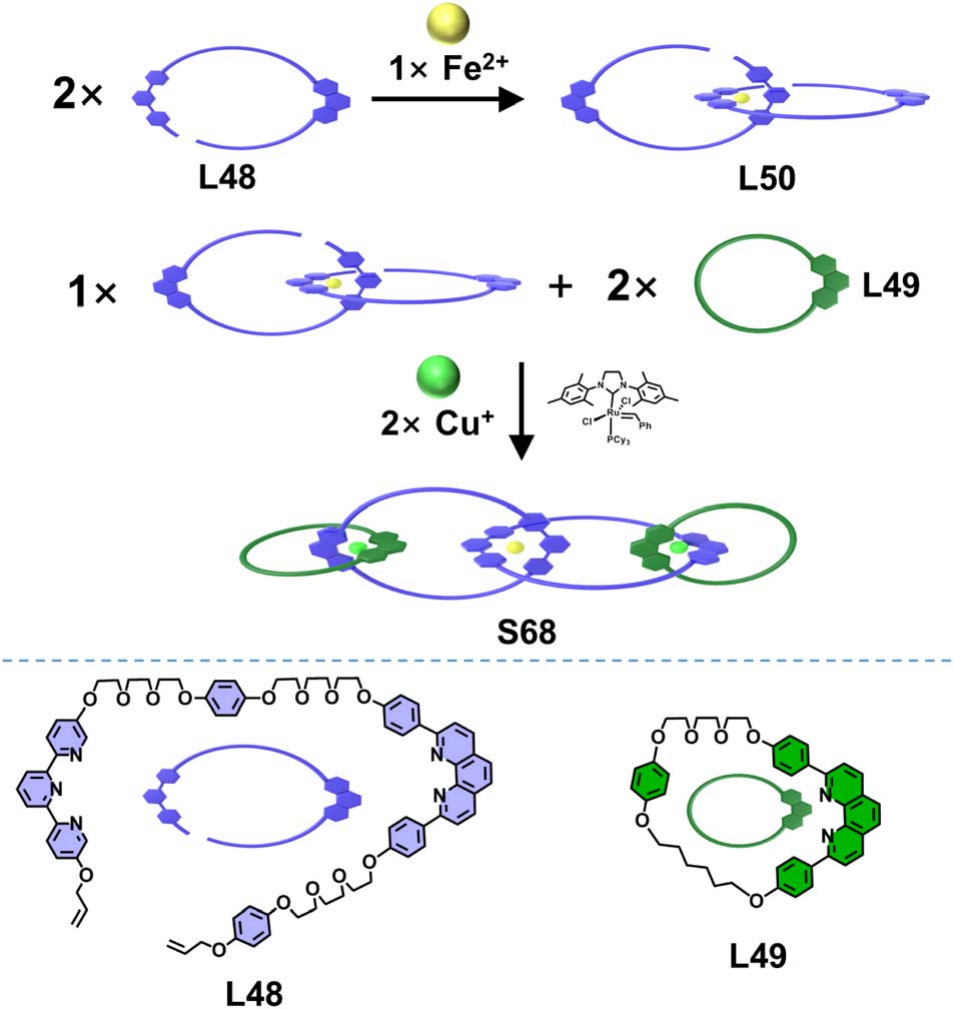
The illustration of the synthesis of [4]catenane S68.

**Fig. 30:**
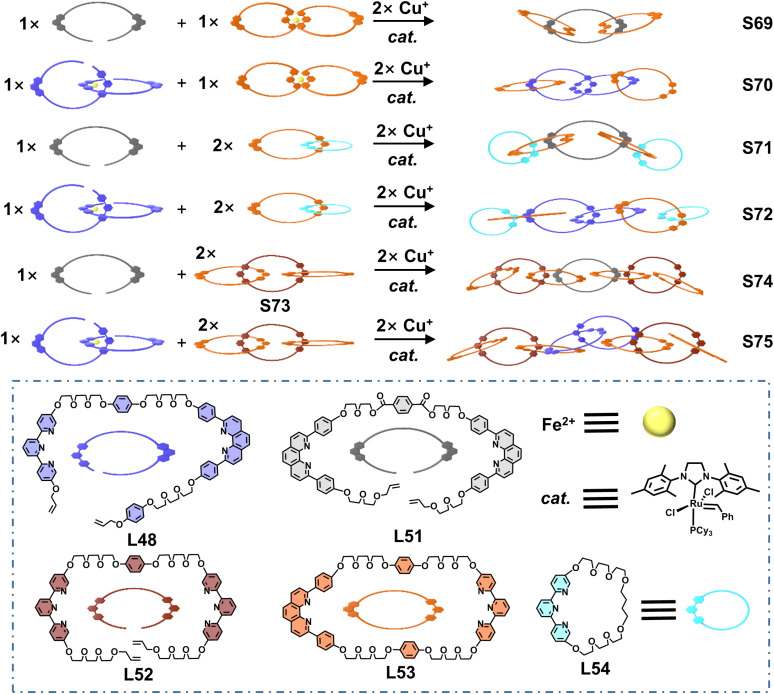
The illustration of the synthesis of [3–8]catenanes S69–S75.

By introducing multiple 〈tpy-M-tpy〉 and 〈dpp-M-dpp〉 coordination centers as crossing points, Barnes and coworkers reported the efficient synthesis of a series of linear [*n*]catenanes. An open macrocyclic ligand L48, containing one tpy and one dpp unit, was designed and synthesized. Fe^2+^ ions selectively coordinated with the tpy unit of L48 to form the open metallo-ligand L50, leaving two uncoordinated dpp units.^121^ Subsequent assembly of L50 with the macrocyclic ligand L49 and Cu^+^ ions, followed by ring-closing metathesis, afforded the [4]catenane S68 (Fig. 29). This excellent strategy was further extended to construct [*n*]catenanes (*n* = 3–8). The open macrocyclic ligands L48, L51, and L52 as well as the closed macrocyclic ligands L53 and L54 were judiciously designed, incorporating tpy and/or dpp units.^122,123^ The open ligands could thread through the closed macrocyclic ligands under coordination-directed templation, followed by ring-closing metathesis to afford the target [*n*]catenanes S69–S75 (Fig. 30).

**Fig. 31:**
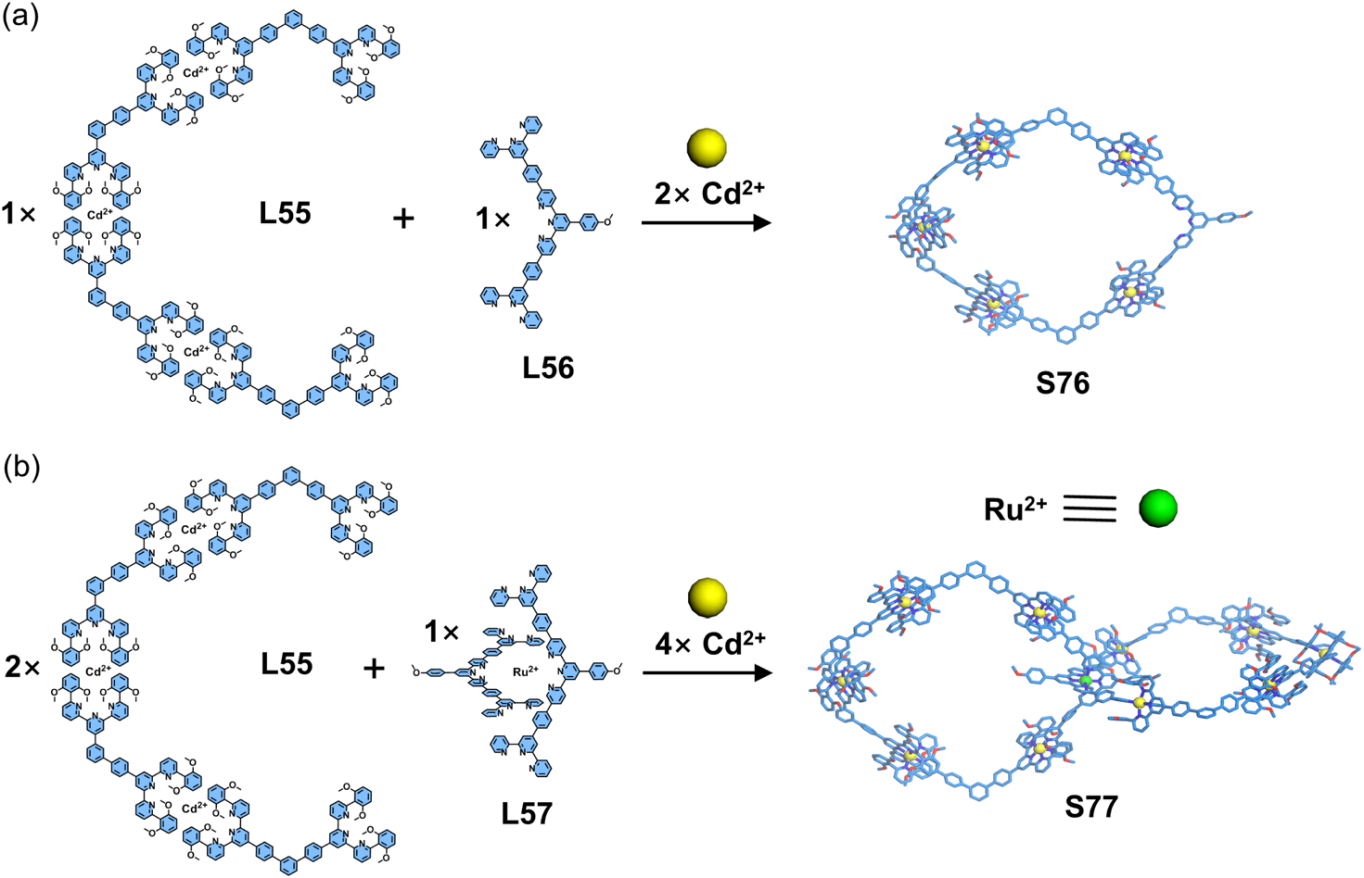
(a) Simulated structure of macrocycle S76 assembled from ligands L55 and L56. (b) Simulated structure of catenane S77 assembled from ligands L55 and L57.

Chan and coworkers developed a stepwise self-assembly strategy for the synthesis of a metallo-catenane (Fig. 31). The authors employed a combination of metal coordination and Suzuki–Miyaura coupling reactions to first construct metallo-ligand L55. Subsequent assembly of L55 with L56 and Cd^2+^ ions yielded metallacycle S76, which contained an uncoordinated tpy unit. In a parallel approach, ligand L56 was pre-coordinated with Ru^2+^ to establish the 〈tpy-Ru-tpy〉 crossing point, forming metallo-ligand L57. The final metallo-catenane S77 was then obtained through the assembly of L57 with L55 and Cd^2+^ ions.^124^

**Fig. 32:**
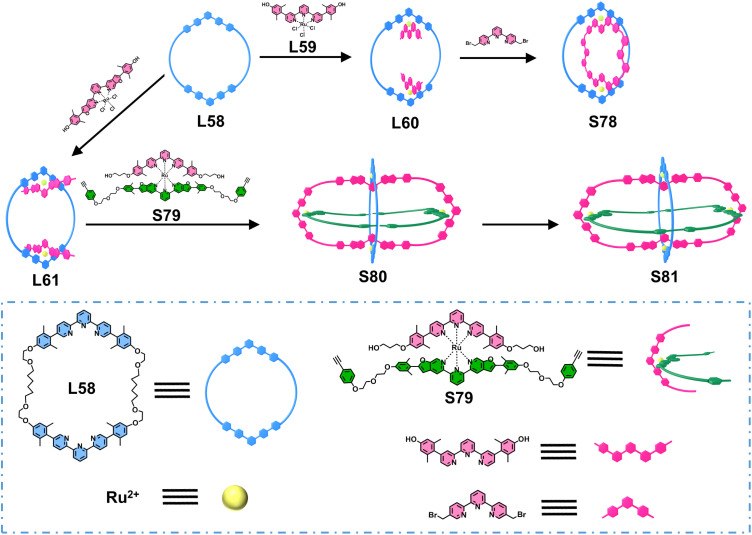
The illustration of the synthesis of ring-in-ring complex S78 and Borromean link S81.

Siegel and coworkers reported a precise strategy for constructing ring-in-ring structures and Borromean links (Fig. 32). The authors designed a macrocyclic ligand L58 featuring two tpy units, which reacted with a Ru^2+^-based metallo-ligand L59 to form stable complex L60. The remaining hydroxyl groups subsequently underwent ring closure *via* Williamson ether synthesis with 5,5′′-bis(bromomethyl)-terpyridine, yielding the ring-in-ring complex S78.^125^ Although the uncoordinated tpy units in S78 could potentially participate in further metal coordination, attempts to extend this system to synthesize Borromean links were unsuccessful. Subsequently, employing a similar strategy, the authors synthesized another metallo-ligand L61 structurally analogous to L60. This ligand reacted with mononuclear complex S79 to form intermediate S80, leaving acetylene groups available for intramolecular macrocyclization through a copper-mediated Eglinton reaction, ultimately achieving the synthesis of Borromean link S81.^126^

**Fig. 33:**
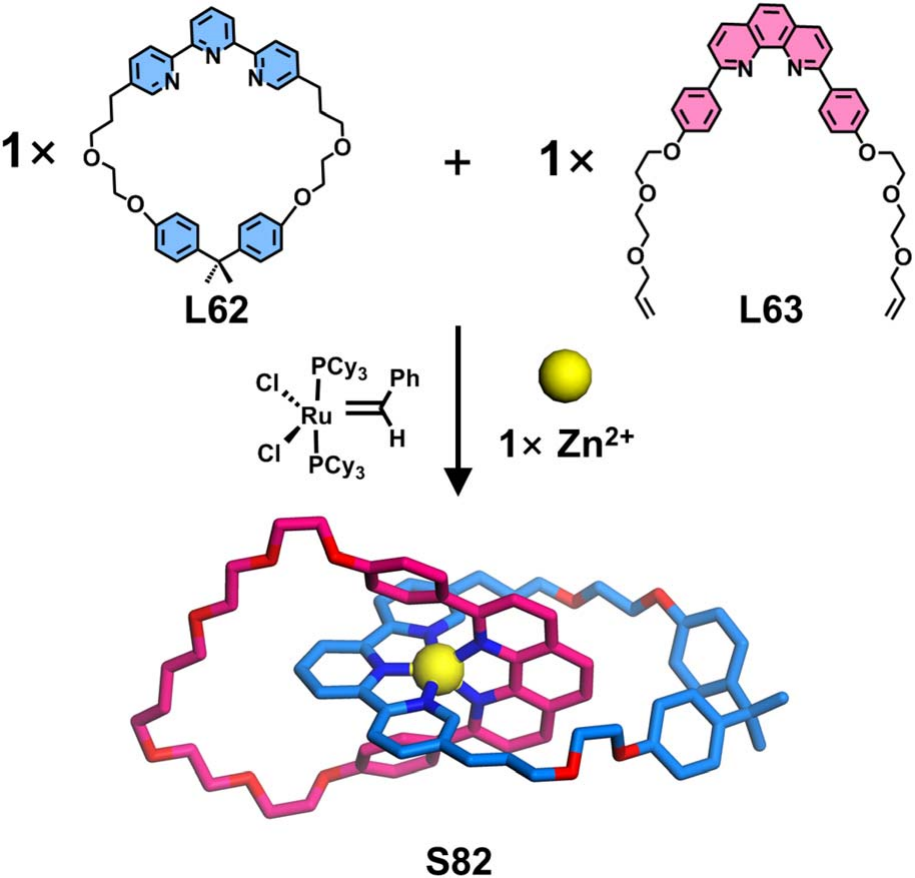
The illustration of the synthesis of [2]catenane S82 (simulated structure).

In addition to orthogonal self-assembly strategies for constructing crossing points based on 〈tpy-M-tpy〉 or 〈dpp-M-dpp〉 coordination centers, Sauvage, Kern, and coworkers reported a precise synthesis of [2]catenanes utilizing <tpy-M-dpp> as the crossing point (Fig. 33). The macrocyclic ligand L62 containing a single tpy unit was first Zn^2+^-coordinated, followed by the introduction of open macrocyclic ligand L63 bearing one dpp unit. Through metal-ion templating effects, L63 underwent threading through the cavity of L62. The subsequent ring-closing metathesis of the two remaining terminal olefins, mediated by Grubbs first-generation catalyst, afforded the [2]catenane S82.^127^

**Fig. 34:**
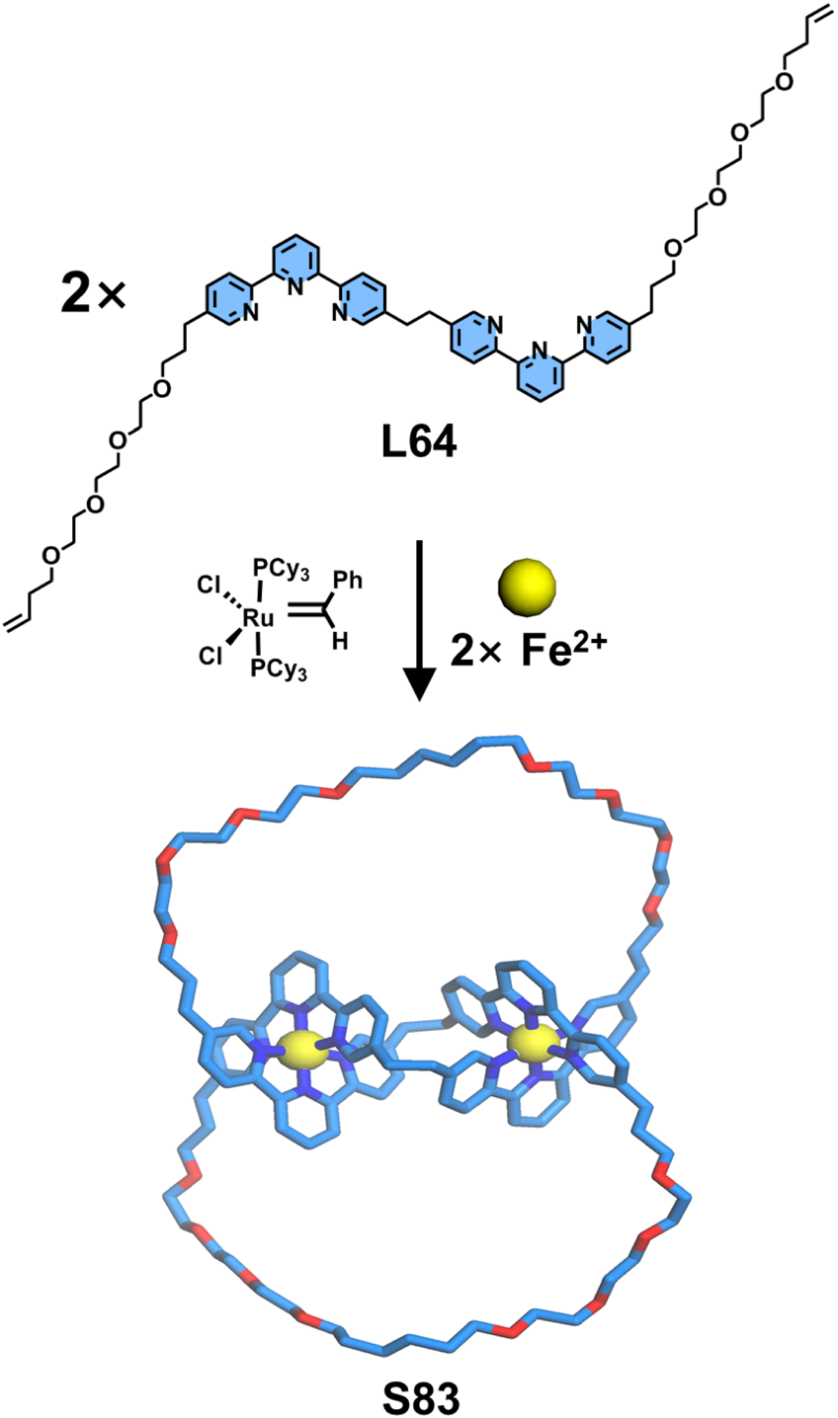
The illustration of the synthesis of knot S83 (simulated structure).

Molecular knots, as a class of mechanically interlocked macrocyclic molecules similar to catenanes, are typically formed through the topological entanglement of a single molecular strand, requiring judiciously designed crossing points for their construction. Sauvage and coworkers reported a seminal synthesis of molecular knots employing 〈tpy-M-tpy〉 templating strategy (Fig. 34). Ligand L64 was structurally engineered with two tpy units interconnected at their 5-positions *via* flexible alkyl chains, conferring adequate conformational flexibility to facilitate helical twist. The remaining 5′′-positions of the tpy units were functionalized with molecular chains containing terminal olefins. Upon coordination with Fe^2+^ ions, L64 self-assembled into a double-stranded helical precursor. Subsequent ring-closing metathesis of the terminal olefins under Grubbs catalysis yielded the topologically complex molecular knot S83.^128^

### 3.4 3D cages and polyhedra

In addition to 2D architectures, the coordination-driven self-assembly of tpy-based ligands and metal ions has been extensively employed for constructing 3D supramolecular architectures.^129–132^ Owing to the linear geometry of 〈tpy-M-tpy〉 connectivity, tpy moieties are normally positioned along the edges of 3D polyhedra. The design principles for tpy-based ligands typically involve covalent conjugation of directing groups with three or more substitutable sites to the 4′-position of tpy units to construct multitopic ligands, while ditopic ligands are generally employed for 2D architectures. The resulting 3D architectures exhibit strong dependence on the geometry of the ligands, including arm length, arm multiplicity, and inter-arm angular disposition. Guided by these fundamental design criteria, numerous tpy-based 3D supramolecular systems have been successfully engineered, as systematically documented in several seminal review articles.^70,72,74,75^

**Fig. 35:**
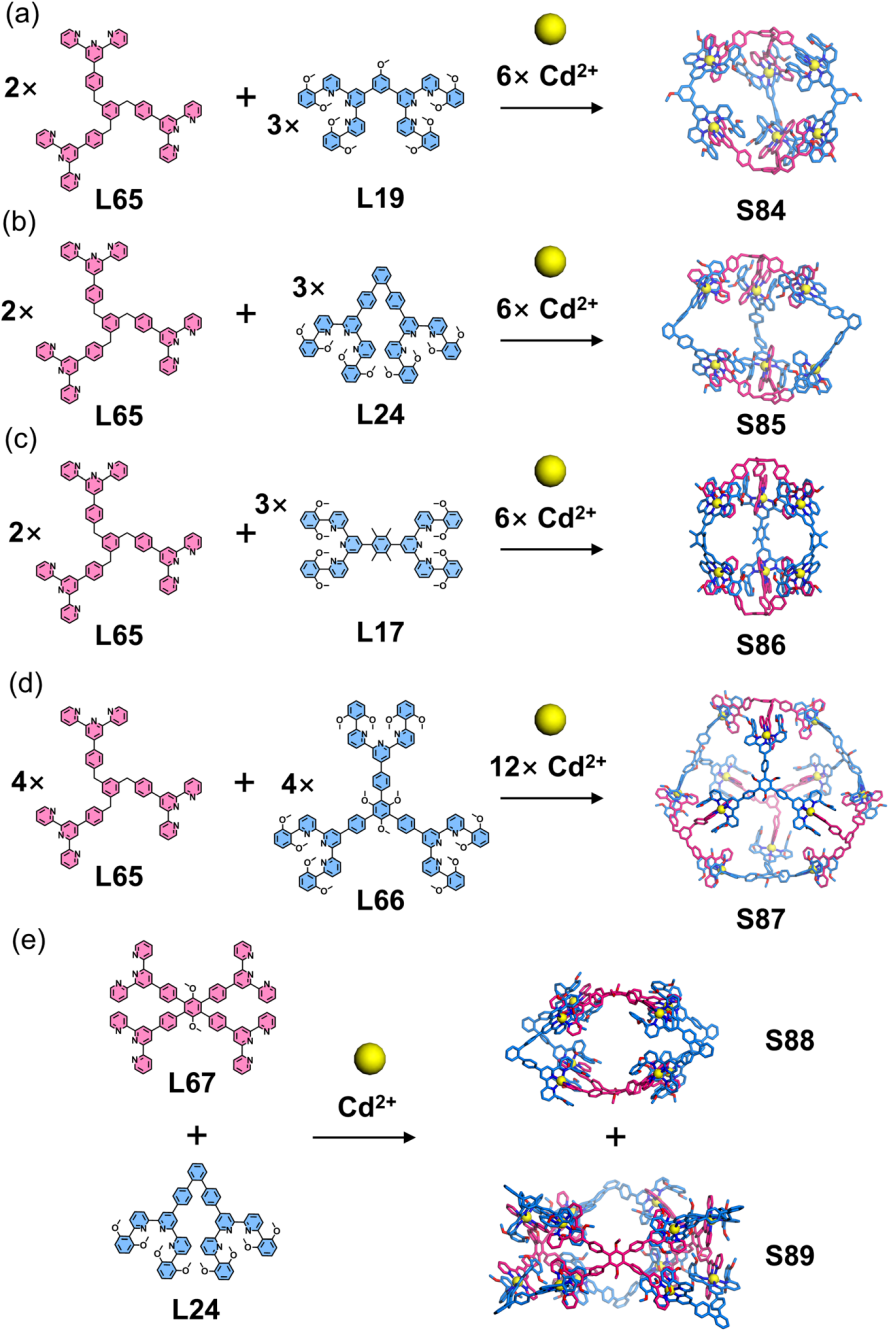
(a) Simulated structure of cage S84 assembled from ligands L19 and L65. (b) Simulated structure of cage S85 assembled from ligands L24 and L65. (c) Simulated structure of cage S86 assembled from ligands L17 and L65. (d) Simulated structure of cage S87 assembled from ligands L65 and L66. (e) Simulated structures of cages S88 and S89 assembled from ligands L24 and L67.

Chan and coworkers employed complementary ligand pairs to construct a series of heteroleptic 2D architectures, as described in Section 3.3. By extending the design to multitopic ligands, they further obtained a family of heteroleptic 3D structures (Fig. 35). Specifically, the assembly of a tritopic ligand (L65) with ditopic ligands (L19, L24, and L17) in the presence of Cd^2+^ yielded heteroleptic cages S84–S86.^106^ Expanding this strategy, the combination of two tritopic ligands (L65 and L66) with Cd^2+^ produced a dodecanuclear Cd^2+^ tetrapod structure S87. Using a similar strategy, the combination of a tetratopic ligand (L67) with a ditopic ligand (L24) and Cd^2+^ ions afforded a mixture of heteroleptic cages (S88 and S89).^112^

**Fig. 36:**
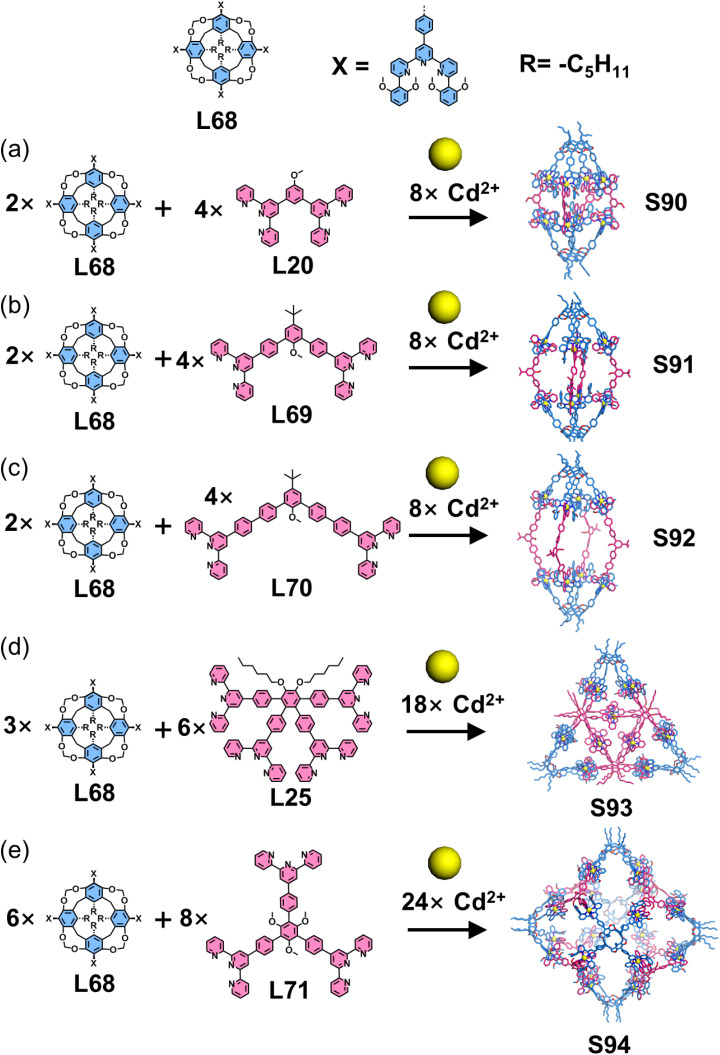
(a) Simulated structure of cage S90 assembled from ligands L20 and L68. (b) Simulated structure of cage S91 assembled from ligands L68 and L69. (c) Simulated structure of cage S92 assembled from ligands L68 and L70. (d) Simulated structures of cage S93 assembled from ligands L25 and L68. (e) Simulated structures of cage S94 assembled from ligands L68 and L71.

**Fig. 37:**
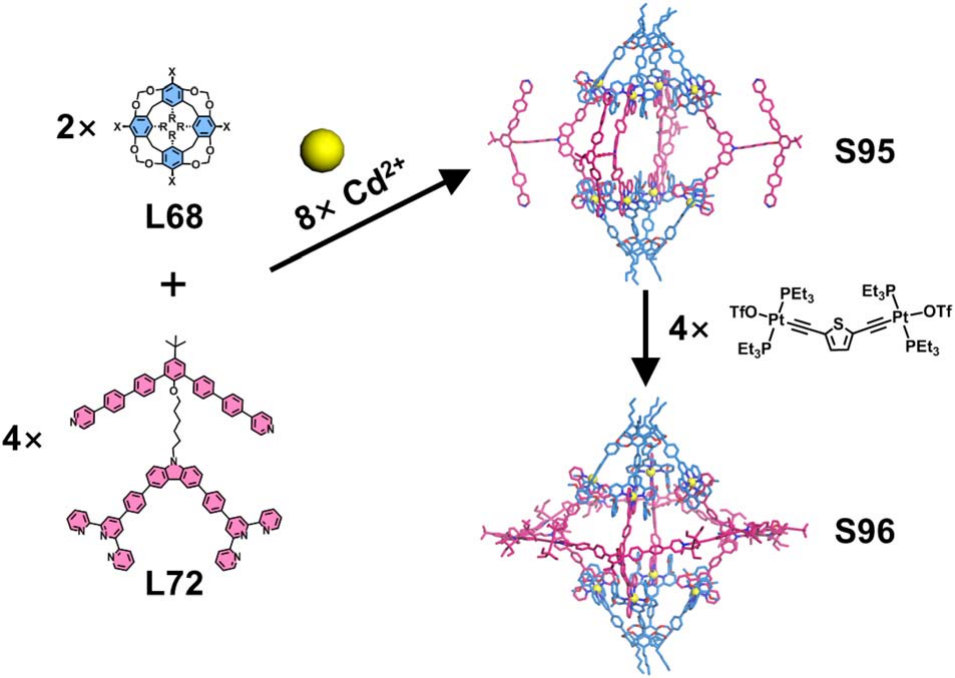
Simulated structures of cage S95 and nano-Saturn complex S96 assembled from ligands L68 and L72.

Macrocyclic arenes and their related compounds—such as calixarenes, pillararenes, pyrogallolarenes, and resorcinarenes—are frequently employed as directing units for constructing 3D cages due to their high symmetry, suitable structural rigidity, tunable functionalization, and versatile substitutable sites. Chan and coworkers employed calix[4]resorcinarene as a structural directing unit to construct heteroleptic 3D architectures (Fig. 36). The tetratopic calix[4]resorcinarene-based ligand L68 was designed and synthesized *via* Suzuki–Miyaura coupling between tetrabrominated methylene-bridged calix[4]resorcinarene and 6,6′′-substituted terpyridylphenylboronic acid pinacol ester. Assembly of L68 with 120°-directed ditopic ligands (L20, L69, and L70) and Cd^2+^ ions yielded three distinct 3D nanocapsules (S90–S92). Extending this strategy, self-assembly of L68 with tetratopic ligand L25 or tritopic ligand L71 in the presence of Cd^2+^ produced a Sierpiński triangular prism S93 or a cubic star S94, respectively.^133^ Building upon the 3D nanocapsule architecture, the same group further functionalized the ditopic ligands by incorporating two pyridyl motifs to construct the outer macrocycles, culminating in the design of tetratopic ligand L71.^134^ Using a stepwise assembly strategy, the authors first pre-assembled nanocapsule S95 from L68, L72, and Cd^2+^ ions. The remaining pyridyl units were then coordinated to organometallic *trans*-Pt^2+^ acceptors, ultimately yielding heterobimetallic nano-Saturn complex S96 (Fig. 37).

**Fig. 38:**
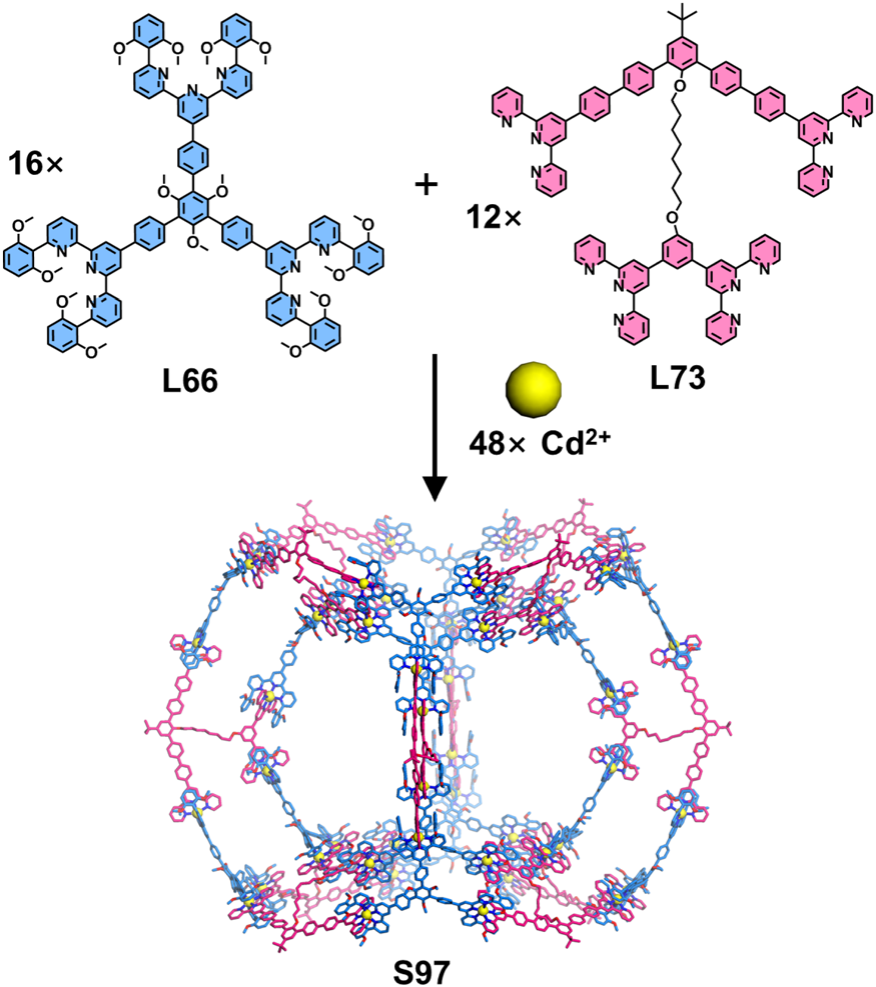
Simulated structure of double-layered cuboctahedron S97 assembled from ligands L66 and L73.

The construction of 3D supramolecular architectures with precisely enlarged sizes remains a significant synthetic challenge. Chan, Liou, and coworkers addressed this by integrating complementary ligand pairs with multivalent ligand design, successfully synthesizing a giant double-layered cuboctahedron (S97) featuring inner and outer diameters of 6.3 nm and 10.5 nm, respectively (Fig. 38). Key to this achievement was the strategic design of tetratopic ligand L73, incorporating flexible C8 alkyl chains as a linker. Assembly of L73 with tritopic L66 and Cd^2+^ ions yielded exclusively the target structure S97, as unambiguously confirmed by NMR and ESI-MS. Crucially, the length of the alkyl chains proved determinant: analogous ligands with shorter C6 or longer C12 linkers failed to exclusively produce the target cuboctahedron, highlighting the precision required in spacer engineering.^135^

**Fig. 39:**
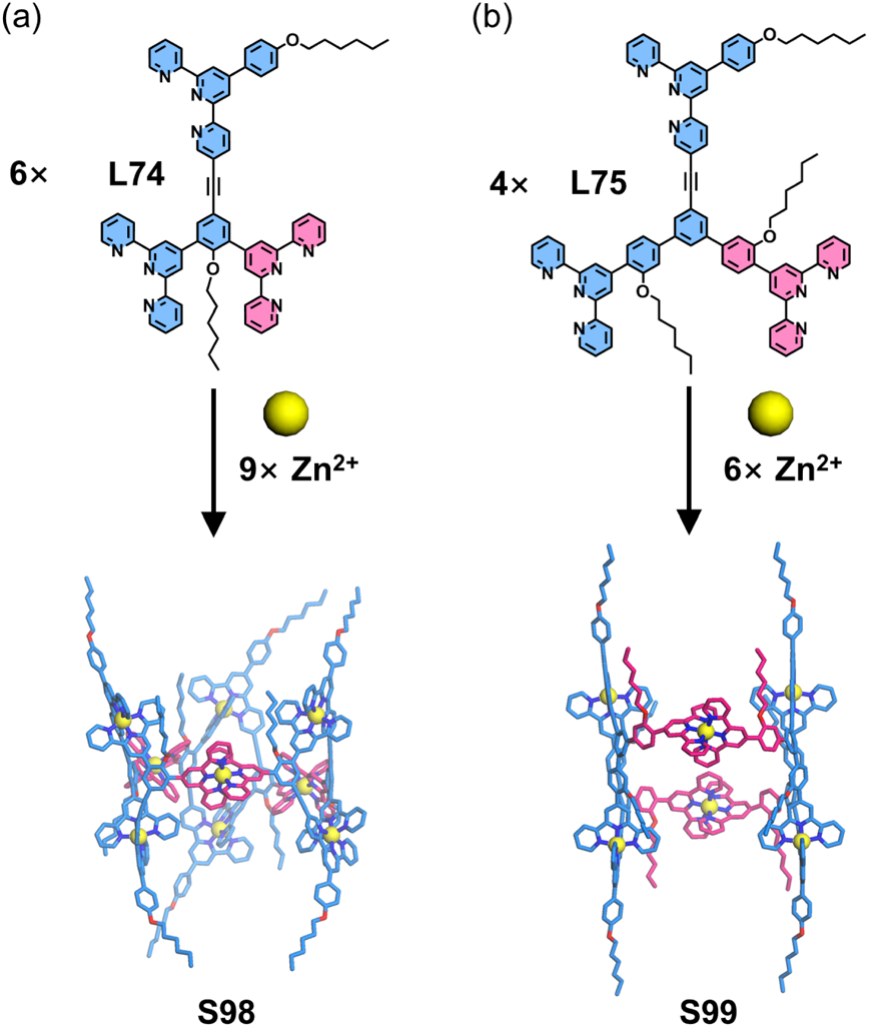
(a) Simulated structure of cage S98 assembled from ligand L74. (b) Simulated structure of cage S99 assembled from ligand L75.

Compared to conventional 4′-position modifications, substitutions at the side pyridine positions provide an alternative strategy for angular modulation, significantly enriching ligand geometries and facilitating the development of novel 3D architectures. Furthermore, dissymmetrical functionalization of one-side pyridine in the tpy units can effectively reduce ligand symmetry, thereby enabling the construction of low-symmetry and chiral 3D architectures. Wang, Song, Wang, and coworkers reported the construction of a low-symmetry hourglass-shaped molecular cage through precisely designed tritopic ligand L74, which incorporated two 4′-substituted and one 5-substituted tpy units (Fig. 39). The coordination between one 4′-substituted tpy unit and the 5-substituted tpy unit with Zn^2+^ ions formed a self-complementary dimer, while the remaining tpy units participated in macrocyclic coordination. The introduction of this self-complementary dimer effectively reduced the structural symmetry and expanded the architectural dimensionality, ultimately leading to the formation of a hexameric coordination cage S98. Notably, when the ligand arm length was extended in the designed ligand L75, the assembly with Zn^2+^ yielded a tetrameric structure S99 instead of the hexameric form of L75. Mechanistic studies revealed that this structural divergence could originate from preformed complementary dimers during the assembly process, where the increased ligand arm length induced a change in the metal–ligand arm angle, thereby altering the final 3D architecture.^136^

**Fig. 40:**
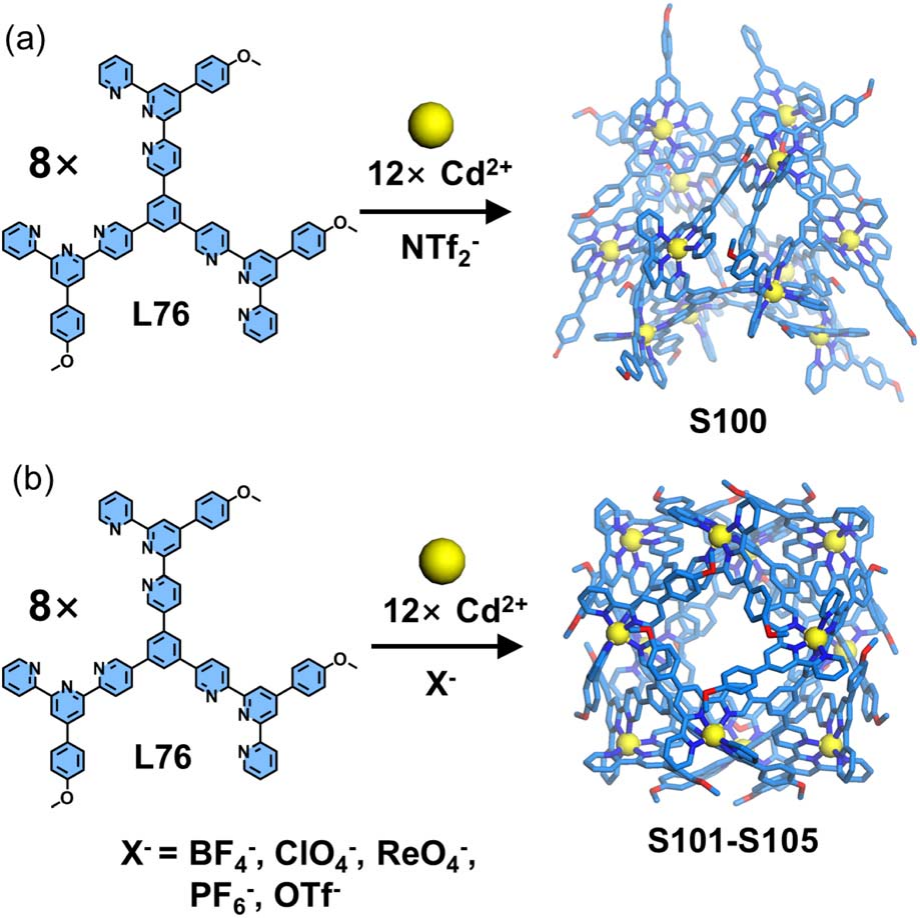
(a) Crystal structure of cage S100 assembled from ligand L76 with ion templating. (b) Crystal structures of cages S101–S105 assembled from ligand L76 with ion templating.

Wu, Wang, Hou, and coworkers reported the synthesis of a class of coordination polyhedra exhibiting anion-dependent structural polymorphism (Fig. 40). The authors designed and synthesized a tritopic dissymmetrical ligand L76 featuring three 5-position modified tpy units. The self-assembly of L76 with Cd^2+^ demonstrates significant anion-dependent structural regulation. When employing the bulkier NTf_2_^−^ anion, its templating effect facilitates the formation of a *D*_3_-symmetric *quasi*-icosahedral architecture S100. In contrast, smaller anions including BF_4_^−^, ClO_4_^−^, ReO_4_^−^, PF_6_^−^, and OTf^−^ induce transformation into a highly symmetric cuboctahedral architectures S101–S105. In contrast to conventional strategies, 5-position functionalization confers directional characteristics to the 〈tpy-M-tpy〉 coordination center, enabling its role as polyhedral vertices in the 3D polyhedral architecture. Meanwhile, the 5-position substituted tpy exhibits greater structural rigidity, facilitating intermolecular stacking and the acquisition of single-crystal structures of metallo-supramolecules.^34^

**Fig. 41:**
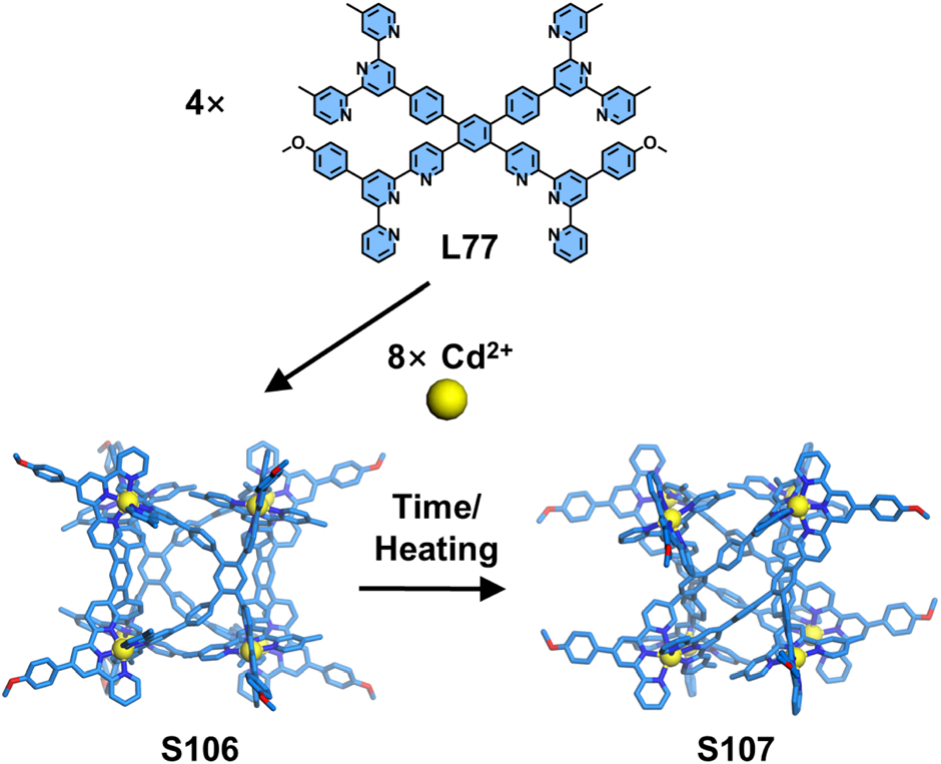
Simulated structure of cage S106 and crystal structure of cage S107 assembled from ligand L77 and their configurational interconversion from S106 to S107.

Wang, Wu, Jiang, and coworkers reported the construction of a 3D supramolecular cube (Fig. 41). The tetratopic ligand L77 was strategically designed with hybrid 5-substituted and conventional 4′-substituted tpy units, creating pronounced dissymmetry in the ligand geometry. The self-assembly of L77 with Cd^2+^ yielded two configurational cubic isomers (*C*_2h_-symmetric S106 and *D*_2_-symmetric S107), as unequivocally characterized by NMR, ESI-MS, and SCXRD. Under heating at 65 °C, time-resolved NMR studies revealed a slow configurational interconversion from *C*_2h_-S106 to *D*_2_-S107 with a remarkably low rate constant, achieving full transformation over four weeks.^137^

**Fig. 42:**
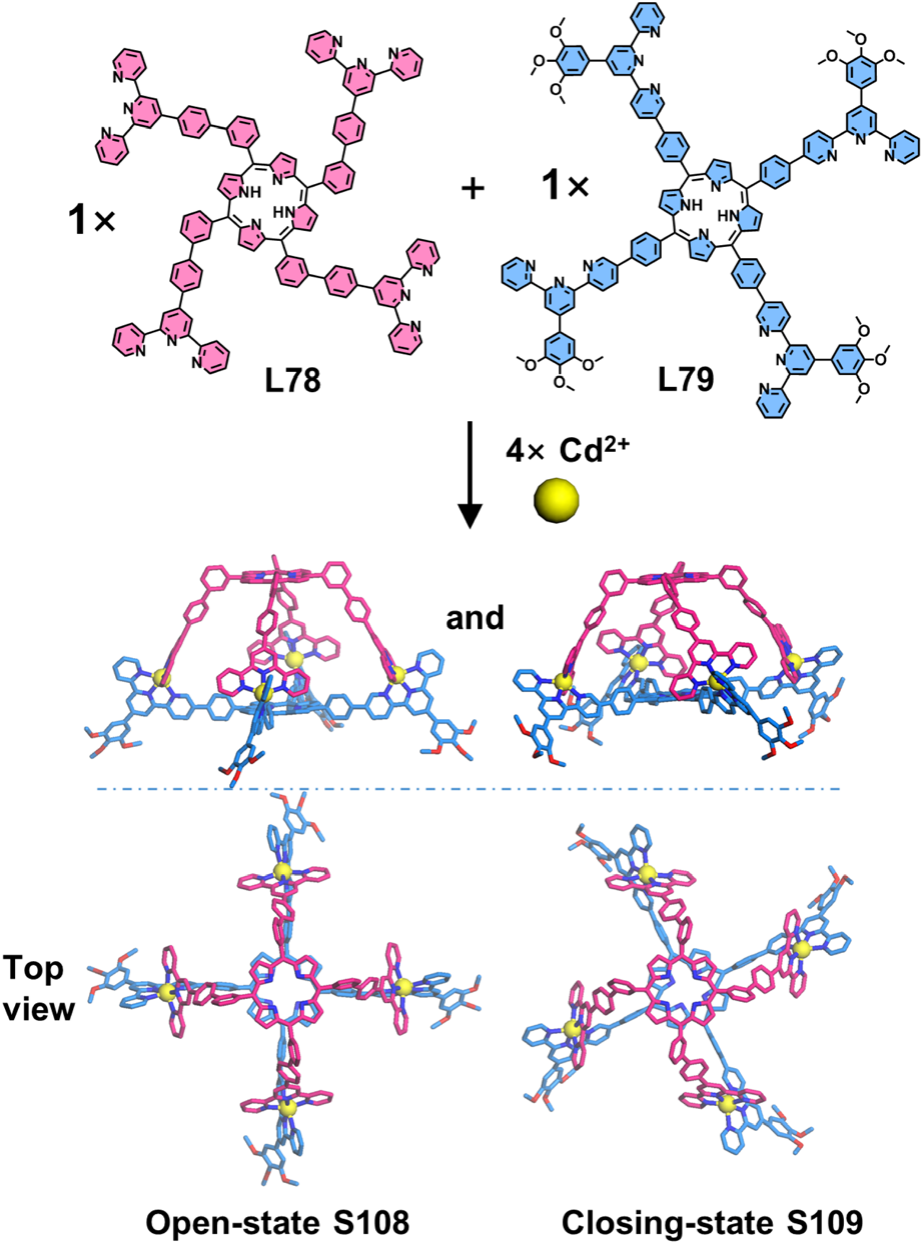
Crystal structures of cages S108 and S109 assembled from ligands L78 and L79.

In addition to constructing homoleptic 3D architectures, the side-pyridine functionalization strategy can precisely engineer complementary heteroleptic architectures through flexible angular modulation. Wang, Lin, Liu, Wang, and coworkers reported the precise construction of a porphyrinic heteroleptic cage. The authors designed two shape-complementary ligands: a 4′-position-modified tetratopic L78 and a 5-position-modified tetratopic ligand L79 (Fig. 42). Upon coordination with Cd^2+^, L78 and L79 self-assembled into the target heteroleptic cage with 100% ligand selectivity. SCXRD revealed that the heteroleptic cage features a truncated pyramid-shaped pocket, with the 〈tpy-M-tpy〉 coordination center positioned at the vertices to guide the assembly. Interestingly, the crystal structures exhibited two distinct conformations of the cage with varying cavity dimensions and helicity: an open-state S108 and a closed-state S109. The open-state S108 displays lower helicity (13°) and a more spacious cavity (interpanel distance of 14.0 Å), compared to those of closed-state S109 (39°, 12.6 Å). This observation highlights the tunable molecular cavity of the cage, suggesting its potential for applications in molecular recognition.^138^

### 3.5 Decker architectures

Coordination interactions enable the integration of ligands into discrete architectures, achieving their precise spatial positioning and ordered arrangement. Leveraging this advantage, the introduction of functional groups (*e.g.*, luminogens,^139–141^ photoswitches,^142–144^ catalytic centers,^145^ radicals,^146,147^ therapeutic agents,^148–150^ chiral groups^151–153^) into ligands enables their ordered spatial arrangement upon coordination, allowing for synergistic interactions among the groups and enhanced functionalities. Based on this strategy, numerous functionalized coordination supramolecular systems have been extensively reported, demonstrating application potential across diverse fields. To date, the majority of functionalized systems are based on macrocyclic or molecular cage frameworks featuring sizable cavities. Due to the relatively large geometric angles of directing units and metal centers, the incorporated functional groups occupy spatially dispersed positions, functioning as independent units without exhibiting strong interactions.

**Fig. 43:**
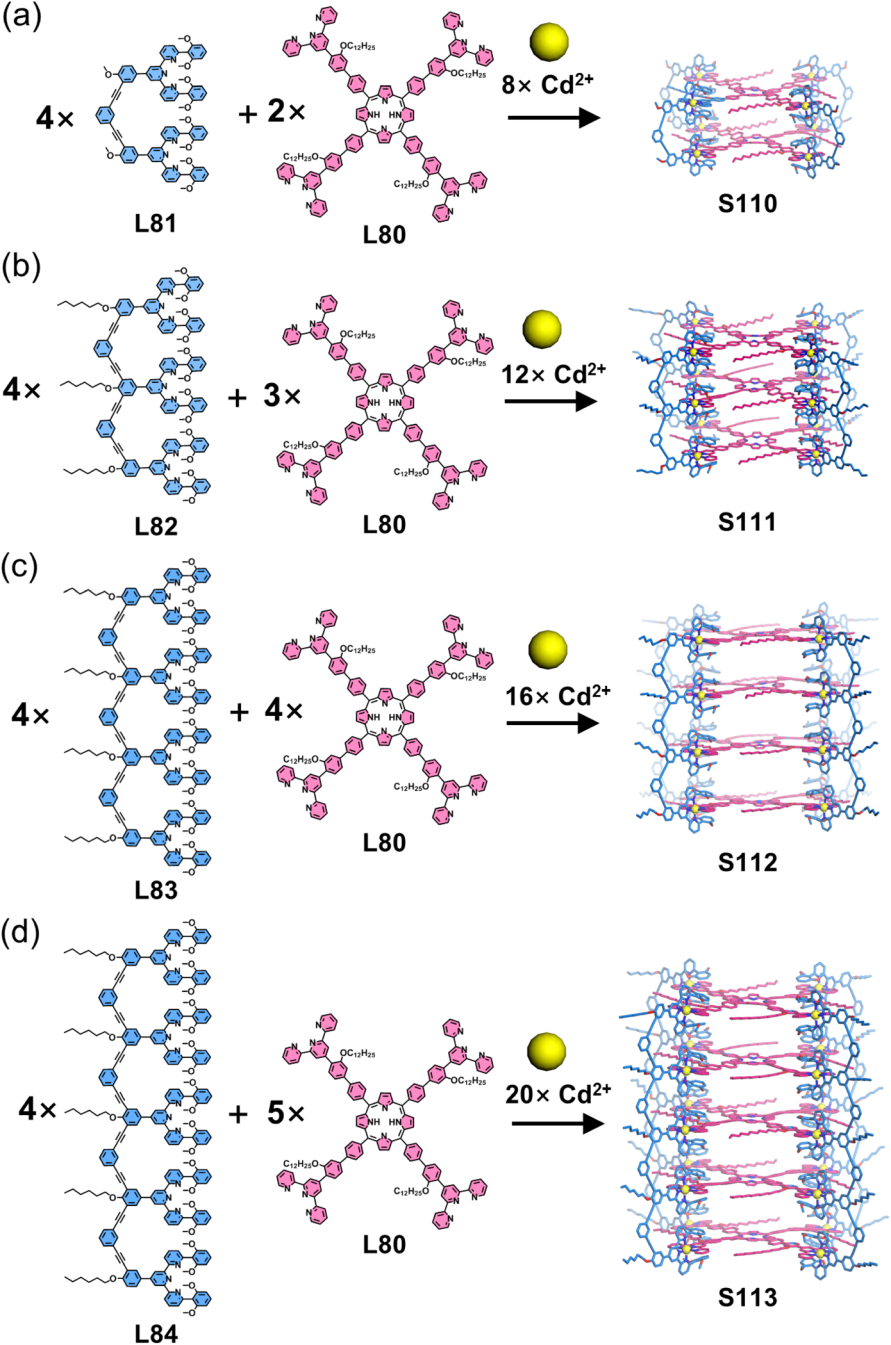
(a) Simulated structure of cage S110 assembled from ligands L80 and L81. (b) Simulated structure of cage S111 assembled from ligands L80 and L82. (c) Simulated structure of cage S112 assembled from ligands L80 and L83. (d) Simulated structures of cage S113 assembled from ligands L80 and L84.

Liu, Wang, and coworkers constructed a series of decker architectures with loose arrangements of functional units, characterized by large interlayer spacing.^154^ Using porphyrin-based tetratopic ligand L80 as the panel and ditopic L81, tritopic L82, tetratopic L83, and pentatopic L84 as edge components, the heteroleptic self-assembly of panel L74, edge ligands (L81–L84) with Cd^2+^ ions produced decker architectures S110–S113 (Fig. 43).

**Fig. 44:**
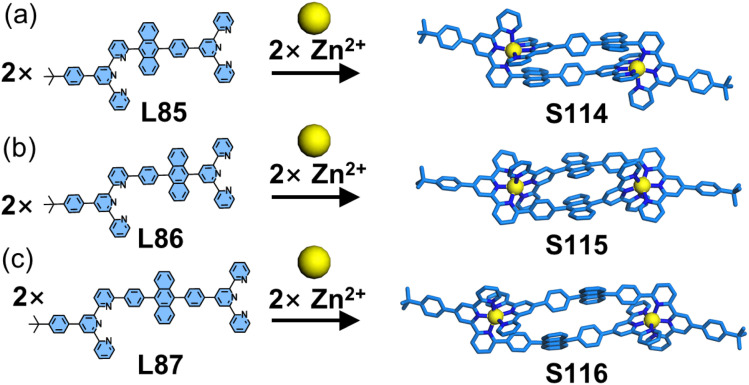
(a) Crystal structure of dimer S114 assembled from ligand L85. (b) Crystal structure of dimer S115 assembled from ligand L86. (c) Crystal structure of dimer S116 assembled from ligand L87.

Inspired by Lehn's work on mononuclear complexes based on 6,6′′-substituted tpy, Wang and coworkers developed a polynuclear decker supramolecular assembly strategy to achieve densely stacked arrangements of functional groups.^155^ A binuclear complementary coordination dimer system was developed using three dissymmetrical ditopic ligands (L85–L87) integrated with an anthracene chromophore. By varying the phenyl spacer in the ligand design, the resulting head-to-tail dimers (S114–S116) exhibited distinct spatial arrangements of the anthracene units (Fig. 44). SCXRD confirmed the formation of stacked decker structures with proximal chromophore alignment, demonstrating potential control over interchromophoric interactions and properties.

**Fig. 45:**
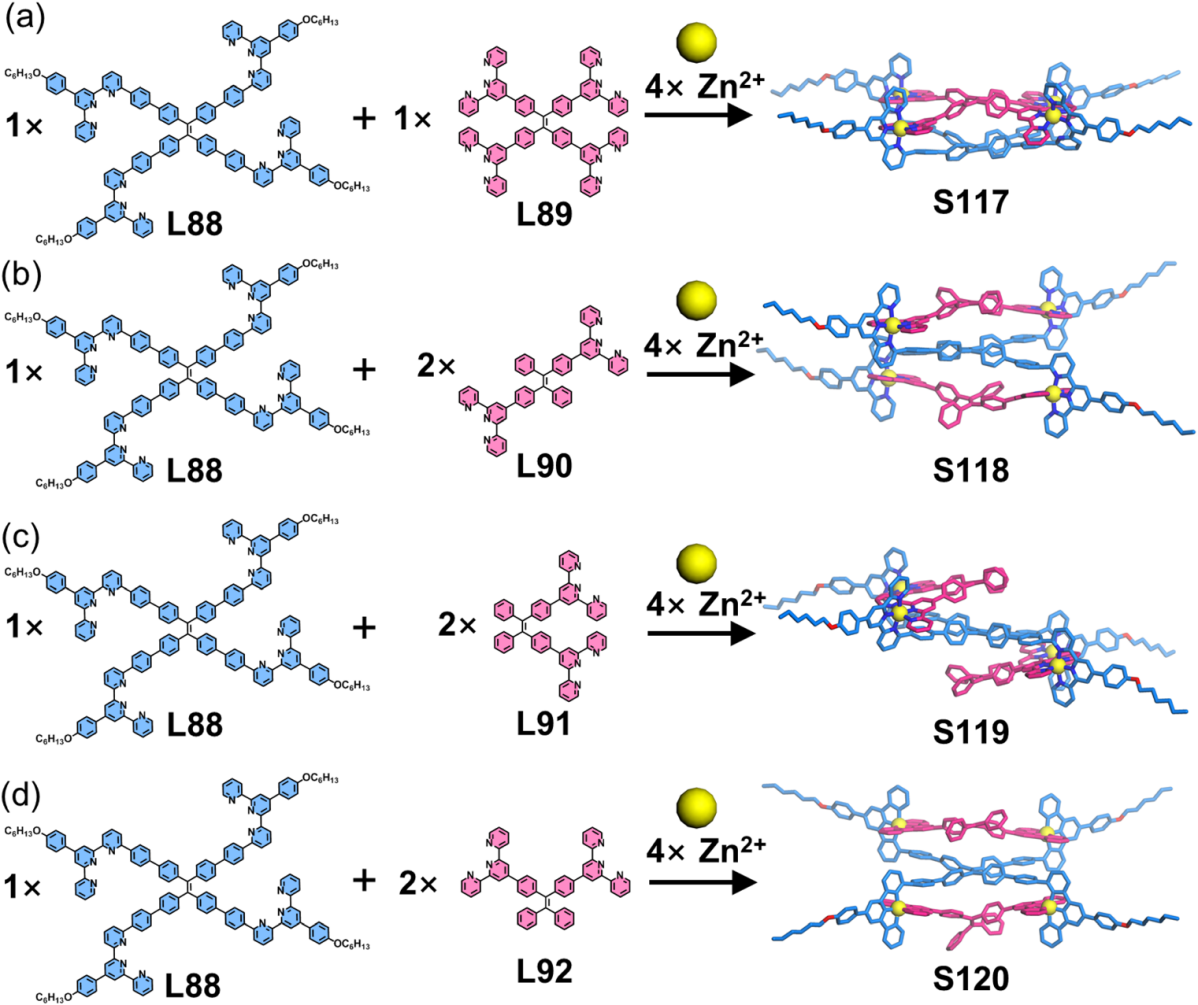
(a) Simulated structure of decker S117 assembled from ligands L88 and L89. (b) Simulated structure of decker S118 assembled from ligands L88 and L90. (c) Simulated structure of decker S119 assembled from ligands L88 and L91. (d) Simulated structure of decker S120 assembled from ligands L88 and L92.

**Fig. 46:**
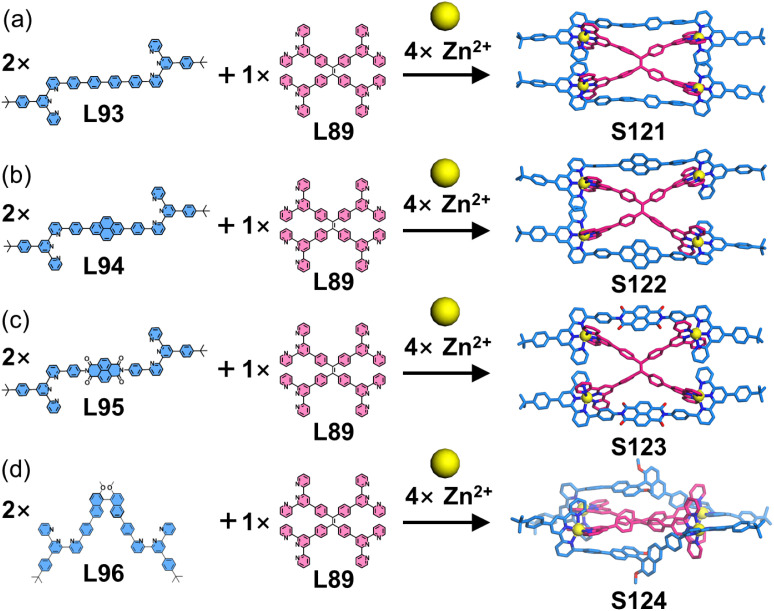
(a) Simulated structure of decker S121 assembled from ligands L93 and L89. (b) Crystal structure of decker S122 assembled from ligands L94 and L89. (c) Crystal structure of decker S123 assembled from ligands L95 and L89. (d) Crystal structure of decker S124 assembled from ligands L96 and L89.

The above strategy demonstrates that 6-position modification enables dense chromophore packing. However, such systems can only incorporate a single chromophore type, where the chromophores merely provide functional properties without conferring coordination selectivity. When two ditopic ligands with identical arm lengths but different chromophores are assembled with metal ions, a statistical mixture of homoleptic (AA/BB) and heteroleptic (AB) complexes forms, making it impossible to achieve further selectivity control through chromophore differentiation. To address this, the same group developed a heteroleptic assembly system. The design principle utilizes dissymmetrical multitopic ligands (A) exclusively functionalized at the 6-position of tpy and multitopic ligands (B) modified at the 4′-position of tpy. When co-assembled with metal ions, these geometrically matched ligands preferentially form heteroleptic AB architectures. This approach enables the synthesis of both homochromophoric and heterochromophoric decker structures. For instance, the claw-like tetraphenylethylene (TPE)-embedded ligand L88, when assembled with either tetratopic L89 or ditopic L90–L92 in the presence of Zn^2+^ ions, forms a double-decker (S117) or three triple-decker (S118–S120) architectures with 100% ligand selectivity, respectively (Fig. 45).^156^ Remarkably, this heteroleptic self-assembly strategy enables the construction of heterochromophoric decker systems by precisely positioning distinct chromophores on complementary ligands. This capability is further demonstrated by the assembly of claw-like ditopic ligands L93–L96 with L89 and Zn^2+^ ions, yielding the triple-decker complexes S121–S124 with programmed chromophore arrangements (Fig. 46).^42^

## 4 Functional metallo-supramolecular architectures

In the previous chapter, we systematically elucidated the unique advantages of the side-pyridine modification strategy in constructing tpy-based coordination supramolecular architectures. This strategy not only significantly expands the structural diversity of metallo-supramolecular systems but also achieves precise assembly control that is difficult to realize through conventional 4′-position modifications. More importantly, the side-pyridine modification endows the structures with novel performance characteristics by reconstructing the supramolecular framework and introducing additional functionalization sites. The following chapter will thoroughly explore the outstanding contributions of this strategy in functional expansion, with particular emphasis on revealing its regulatory mechanisms and performance advantages in the fields of luminescence, catalysis, chirality, host–guest chemistry, and hierarchical assembly.

### 4.1 Luminescence

**Fig. 47:**
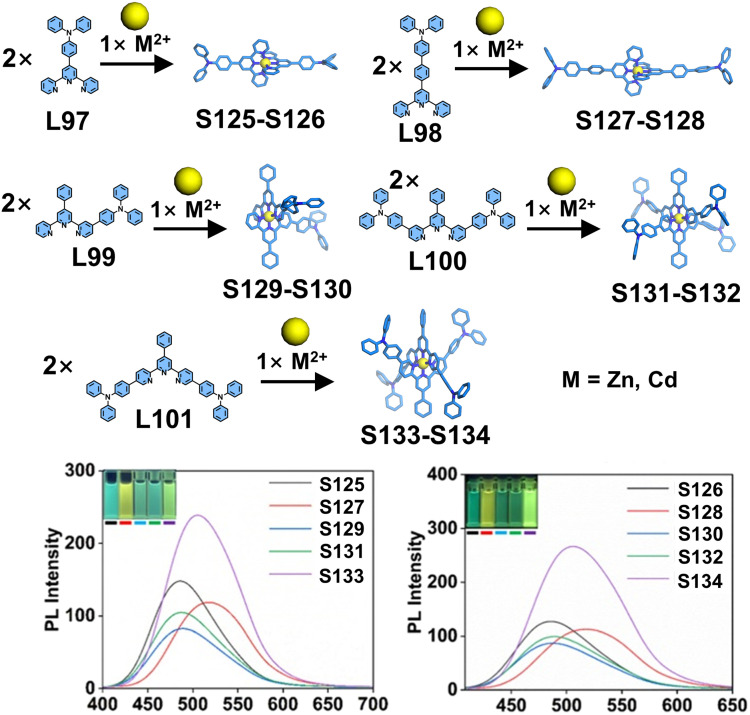
Simulated structures of complexes S125–S134 and their luminescent spectra in CH_3_CN.

**Fig. 48:**
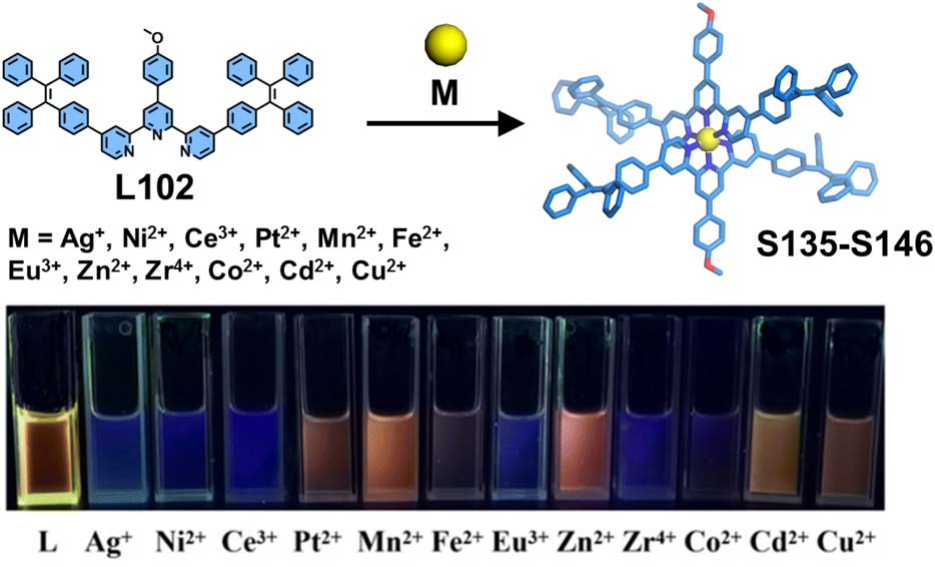
Self-assembly of complexes S135–S146 and the photographs under 365 nm UV light of L102 in CHCl_3_/MeOH (v/v, 1 : 3) with various metal ions.

The incorporation of luminophores into ligands represents a well-established strategy for constructing luminescent coordination supramolecular systems. While conventional 4′-position modifications of fluorescent groups are common, the side-pyridine modification approach not only alters supramolecular architectures but also modulates their photophysical properties by influencing interactions with the tpy moiety. Shi and coworkers systematically designed and synthesized a series of tpy-based mononuclear complexes (S125–S134) incorporating triphenylamine groups.^157^ Five distinct ligands—4′-substituted L97 and L98, 4-substituted L99, 4,4′′-substituted L100, and 5,5′′-substituted L101—were coordinated with Zn^2+^ and Cd^2+^ ions to form Zn^2+^ complexes (S125, S127, S129, S131, and S133) and Cd^2+^ complexes (S126, S128, S130, S132, and S134), respectively (Fig. 47). The strategic positioning of the electron-donating triphenylamine groups at different substitution sites of the electron-deficient tpy core significantly influenced intramolecular charge transfer (ICT) characteristics, consequently tuning their luminescence properties. Notably, the 5,5′′-substituted complexes S133 and S134 exhibited the highest fluorescence quantum yields in the solution state, demonstrating the superior efficacy of this modification strategy for optimizing luminescence efficiency. Based on a similar construction strategy, Xie, Wang, and coworkers reported a series of TPE-based mononuclear complexes (S135–S146). The TPE unit was functionalized at the 4,4′′ positions of the tpy moiety, yielding ligand L102. Upon coordination with different metal ions, these complexes exhibited distinct luminescent behaviors, making them suitable for use as fluorescent sensors for detecting heavy metal ions (Fig. 48).^43^

**Fig. 49:**
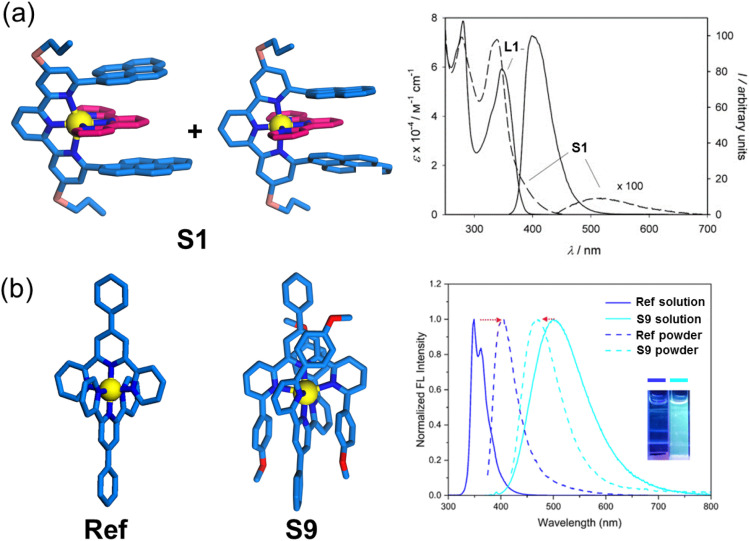
(a) Absorption and luminescent spectra of L1 and S1 in CH_2_Cl_2_. (b) Absorption and luminescent spectra of Ref and S9 in CH_3_CN and in the solid state, and the photographs of Ref and S9 in CH_3_CN under 365 nm are given in the insets.

Distinct from other substitution positions, the 6,6′′-position modification strategy enables close-range π-stacking between fluorophores, thereby effectively modulating luminescent properties. Lehn and coworkers investigated the photophysical behavior of heteroleptic complex S1, where pyrene units were strategically installed at the 6,6′′ positions of the tpy.^91^ Upon metal coordination, this design yielded a unique pyrene-tpy-pyrene stacked architecture that facilitated enhanced charge transfer (CT) interactions between the pyrene donors and the tpy acceptor. Comparative analysis revealed that complex S1 exhibited a substantially red-shifted emission profile (Δ*λ* > 120 nm) accompanied by reduced fluorescence quantum yield relative to its precursor ligand L1. This phenomenon clearly demonstrates how positional control of fluorophore arrangement can dramatically alter both the spectral characteristics and fluorescence efficiency of coordination complexes through precise spatial organization of π-conjugated systems (Fig. 49a). Similarly, Zn^2+^ complex S9 also demonstrates characteristic charge transfer luminescence behavior.^93^ This system exhibits analogous photophysical phenomena to S1, where strategic positioning of donor–acceptor–donor moieties facilitates ICT transitions (Fig. 49b). In contrast, the unmodified complex Ref, which lacks an electron–donor modification for stacking with TPY, exhibits no charge transfer effect and weak luminescence.

**Fig. 50:**
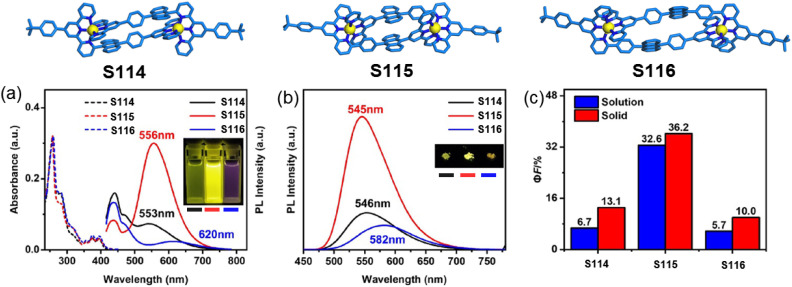
(a) Absorption and luminescent spectra of S114–S116 in CH_3_CN, and their photographs under 365 nm are given in the insets. (b) Luminescent spectra of S114–S116 in the solid state, and their photographs under 365 nm are given in the insets. (c) *Φ*_F_ of S114–S116 in CH_3_CN and in the solid state.

Wang and coworkers developed a series of decker architectures featuring controlled fluorophore stacking and systematically investigated their photophysical properties. Three dimeric decker complexes (S114–S116) constructed from 6-modified dissymmetrical ligands exhibited distinct anthracene packing arrangements, resulting in markedly different through-space electronic interactions (Fig. 50). Notably, while the precursor ligands (L85–L87) displayed similar emission characteristics, their corresponding dimers demonstrated dramatically divergent luminescent behaviors: (1) S115 with cofacial anthracene stacking exhibited bright yellow fluorescence (*Φ*_F_ = 32.6%); (2) S116 with offset stacking showed significantly red-shifted and quenched emission (*Φ*_F_ = 5.7%); (3) S114 with maximally separated anthracenes likely manifested weakest anthracene–anthracene interactions, permitting dominant anthracene-to-tpy charge transfer. This study clearly establishes how subtle variations in intermolecular luminophore alignment can drastically modulate the photophysical output of supramolecular architectures, providing valuable design principles for luminescent material engineering.^155^

**Fig. 51:**
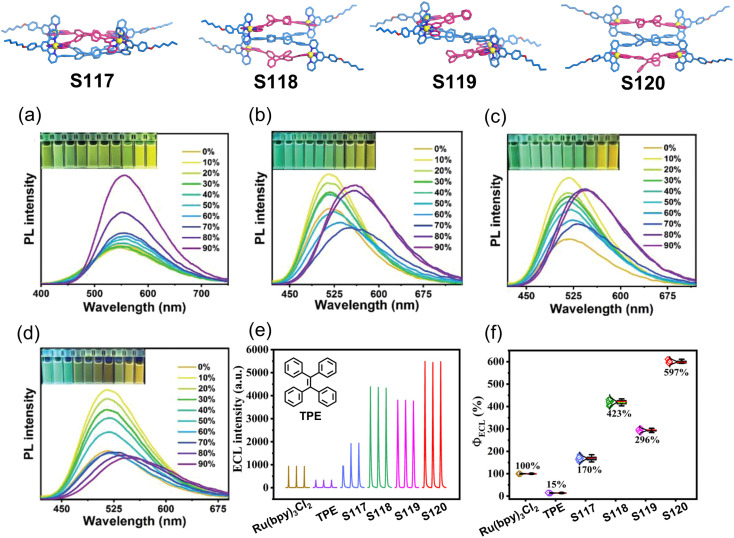
Luminescent spectra of (a) S117, (b) S118, (c) S119, and (d) S120*versus* the water fraction in the CH_3_CN/water, and their photographs under 365 nm are given in the insets. (e) ECL intensity–time curves and (f) ECL efficiencies obtained for 1 mM Ru(bpy)_3_Cl_2_, TPE, S117–S120 in phosphate-buffered saline containing 0.1 M K_2_S_2_O_8_.

Aggregation-induced emission (AIE), first reported by Tang and coworkers in 2001, addresses the notorious aggregation-caused quenching (ACQ) problem of conventional conjugated molecules.^158–160^ As a classic AIE luminogen, TPE has been widely employed in luminescent material design. Wang and coworkers constructed a series of TPE-based decker supramolecular architectures (S117–S120) featuring intramolecular cofacial packing of TPE units (Fig. 51). The constrained phenyl ring rotation in these stacked structures effectively suppresses non-radiative decay pathways, leading to enhanced fluorescence in solution and well-preserved fluorescence in aggregated states. Remarkably, all four assemblies demonstrated substantially improved electrochemiluminescence (ECL) cathodic efficiencies compared to free TPE. Particularly, S120 exhibited exceptional ECL performance, enabling highly sensitive cysteine detection with a remarkable detection limit of 14.4 nM.^156,161^

**Fig. 52:**
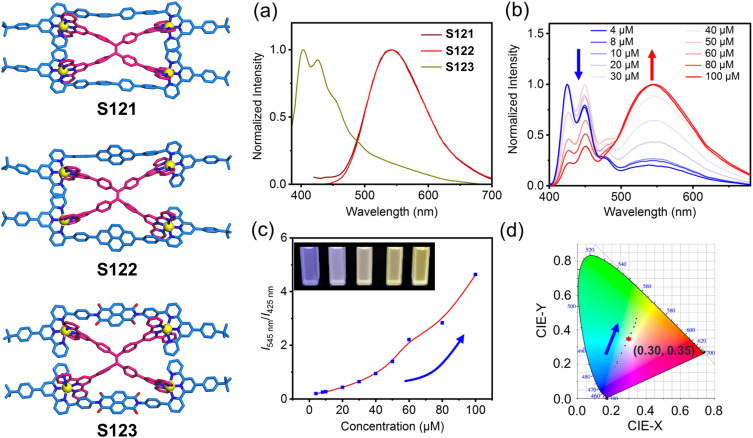
(a) Luminescent spectra of S121–S123 in CH_3_CN. (b) Luminescent spectra of S122 at different concentrations in DMSO. (c) Plot of relative luminescent intensity (*I*_545 nm_/*I*_425 nm_) of S122*versus* concentration in DMSO and the photographs at concentrations of 4, 10, 40, 60, and 100 μM under 365 nm are given in the insets. (d) CIE coordinates of the luminescence of S122 in DMSO at different concentration (4–100 μM).

Multichromophoric systems enable precise modulation of luminescent properties through controlled type, relative ratio, and spatial organization of chromophores. Building upon their stacked supramolecular platform, the same research group developed heterochromophoric decker architectures (S121–S123) and systematically investigated their interchromophoric interactions and photophysical behaviors (Fig. 52). The close spatial proximity between fluorophores enabled highly efficient Förster resonance energy transfer (FRET), resulting in quenching of the ditopic ligand's fluorescence. Concurrently, the stacking interactions effectively restricted the intramolecular rotation of the TPE units, leading to fluorescence enhancement of the TPE moiety. Taking advantage of the dynamic and reversible nature of metal–ligand coordination, concentration-dependent structural evolution in assemblies S121 and S122 was observed. Upon progressive dilution, the gradual disassembly of the supramolecular structures restored the characteristic blue fluorescence of the free ditopic ligand. Remarkably, at an optimal intermediate concentration, balanced emission from both ligands generated white light emission.^42^

### 4.2 Catalysis

**Fig. 53:**
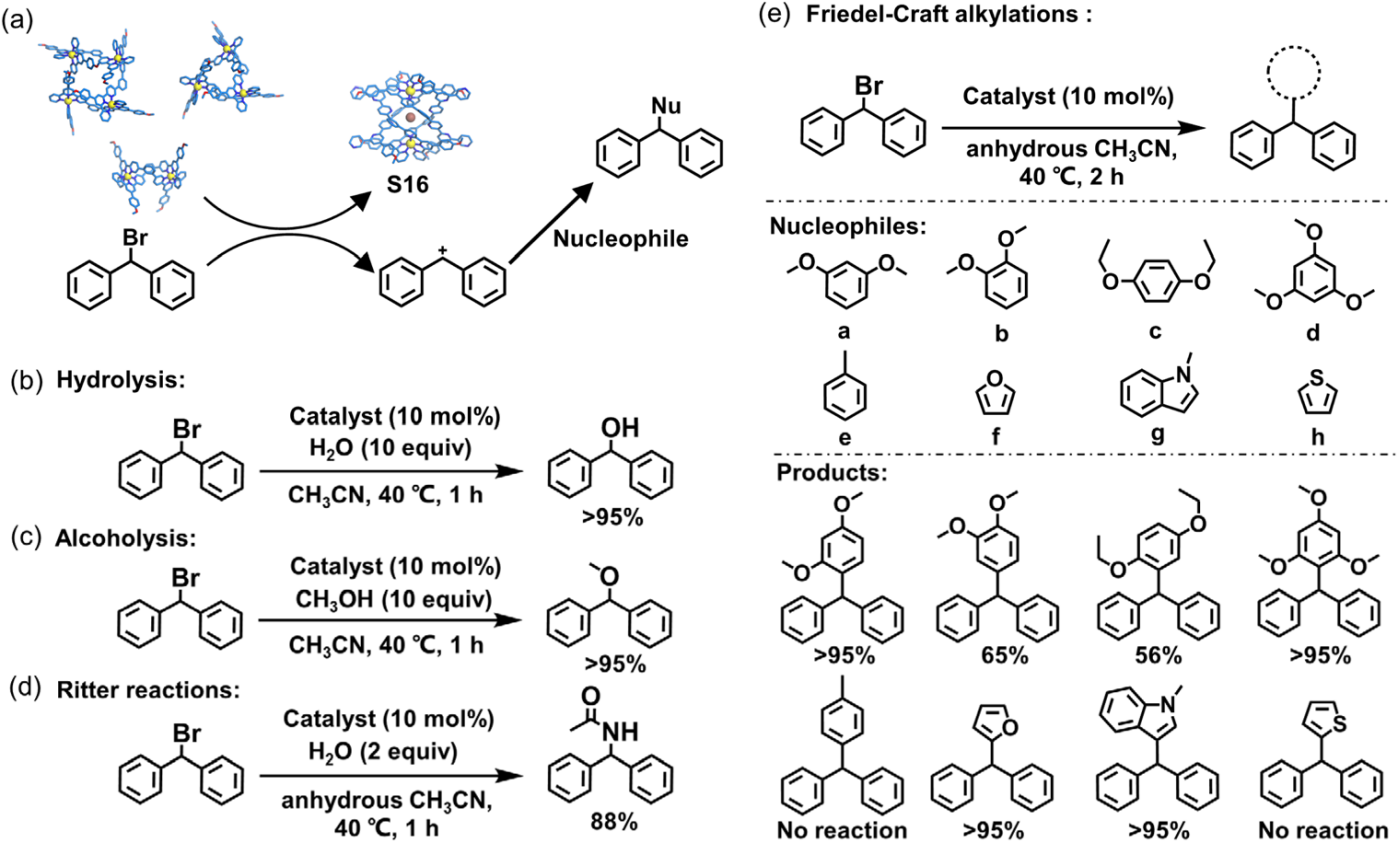
(a) The illustration of C–Br bond activation through the halophilic templation effect from macrocycles to helicate S16, and its catalysis of nucleophilic substitution reactions of bromo-hydrocarbons. The catalysis of (b) hydrolysis, (c) alcoholysis, (d) Ritter reactions, and (e) Friedel–Crafts alkylations reactions.

Wu and coworkers reported the halogen ion-templated synthesis of quadruple-stranded helicates (S15–S17). By leveraging the affinity between macrocyclic complexes and bromide anions (Br^−^), these complexes stabilize Br^−^ and facilitate the cleavage of the C–Br bond in (1-bromoethyl)benzene. The resulting Ph_2_CH^+^ acts as an ideal electrophile, undergoing diverse reactions with nucleophiles, including hydrolysis, alcoholysis, Ritter reactions, and Friedel–Crafts alkylations (Fig. 53). The authors extended the Friedel–Crafts alkylation reaction to arene and heteroarene substrates a–h. For the methoxybenzene substrates a, c, and d, although all exhibited good yields, only b and d showed high regioselectivity. Meanwhile, the steric hindrance of the ethoxy group in substrate c led to a reduced yield (56%). Heteroarene substrates f and g with higher electron density demonstrated high yields, while substrates e and h failed to undergo the reaction.^162^

**Fig. 54:**
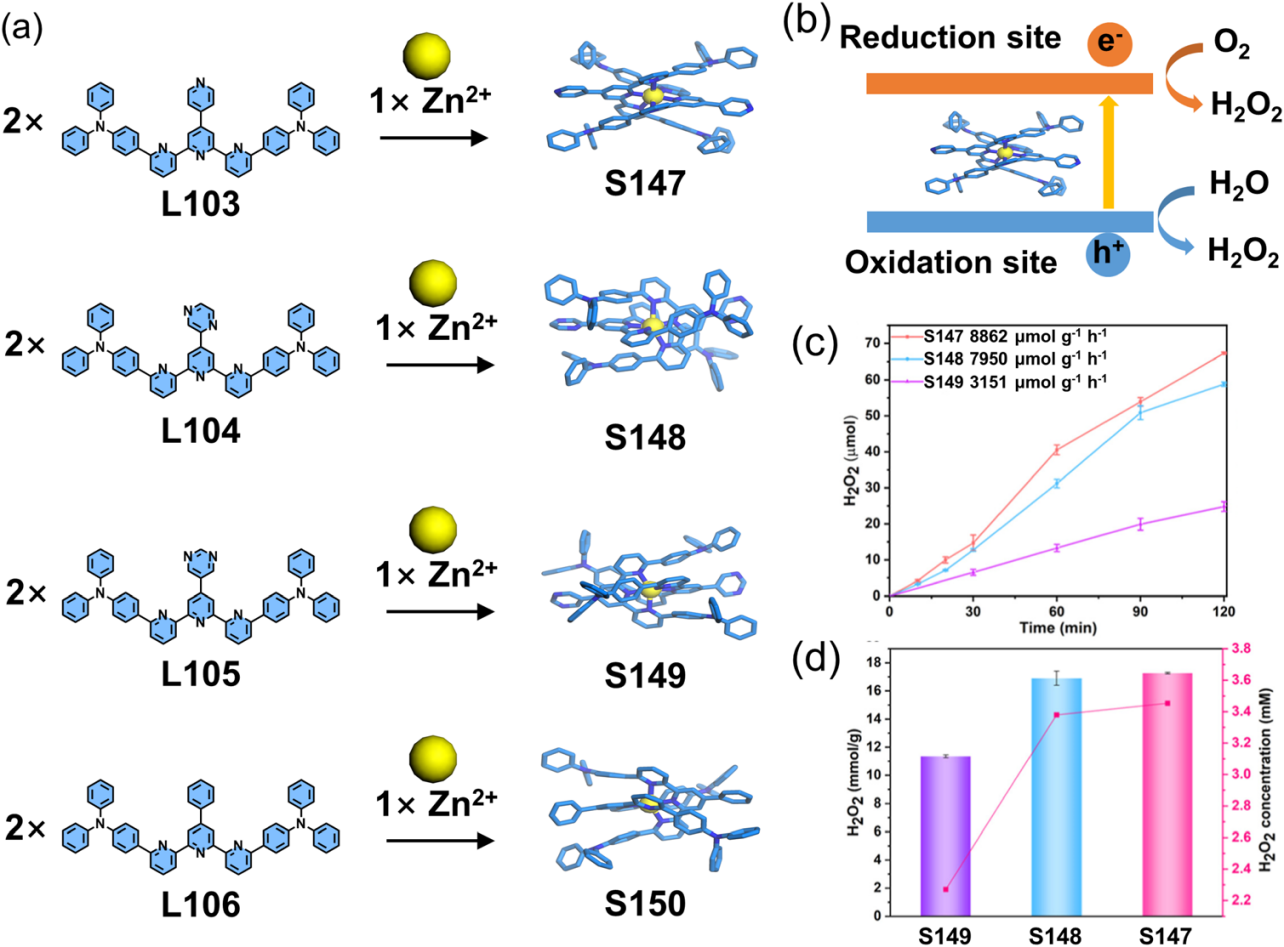
(a) Self-assembly of complexes S147–S150 (crystal structures). (b) The illustration of photosynthesis of H_2_O_2_. (c) Photocatalytic activities in H_2_O_2_ production and (d) levels of total H_2_O_2_ production in a pure water and air atmosphere.

Liu, Wang, and coworkers reported a series of donor–acceptor–donor (D–A–D) type mononuclear complexes (S147–S150). In these systems, the tpy core was functionalized at the 6,6′′ positions with electron-donating triphenylamine groups, while the 4′ position was modified with four distinct electron-accepting units, yielding ligands L103–L106. The 6,6′′-substitution pattern enforced proximal stacking between donor and acceptor moieties upon complexation, promoting through-space charge transfer (TSCT). This design generated long-lived excited states and spatially separated redox centers, enabling their application in photocatalytic hydrogen peroxide production with an exceptional evolution rate of 8863 μmol g^−1^ h^−1^ for S147 (Fig. 54).^27^

### 4.3 Chirality

Generally, chiral coordination supramolecular assemblies can be constructed through two primary strategies: (1) direct use of chiral ligands; (2) assembly from achiral ligands, where chirality arises from metal-induced ligand arrangements or ligand twisting.^163,164^ For tpy-based metallo-supramolecular systems, the incorporation of chiral moieties represents the prevailing design approach. This strategy proves effective for both traditional 4′-position and side-pyridine modification approaches.

**Fig. 55:**
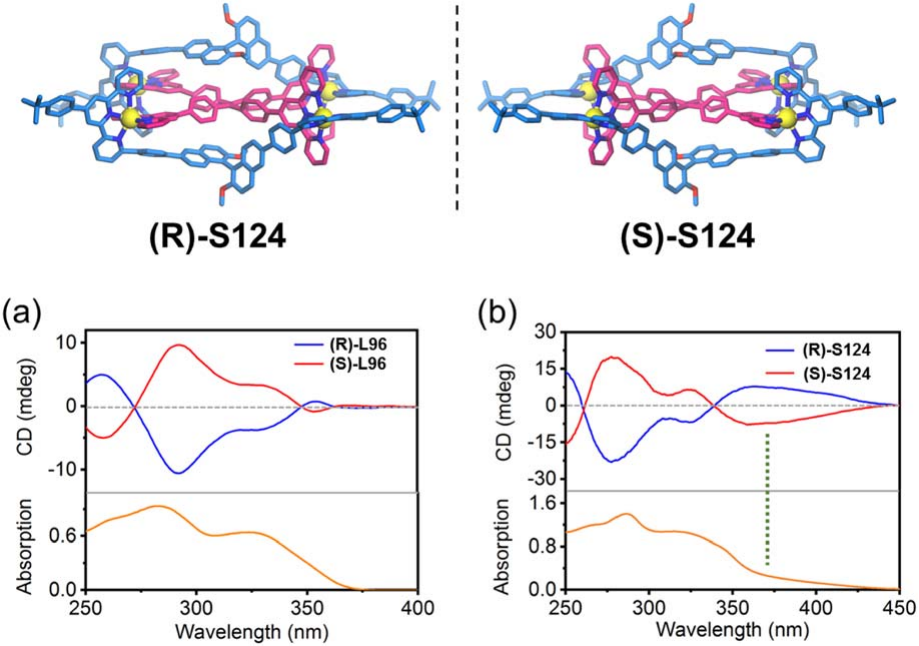
(a) CD spectra and absorption spectra of L96 enantiomers in CHCl_3_. (b) CD spectra and absorption spectra of S124 enantiomers in CH_3_CN.

For instance, Wang and coworkers designed a chiral ditopic ligand (L96) by introducing a binaphthyl group at the 6-position of tpy (Fig. 55). Assembly of L96, L89 and Zn^2+^ ions formed a chiral triple-decker architecture S124. The 6-position modification enforced intimate stacking between the chiral binaphthyl and TPE units, effectively restricting the chiral conformation of TPE. This spatial constraint facilitated successful chirality transfer, as demonstrated by SCXRD and CD spectroscopy.^42^

**Fig. 56:**
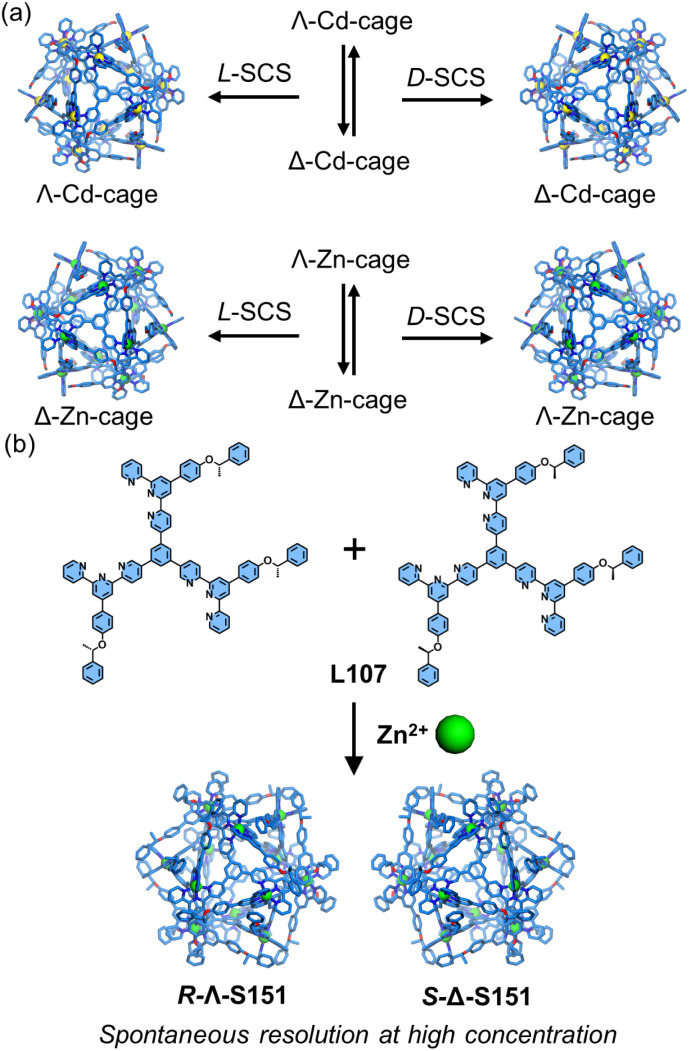
(a) The illustration of the same chiral guests (d- or l-SCS) inducing opposing chirality of cuboctahedra cages. (b) The illustration of the spontaneous resolution of cuboctahedra cage S151 at high concentration.

The highly symmetric 〈tpy-M-tpy〉 coordination geometry renders traditional 4′-position modifications ineffective for inducing chirality *via* metal-center directionality. Dissymmetrical functionalization of the side pyridines can create localized chirality around the metal center, reminiscent of the chirality generated by bidentate chelating ligands (such as bipyridine) with metal ions. This strategy is exemplified by helicates (S15–S17) and cages (S87–S91), which both exhibit stable chiral conformations (Fig. 56). Racemic Cd-cage S91 underwent resolution when treated with chiral guests (d- or l-sodium camphorsulfonate, d/l-SCS), yielding complex d-SCS⊂Λ-Cd-Cage or l-SCS⊂Δ-Cd-Cage. Notably, the Zn^2+^-based cage (Zn-Cage) displayed opposite handedness to Cd-Cage: l-SCS⊂Λ-Zn-Cage or d-SCS⊂Δ-Zn-Cage. To achieve spontaneous resolution, the authors synthesized chiral ligands (*R*/*S*-L107) by introducing chiral 2-methylphenylethyl groups. When assembled with Zn^2+^, the resulting cage (S151) spontaneously segregated into enantiopure forms (*R*-Λ-S151 or *S*-Δ-S151) at high concentrations or in the solid state.^68^

### 4.4 Host–guest chemistry

**Fig. 57:**
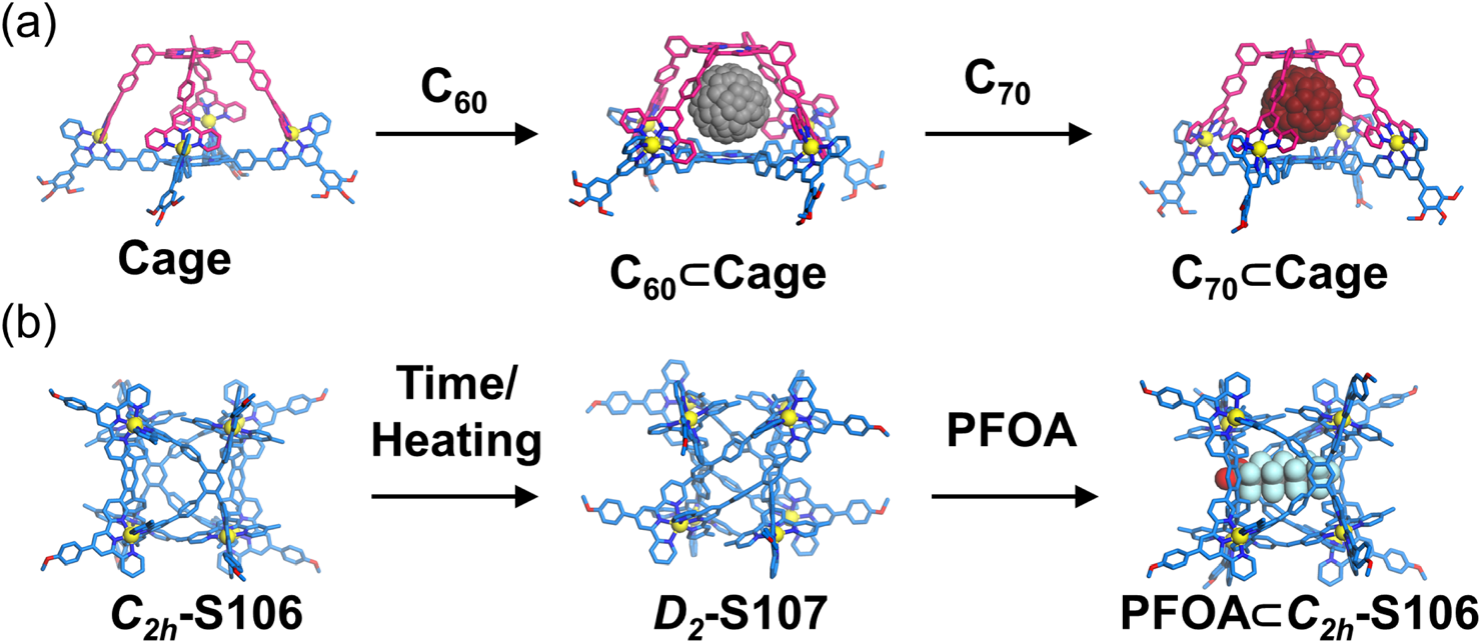
(a) The illustration of host–guest interactions of the porphyrinic cage with C_60_ and C_70_. (b) The illustration of conformational change of cages and the host–guest interactions with PFOA.

Due to their unique framework and cavity, 3D metallo-supramolecules are often employed as hosts to study host–guest behaviors. Compared with the at 4′-position modification, the 5-position modification can place the bulky 〈tpy-M-tpy〉 units at the corner of the structure, reducing the inhibition of host–guest encapsulation properties caused by steric hindrance. The dynamic cages (open-state S94 and closed-state S95) exhibit host–guest interactions with C_60_ and C_70_.^138^ Owing to the structural flexibility of the cage, its conformation—particularly the distance between porphyrin panels—can be adjusted to enhance donor–acceptor interactions with the guest molecules. Notably, the cage demonstrates a higher binding affinity for C_70_, enabling the selective displacement of pre-encapsulated C_60_ by C_70_, thereby achieving selective extraction of C_70_ (Fig. 57a). Wang, Wu, and coworkers also investigated the host–guest behavior of cubic isomers (*C*_2h_-symmetric S106 and *D*_2_-symmetric S107). The study revealed that *C*_2h_-S106 undergoes structural transformation into *D*_2_-S107, confirming that the *D*_2_-S107 is the thermodynamically stable product. However, upon the addition of the guest molecule perfluorooctanoate (PFOA) to *D*_2_-S107, a structural reversion is induced, leading to the formation of the host–guest complex PFOA⊂*C*_2h_-S106 (Fig. 57b).^137^

**Fig. 58:**
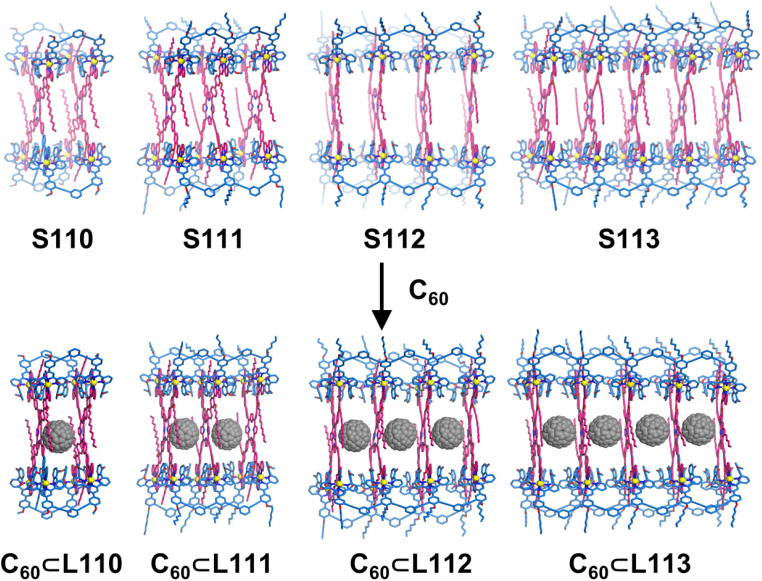
The illustration of host–guest interactions of cages S110–S113 with C_60_.

The capsules S110–S113 also exhibit host–guest behavior with C_60_. Due to their multiple cavities, S111–S113 can encapsulate multiple C_60_ molecules at a time (Fig. 58). However, upon binding multiple C_60_ guests, a negative synergistic effect was observed. This phenomenon likely arises from steric constraints: after initial C_60_ binding, the capsule undergoes conformational changes that reduce the effective size of the remaining cavities, thereby weakening further guest-binding affinity.^154^

### 4.5 Hierarchical self-assembly

**Fig. 59:**
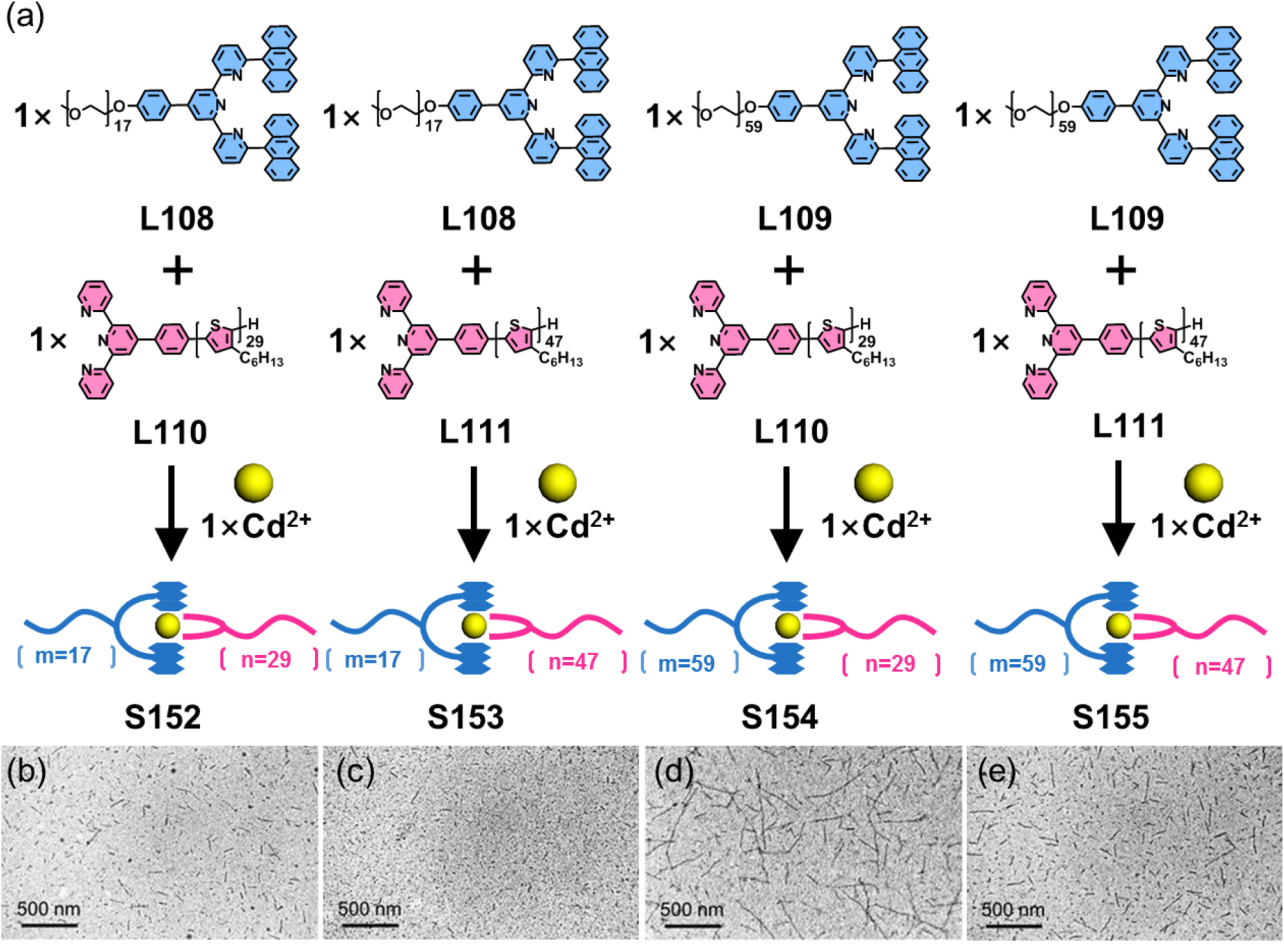
(a) Self-assembly of tpy-functionalized polymers S152–S155. TEM images of the solutions (3 mg mL^−1^, CHCl_3_/MeOH, v/v = 1 : 1) of (b) S152, (c) S153, (d) S154, and (e) S155.

The advantage of heteroleptic self-assembly lies in its capacity to integrate diverse ligands into a single assembly. By modifying different ligands with functional groups capable of providing additional non-covalent interactions, various functional components can be incorporated and subsequently utilized for hierarchical self-assembly into well-defined nanostructures. Building upon the complementary ligand pairs reported by Chan and coworkers, the same research group successfully constructed rod-coil block copolymers S152–S155 by incorporating hydrophilic poly(ethylene oxide) (PEO) and hydrophobic poly(3-hexylthiophene) (P3HT) chains. In chloroform/methanol mixed solvents, varying the rod-to-coil block ratios led to the formation of both spherical and fibrous nanostructures (Fig. 59). Notably, the morphological evolution from spherical assemblies to fibrous structures was observed with increasing P3HT content, demonstrating the precise control over hierarchical assembly architectures through block ratio modulation.^92^

**Fig. 60:**
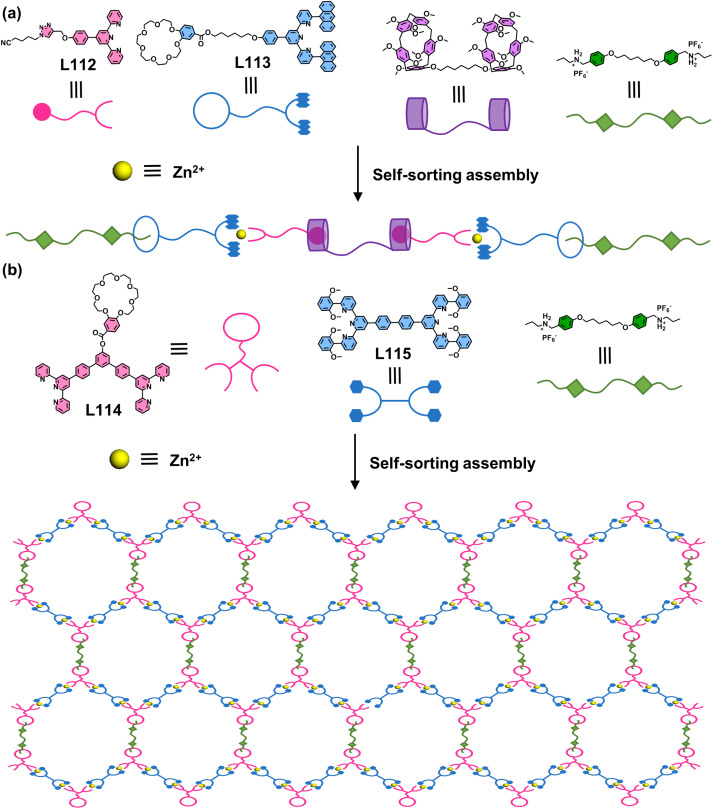
(a) Supramolecular linear polymers and (b) crosslinked polymer networks formed by hierarchical self-assembly.

Tian, Li, and coworkers demonstrated an elegant example of orthogonal self-assembly by synergistically combining host–guest interactions with metal coordination (Fig. 60a). In their design, complementary ligand pairs were functionalized with both a neutral guest chain (ligand L112) and a crown ether moiety (ligand L113), enabling the formation of bifunctional complexes. These complexes could subsequently co-assemble with monomers containing pillar[5]arene and dialkylammonium salt groups to form linear supramolecular polymers. TEM characterization revealed spherical morphologies, likely resulting from the entanglement of flexible supramolecular polymer chains. Notably, the dynamic nature of these polymers, conferred by their host–guest recognition motifs, allowed for reversible disassembly and reassembly through the competitive addition/removal of K^+^ ions and butanedinitrile molecules.^165^ Building upon this work, Tian, Li, and coworkers recently reported another orthogonal self-assembly system (Fig. 60b). By ingeniously integrating heteroleptic assembly of ligands L114, L115 and Zn^2+^ with crown ether-based host–guest interactions, they successfully constructed crosslinked polymer networks. These networks demonstrated remarkable gelation behavior at high concentrations, exhibiting intrinsic self-healing properties due to the dynamic nature of the non-covalent interactions involved.^166^

**Fig. 61:**
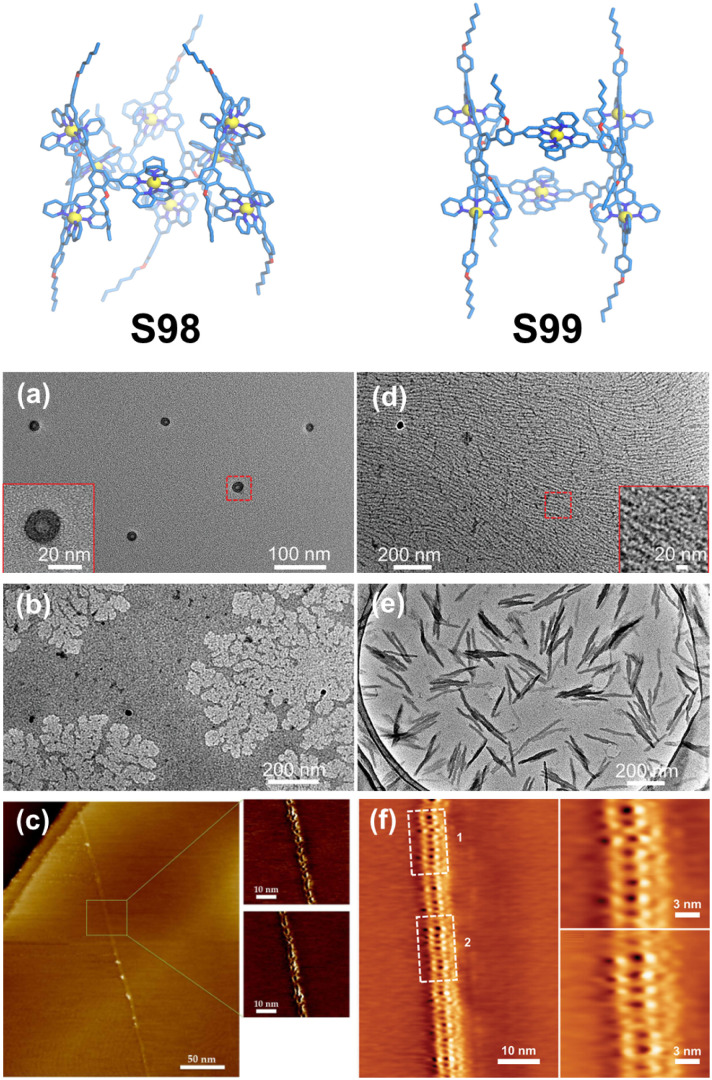
TEM images of (a) S98 and (d) S99, by evaporating THF into DMF. TEM images of (b) S98 and (e) S99, by evaporating diethyl ether into acetonitrile. STM images of (c) S98 and (f) S99 self-assembled as nanowire on HOPG surface.

Beyond introducing functional moieties capable of non-covalent interactions, the intrinsic structural features of assembly units alone can direct the formation of well-ordered hierarchical architectures. As exemplified by S98 and S99, these complexes demonstrate remarkable hierarchical assembly properties (Fig. 61). When prepared through THF evaporation into their DMF solution, S98 forms hollow structures while S99 assembles into nanofibers. Intriguingly, switching the poor solvent to diethyl ether leads to fibrous structures for S98 but irregular morphologies for S99. The researchers further investigated their hierarchical self-assembly behaviors at the liquid/solid interface using scanning tunneling microscopy (STM). Both S98 and S99 form extended nanofibers several hundred nanometers in length, with widths corresponding to single assembly units. The distinctive hourglass-shaped molecular structure likely facilitates these assemblies through enhanced intermolecular hydrophobic interactions and C–H⋯π interactions.^136^

## 5 Conclusions and outlook

Tpy has been extensively employed in constructing metallo-supramolecular architectures. However, modification strategies for tpy-based ligands have predominantly focused on the 4′-position of the central pyridine ring, with the primary challenge lying in how to diversify the structural and functional repertoire of tpy-based supramolecular systems.

In recent years, groundbreaking advances have been achieved in tpy-based metallo-supramolecular chemistry, particularly through side-pyridine modification strategies. This approach has garnered increasing attention due to its distinctive structural features, which enable richer functionality compared to traditional 4′-position modifications. Systematic analysis of existing research reveals that the key advantages of this strategy include facile modulation of supramolecular properties, tunable ligand geometry, dissymmetrical functionalization, and enhanced coordination selectivity. Modification at the 6,6′′-positions proves advantageous for improving coordination selectivity and constructing decker structures, while functionalization at the 4,5,4′′,5′′-positions is more commonly employed for regulating ligand geometry. Inspired by biological systems' precision, the development of dissymmetrical side-pyridine modification could mimic Nature's approach to creating functional complexity from simple building blocks. Dissymmetrical modification *via* one side pyridine facilitates the synthesis of low-symmetry architectures. Leveraging these features, researchers have successfully constructed a variety of novel supramolecular structures, including discrete mononuclear complexes, helicates, 2D metallacycles, 3D cages, and decker structures.

From a functional perspective, multiple modification sites on tpy enable the incorporation of functional moieties, such as chromophores and chiral groups, as discussed in this review. Side-pyridine modifications have also given rise to heteroleptic assembly systems, facilitating the integration of multiple functional components. Representative examples include multichromophore systems that enhance interchromophoric interactions and amphiphilic assemblies that achieve hierarchical organization. Additionally, this review summarizes the host–guest chemistry enabled by 3D structures with well-defined cavities.

In recent years, although this novel modification strategy has achieved significant progress in the construction and properties of tpy-based metallo-supramolecules, numerous challenges remain. Here, we briefly outline some future perspectives.

(1) A broader range of functional groups can be introduced at the 6,6′′ positions of tpy to enhance coordination selectivity, including both ligand and metal selectivity. Current studies have demonstrated that 6,6′′-position modifications can effectively improve ligand coordination selectivity and have been successfully extended to more complex heteroleptic architectures. However, the variety of applied functional groups remains limited. Moreover, recent studies have reported one-pot systems enabling selective coordination of two distinct metal species, yet such systems still require further development.

(2) The current decker architecture enables precise and controllable stacked arrangement of functional groups. The universality can be further expanded to fully exploit inter-group interactions for functional enhancements. Functional groups such as photoswitches, near-infrared photosensitizers, and organic radicals can be introduced in future studies to explore applications in sensing, imaging, stimuli-responsive materials, and biomedical fields.

(3) One-side pyridine modification offers a facile dissymmetrical ligand design strategy, enabling the synthesis of low-symmetry structures with distinctive cavity geometries. Future research should focus more on the specific host–guest recognition properties of these architectures toward chiral substrates, exploring their potential applications in molecular recognition, separation, and confined catalysis.

(4) Compared with 4′-position modification of tpy, side-position modifications have significantly expanded the synthetic diversity of ligands, thereby broadening the scope of discrete topological architectures and functionalities achievable through coordination or self-assembly. Beyond these, this approach holds considerable promise for the rational design and construction of novel materials, including metal–organic frameworks (MOFs) and covalent organic frameworks (COFs). The integration of this strategy with other non-covalent interactions or coordination self-assembly systems based on non-tpy ligands remains to be explored.

In this review, we aim to present a novel ligand design strategy for the field of coordination supramolecular chemistry, bringing multifunctional tpy into the spotlight. We envision that future studies on side-modified tpy will deepen our understanding of their unique assembly behaviors and enable the rational design of functional supramolecular systems. The strategic incorporation of diverse substituents at specific positions is expected to yield unprecedented architectures, where the synergy between multiple functional groups (*e.g.*, chromophores, chiral units, photoswitches, catalytic centers, and recognition motifs) can generate emergent properties. Such advances will likely propel applications in separation, sensing, and targeted drug delivery, where tpy-based cages may achieve selective molecular encapsulation, or in catalysis and optoelectronic devices leveraging interactions between precisely arranged chromophores.

## Author contributions

All authors contributed to composing the review and preparing the figures.

## Conflicts of interest

There are no conflicts to declare.

## Data Availability

No primary research results, software or code have been included and no new data were generated or analysed as part of this review.
